# Integrated Single-Cell and Spatial Multi-Omics of Clonal Precursors and Immune Niches in Germinal Center Lymphomas

**DOI:** 10.3390/cancers18071122

**Published:** 2026-03-31

**Authors:** Sofía Huerga-Domínguez, Beñat Ariceta, Paula Aguirre-Ruiz, Patxi San Martín-Uriz, Sarai Sarvide, Álvaro López-Janeiro, Diego Alignani, Aitziber López, Teresa Ezponda, Rocío Figueroa, Carlos Grande, Ana Alfonso, Esther Pena, Santiago Browne, Ramón Robledano, Amaia Vilas-Zornoza, Sergio Roa, Jose Ángel Martínez-Climent, Felipe Prósper, Miguel Canales

**Affiliations:** 1 Hematology and Cell Therapy Service, Cancer Center Clínica Universidad de Navarra (CCUN), Instituto de Investigación Sanitaria de Navarra (IdiSNA), 31008 Pamplona, Spain; shuergad@unav.es (S.H.-D.);; 2 Centro de Investigación Biomédica en Red de Cáncer (CIBERONC), 28029 Madrid, Spain; 3 Hematology-Oncology Program, Centro de Investigación Médica Aplicada (CIMA), Cancer Center Clínica Universidad de Navarra (CCUN), Instituto de Investigación Sanitaria de Navarra (IdiSNA), 31008 Pamplona, Spain; 4 Department of Pathological Anatomy, Clínica Universidad de Navarra, 31008 Pamplona, Spain

**Keywords:** follicular lymphoma, diffuse large B-cell lymphoma, histological transformation, single-cell RNA sequencing, single-cell DNA sequencing, spatial transcriptomics, tumor microenvironment

## Abstract

**Background**: Follicular lymphoma (FL) and diffuse large B-cell lymphoma (DLBCL) exhibit substantial heterogeneity, reflecting the diversity of the germinal center (GC). Histologic transformation of FL to DLBCL is associated with poor prognosis, yet robust biomarkers predicting transformation remain limited. **Methods**: We integrated single-cell DNA sequencing, single-cell RNA sequencing, and spatial transcriptomics in diagnostic lymph-node biopsies from non-transformed FL (ntFL), transformed FL (tFL), and DLBCL to characterize clonal states and immune niches in GC lymphomas. T-cell signatures associated with transformation were evaluated in an independently published single-cell FL dataset. **Results**: Transcriptional profiling revealed similarities between tFL and DLBCL, consistent with a GC-related malignant program. The tFL microenvironment showed enrichment of exhausted CD4^+^ regulatory and CD8^+^ effector T cells, together with CD4^+^ follicular helper T cells (Tfh) displaying an adhesion-related phenotype. Spatial analysis suggested increased proximity of exhausted/immunosuppressive T cells and enhanced Tfh-B-cell interactions in tFL compared with ntFL. These immune signatures were also observed in an external cohort and were associated with early transformation. In addition, clonal hematopoiesis-associated mutations were detected in microenvironmental cells across samples, suggesting a potential contribution to the lymphoma microenvironment. **Conclusions**: This work demonstrates the feasibility of integrating single-cell and spatial analyses in GC lymphomas and provides a framework for investigating tumor heterogeneity and immune organization. These findings may inform future studies on biomarker development and the rational design of immunotherapies.

## Simple Summary

Follicular lymphoma is a common type of lymphoma that can sometimes transform into a more aggressive disease called diffuse large B-cell lymphoma. This transformation is associated with poor clinical outcomes, but the factors driving this process remain unclear. In this study, we applied advanced technologies to analyze genetic alterations, gene expression patterns, and the spatial organization of cells within lymph-node samples from patients. This approach allowed us to study both malignant cells and the immune cells present in the tumor microenvironment. We identified specific immune cell states and interactions associated with lymphoma transformation and confirmed these findings in an independent dataset. Our results improve our understanding of how follicular lymphoma evolves and highlight potential biomarkers and immune mechanisms that could help predict transformation and inform future therapeutic strategies.

## 1. Introduction

Germinal centers (GCs) are dynamic and genomically unstable microstructures where B cells undergo somatic hypermutation and class switch-recombination [1], ultimately giving rise to memory B and plasma cells, making them essential for the humoral immune response [2]. However, the high proliferative and mutational activity intrinsic to GCs also underlines the origin of the two most common non-Hodgkin lymphomas (NHL): follicular lymphoma (FL) and diffuse large B-cell lymphoma (DLBCL). These GC-derived lymphomas are highly heterogeneous diseases, reflecting the molecular and transcriptional diversity of their tissue of origin. Despite their common origin, these entities arise from B cells at different stages of the GC reaction and display distinct molecular profiles and interactions with the tumor microenvironment (TME) [3].

Studies of FL and DLBCL have relied on bulk profiling, which has successfully identified recurrent mutations, chromatin remodeling defects, and transcriptional subtypes. However, bulk approaches average signals across heterogeneous populations, obscuring the diversity of malignant and immune cells as well as their spatial interactions within the lymph node. This is particularly relevant for FL, where the TME plays a fundamental role in disease biology and progression [4,5,6]. Histological transformation from FL to DLBCL is pivotal to clinical biology and provides leverage to study GC lymphomas as a spectrum [7,8,9]. For DLBCL, multiple genetic subtypes with distinct pathogenetic mechanisms have been described [10,11,12], yet the complexity of its TME remains incompletely understood [13].

Recent advances in single-cell RNA sequencing (scRNA-seq), single-cell DNA sequencing (scDNA-seq), and spatial transcriptomics have revolutionized our ability to dissect cancer biology. These technologies enable the simultaneous characterization of genetic alterations, transcriptional programs, and cellular neighborhoods within intact tissue sections [14,15,16]. Applied directly to patient biopsies, they contribute to uncover heterogeneity of malignant and non-malignant cells, reconstruct clonal evolution, and define immune niches in situ. Although, single-cell technologies have been used to investigate FL and DLBCL [17,18,19,20,21,22], the integration of several layers of information in the same samples represents a technical challenge.

Here, we integrated scDNA-seq, scRNA-seq, and spatial transcriptomics on diagnostic lymph-node biopsies from non-transformed FL (ntFL), transformed FL (tFL), GCB- and ABC-DLBCL, and on reactive tonsils. This exploratory, proof-of-concept multi-omics study is complemented by validation in an independent cohort. Our goal was to demonstrate how multi-omics profiling of patient-derived material can (i) reconstruct clonal architectures and identify GC-related precursors, (ii) reveal malignant transcriptional heterogeneity and developmental programs potentially involved in transformation, and (iii) map spatially organized immune niches associated with disease states. Together, these data illustrate the translational potential of multi-omics approaches to investigate lymphoma pathogenesis and to inform biomarker discovery and therapeutic strategies.

## 2. Results

**Figure 1:**
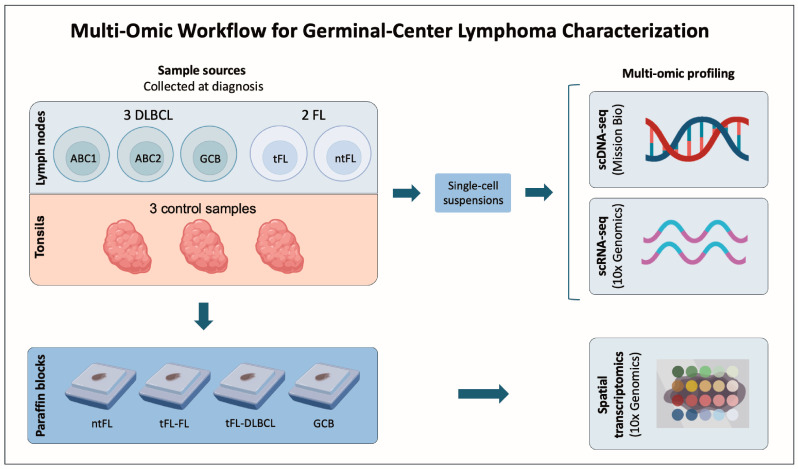
**Multi-omics workflow for germinal center lymphoma characterization.** Five diagnostic lymph-node samples were included, comprising three diffuse large B-cell lymphoma (DLBCL) cases (one germinal center B-cell-like [GCB] and two activated B-cell-like [ABC1 and ABC2] cases) and two follicular lymphoma (FL) cases. Among the FL patients, one patient experienced transformation to DLBCL 16 months after the diagnosis (tFL), while the other remained non-transformed (ntFL). Three tonsil samples from patients with tonsillitis were included as controls. All lymph-node and tonsil samples were mechanically dissociated into single-cell suspensions and analyzed by single-cell DNA sequencing (scDNA-seq; Mission Bio) and single-cell RNA sequencing (scRNA-seq; 10x Genomics). In parallel, high-resolution spatial transcriptomics (Visium HD, 10x Genomics) was performed on formalin-fixed paraffin-embedded (FFPE) tissue blocks from the ntFL sample, the tFL sample at diagnosis (tFL-FL) and at transformation (tFL-DLBCL), and the GCB DLBCL sample. This integrated multi-omics approach enabled the simultaneous characterization of clonal architecture, transcriptional heterogeneity, and spatial organization of malignant and immune cell populations across the germinal center lymphoma spectrum.

We analyzed diagnostic lymph-node biopsies from five germinal center-derived lymphomas using integrated single-cell and spatial transcriptomic approaches. The cohort included three DLBCL cases (one GCB and two ABC -ABC1 and ABC2-) and two FL cases. Among the FL patients, one experienced transformation to DLBCL 16 months after the diagnosis (tFL), while the other remained as FL (ntFL). Three tonsil samples from patients with tonsillitis were used as controls (Figure 1) (Table 1; Supplementary Table S1). This integrated analysis provided a unified view of the mutational landscape, transcriptional programs, and spatial organization of the TME.

### 2.1. scDNA-Seq Delineates Clonal Architectures and Reveals Contributions from Non-Malignant Compartments

Single-cell DNA sequencing and protein expression profiling enabled the reconstruction of clonal architecture across germinal center lymphomas. Genotyping data were obtained from 19,700 single cells in lymphoma samples (average of 3814 cells per sample; range: 2508–5247), and from 16,560 cells across control samples. Median sequencing coverage was 85 reads per amplicon per cell (IQR: 51–88). After filtering, 311 high-confidence variants were identified across the eight samples (Supplementary Tables S2 and S3; see Materials and Methods Section).

**Figure 2:**
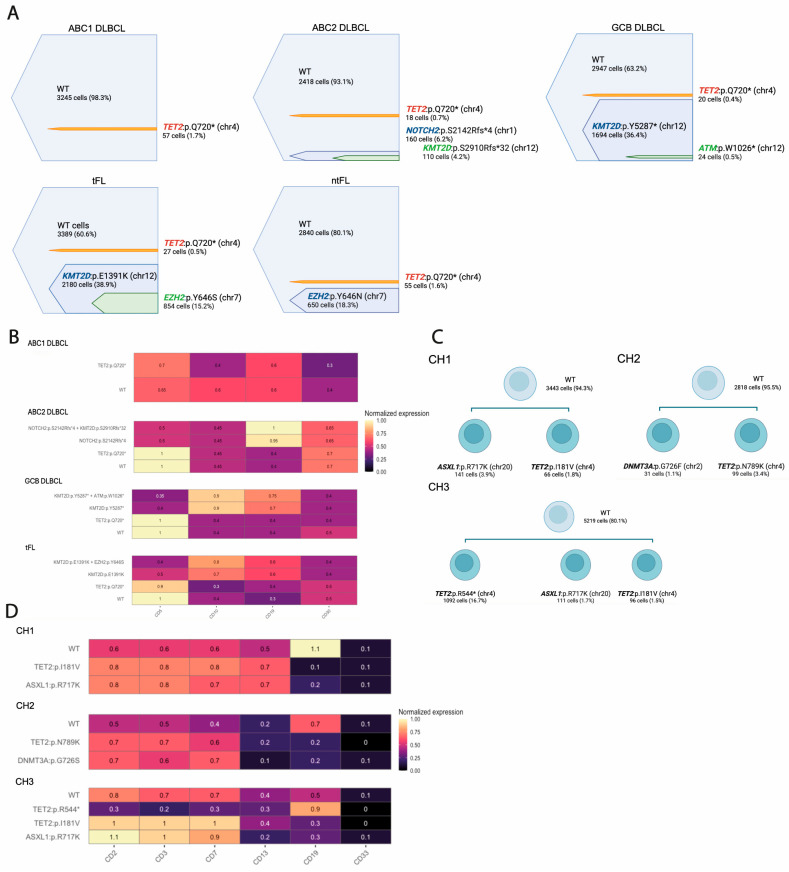
**Single-cell DNA sequencing (scDNA-seq) delineates clonal architectures and identifies clonal hematopoiesis (CH)-associated variants in germinal center lymphomas**. (**A**) scDNA-seq was performed on diagnostic lymph-node samples from five patients (ABC1, ABC2, GCB, tFL, and ntFL) and three control samples using the Mission Bio Tapestri platform with a lymphoma-focused custom panel. Shown are the frequencies of pathogenic somatic variants detected across all sequenced single cells per sample, color-coded by gene, in individual patient samples. Nested bars indicate subclonal variants arising within a founding clone. Detected variants include mutations in chromatin-modifying genes (*KMT2D*, *EZH2*), oncogenic drivers (*NOTCH2)*, and the tumor suppressor gene *ATM*. (**B**) Integration of genotype and surface protein expression revealed that *KMT2D*, *EZH2*, *ATM*, and *NOTCH2* mutations were localized within the CD19^+^ cells, whereas *TET2* mutations were confined to CD5^+^/CD19^−^ cells. Heatmap showing normalized expression of surface protein markers (CD5, CD19, CD10, CD30) across genetically defined single-cell clones identified by scDNA-seq. Rows correspond to genetic clones, and columns represent antibody-derived protein expression levels. Color intensity reflects normalized protein expression (scale shown on the right). (**C**) scDNA-seq identifies clonal hematopoiesis (CH)-associated subclones. scDNA-seq was performed on diagnostic lymph-node samples from three independent germinal center lymphoma cases (CH1, CH2, CH3) using the Mission Bio Tapestri platform with a commercial myeloid panel. The top node corresponds to wild-type (WT) cells, while lower nodes represent subclones harboring mutations in epigenetic regulators commonly associated with CH. Shown are the frequencies of somatic variants detected across all sequenced single cells per sample. (**D**) Integration of genotype and surface protein expression distinguishes clonal hematopoiesis-associated mutations from malignant B-cell clones. Heatmap showing normalized expression of surface protein markers (CD2, CD3, CD7, CD12, CD19, CD33) across genetically defined single-cell clones identified by scDNA-seq in CH1-CH3. Rows correspond to genetically defined clones, while columns represent antibody-derived protein expression levels. Color intensity reflects normalized protein expression (scale shown on the right). CH-associated variants show high expression of T-cell-associated markers (CD2, CD3, CD7) and low or absent CD19 expression, consistent with CH-associated variants being present in non-malignant immune compartments.

Four patients (ABC2, GCB, tFL, and ntFL) harbored mutations in chromatin-modifying genes (*KMT2D* and *EZH2*), oncogenic drivers (*NOTCH2*), and the tumor suppressor gene *ATM*, with variant frequencies ranging from 0.5% to 38.9% of all sequenced single cells per tumor sample. Based on these frequencies, we inferred likely founding events in each case: *KMT2D* variants in GCB and tFL, an *EZH2* mutation in ntFL, and a *NOTCH2* mutation in ABC2. Subclonal variants were found as secondary hits, including *EZH2* in tFL, *ATM* in GCB, and *KMT2D* in ABC2 (Figure 2A). These profiles suggest that ABC2 may align with cluster 1/BN2, while GCB may associate with cluster 3/EZB.

Low-frequency *TET2* variants were also detected. A nonsense mutation in *TET2* (p.Q720* C/T) was detected in all lymphoma samples and in one hyperplastic tonsil, with frequencies in the range of 0.4–1.7% of cells. No mutations were found in the other two control tonsils. Protein expression analysis revealed that *KMT2D*, *EZH2*, *ATM*, and *NOTCH2* mutations were localized within the CD19^+^ cells, whereas *TET2* mutations were confined to CD5^+^/CD19^−^ cells (Figure 2B). Their mutual exclusivity suggests that *TET2* mutations did not arise in B cells destined for lymphoma transformation but rather reflect clonal hematopoiesis (CH).

Given the identification of CH-associated variants and the limited CH coverage of the initial lymphoma-focused panel, we sought to validate and extend these findings using a broader CH-specific approach. Because sufficient material from the original cohort was not available for additional scDNA-seq analyses, and to ensure that these observations were not sample-specific, CH-associated mutations were therefore examined in an independent set of diagnostic lymphoma samples using a CH-focused scDNA-seq approach (Table 2; Supplementary Tables S4 and S5; see Materials and Methods Section).

All samples harbored two or three mutations in epigenetic regulators such as *TET2*, *ASXL1*, and *DNMT3A*, mostly single amino acid substitutions present at low frequencies (1.1–3.9% of all sequenced cells) (Figure 2C). These variants were detected in CD19^−^, CD3^+^, CD2^+^, and CD7^+^ cells, consistent with CH affecting non-malignant lineages. In contrast, a pathogenic nonsense *TET2* mutation, present in 16.7% of the cells, was localized in the CD19^+^ compartment, suggesting its acquisition within the malignant B-cell clone rather than reflecting CH (Figure 2D). These findings support the presence of CH-associated mutations in GC lymphomas, predominantly detected in T-cell populations of the tumor microenvironment, a feature that has been incompletely characterized to date [23,24]. Together, these results illustrate the utility of scDNA-seq applied to patient biopsies, enabling the simultaneous assessment of clonal architecture in malignant B cells and detection of CH signatures in non-malignant immune compartments that may be associated with the tumor ecosystem.

### 2.2. scRNA-Seq Reveals Malignant Heterogeneity and Immune Diversity Within Patient Samples

scRNA-seq of the same five lymphoma biopsies and three tonsillar controls revealed extensive malignant and immune heterogeneity within germinal center lymphomas. Tonsillar samples were included to support the identification of non-malignant cell populations and to enable the distinction between malignant and non-malignant cells. After quality control, 54,024 single cells from lymphoma samples (mean 10,805 per sample; range 7335–18,205) and 34,851 cells from controls were retained for analysis. Unsupervised transcriptomic clustering identified major cell compartments, including B cells, T cells, and myeloid populations. Annotation was based on canonical markers (*CD19*, *MS4A1*, *CD79A/B* for B cells; *CD3D*, *CD3E*, *CD3G*, *TRAC* for T cells; *LYZ*, *CD68*, *CD14*, *CD163* for myeloid cells).

**Figure 3:**
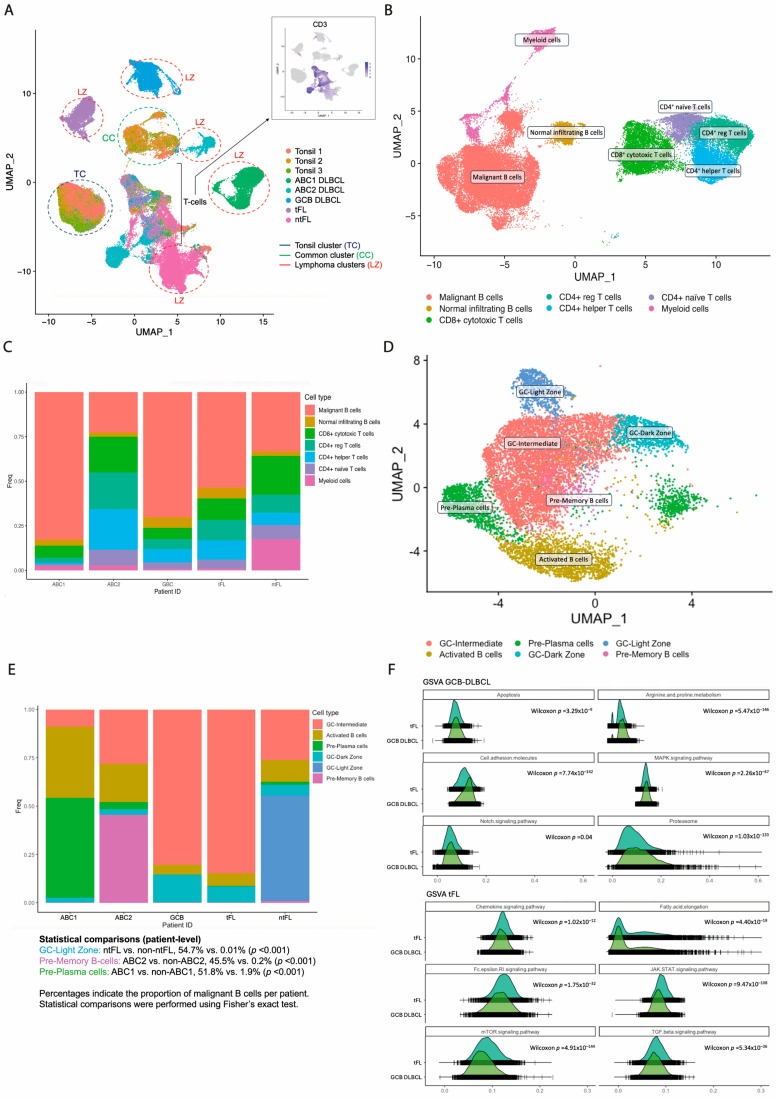
**Single-cell RNA sequencing (scRNA-seq) of germinal center (GC) lymphoma samples**. (**A**) Uniform Manifold Approximation and Projection (UMAP) of merged scRNA-seq data from five lymphoma samples (ABC1, ABC2, GCB, tFL, and ntFL) and three reactive tonsil controls. Cells are colored by sample of origin. Three major B-cell compartments were identified: a tonsil-specific cluster (TC), a shared cluster composed of cells from both lymphoma and tonsil samples (CC), and multiple patient-specific clusters (LC). Overlay of CD19 and CD3 expression was used to exclude T cells (bracketed region). CD19^+^ cells within the common cluster were considered non-malignant infiltrating B cells, whereas CD19^+^ cells within patient-specific clusters were classified as malignant B cells. (**B**) UMAP of the five lymphoma samples (ABC1, ABC2, GCB, tFL, and ntFL) showing seven transcriptionally defined clusters. (**C**) Bar plot representing the relative contribution of each cluster to each patient sample (ABC1, ABC2, GCB, tFL, and ntFL). Colors denote the seven clusters defined in (**B**). Percentages represent the proportion of each cluster within each patient. Statistical comparisons were performed at the single-cell level within individual samples using Fisher’s exact test; *p*-values reflect cell-level variability within samples. (**D**) UMAP of malignant B cells derived from the five lymphoma samples (ABC1, ABC2, GCB, tFL, and ntFL). Six distinct malignant B-cell subclusters were identified: intermediate germinal center (GC) cluster, dark-zone GC (DZ/GC) cluster, light-zone GC (LZ/GC) cluster, activated B-cell cluster, pre-memory B-cell cluster, and pre-plasma cell cluster. (**E**) Bar plot representing the relative contribution of malignant B-cell subclusters to each patient sample (ABC1, ABC2, GCB, tFL, and ntFL). Colors denote the six malignant B-cell subclusters defined in (**D**). Percentages represent the proportion of malignant B cells within each patient. Statistical comparisons were performed at the single-cell level within individual samples using Fisher’s exact test; *p*-values reflect cell-level variability within samples. (**F**) Gene set variation analysis (GSVA) comparing GCB DLBCL and tFL samples. GSVA scores for selected signaling and metabolic pathways are shown. Distributions are represented as density plots. Differences were assessed using a two-sided Wilcoxon rank-sum test at the single-cell level; resulting *p*-values (including FDR correction) reflect cell-level variability within samples.

Transcriptomic structure distinguished malignant from non-malignant B cells. As previously demonstrated, malignant B-cell clones exhibit distinct patient-specific transcriptional profiles, whereas normal infiltrating B cells, including those from tonsils, share a common transcriptional pattern [20,25]. Individual cells from the eight available samples (comprising five lymphoma and three control specimens) were merged. Three B-cell clusters were identified: (i) a cluster composed of tonsil cells (“tonsil cluster”), (ii) a second cluster comprising cells from all eight samples—both lymphoma and tonsils—(“common cluster”), and (iii) individual tumor-specific clusters (“lymphoma clusters”). Cells in the “common cluster” were identified as normal infiltrating B cells, whereas cells in the different “lymphoma clusters” were identified as malignant B cells (Figure 3A) (Supplementary Figure S1A). The presence of a shared “common cluster” across all samples indicates that infiltrating B cells retain a conserved transcriptional identity independent of disease state, whereas malignant B cells are defined by distinct, patient-specific programs.

Clonality was independently supported by immunoglobulin light chain restriction [20,26]. The light chain ratio per B cell (kappa/lambda) was calculated based on the expression of IGKC (κ light chain) and IGLC2/3 (λ light chain) genes. Consequently, clusters designated as “malignant B cells” exhibited exclusive light chain assignment (<1% or >91% Igκ/Igλ), while normal infiltrating B cells did not (Supplementary Figure S1B). Together, these findings validate the accurate discrimination of malignant and non-malignant B cells and provide a framework for downstream analyses of malignant heterogeneity.

Global clustering identified nine major cell populations (Figure 3B). Cycling populations were excluded for downstream analyses. The proportion of malignant B cells varied across patients, as illustrated in Figure 3C. The most prevalent cells in the TME were T cells (86.2% of TME cells), mostly CD4^+^ (63.4% of T cells). Myeloid cells were higher in the ntFL patient (13.6% vs. 1.6%; *p* = 0.003; reflecting cell-level variability within individual samples), whereas no other differences were detected in TME composition among patients.

Subclustering of malignant B cells revealed marked malignant heterogeneity, identifying six malignant and three non-malignant B-cell subsets, corresponding to distinct differentiation states: activated B cells (*CD83*, *MIR155HG*, *NFKB1*), germinal center (GC) B cells (*CCR7-*, *CD38+*, *PRDM1*), dark-zone (DZ) GC B cells (*CXCR4*), light-zone (LZ) GC B cells (*CD83*, *CD86*), memory B-cell precursors (pre-M) (*TNFRSF13C*, *MEF2B*, *RGS13*, *LRMP*, *CD22*, *RFTN1*), and plasma cells precursors (pre-PC) (*CREB3L2*, *ADM*, *TXNDC5*, *VDR*, *TNFRSF17*). Malignant subclusters comprised three germinal center B-cell clusters: (i) DZ signature, (ii) LZ signature, and (iii) intermediate cluster expressing both DZ and LZ markers (DZ/LZ); an activated B-cell cluster, a pre-memory B-cell cluster, and a pre-plasma cell cluster (Figure 3D). Non-malignant subclusters included an LZ-GC cluster, an intermediate DZ/LZ-GC cluster, and a pre-memory B-cell cluster (Supplementary Figure S1C).

Subcluster composition was heterogeneous across patients. Notably, the LZ GC B-cell subcluster was predominantly observed in the ntFL patient (54.7% vs. 0.01%; *p* < 0.001). In contrast, both tFL and GCB DLBCL samples showed a high proportion of intermediate DZ/LZ GC cells (84.7% and 80.5%), consistent with a shared GC-like transcriptional program at the single-cell level. ABC-DLBCL samples displayed distinct differentiation patterns: ABC2 was enriched in pre-memory B-cell cluster (45.5% vs. 0.2%; *p* < 0.001), whereas ABC1 was dominated by pre-plasma cells (51.8% vs. 1.9%; *p* < 0.001), in line with recent models of ABC-DLBCL ontogeny [18,26] (Figure 3E). Given the limited cohort size, these observations are presented descriptively at the individual patient level; *p*-values reflect cell-level variability and do not constitute statistical comparisons between disease groups.

While non-malignant B cells exhibited consistent gene expression profiles across patients, suggesting partial preservation of standard B-cell transcriptional programs (Supplementary Figure S1D), differential expression analysis further highlighted distinct transcriptional programs across malignant B-cell states. Intermediate DZ/LZ GC B-cell cluster upregulated genes involved in B-Cell Receptor (BCR) signaling, proliferation, and chromatin remodeling pathways (e.g., *CD79B*, *CREBBP*). The activated B-cell cluster displayed the activation of pro-tumorigenic pathways, including non-canonical NF-κB signaling (*CD40*, *NFKB1*) and enhanced translational activity (*HSPD1*, *RPL18A*). The pre-plasma cell cluster upregulated genes associated with lymphoma dissemination and tumor angiogenesis (*ITGB1*, *CDKN2A*). The DZ cluster exhibited an overexpression of *BTG1* and *BTG2*, suggesting p53-related DNA damage response, whereas the LZ cluster was enriched in immune-related genes, regulating cytokine and interferon signaling pathways (*STAT3*, *IRF7*). The pre-memory B-cell cluster displayed an upregulation of genes linked to immune regulation, and B-cell proliferation (*CD74*, *CD22*, *STAT3*, *JAK2*) (Supplementary Table S6).

Gene set variation analysis comparing tFL and GCB DLBCL samples at the single-cell level showed differences in pathway activity. GCB DLBCL cells were enriched for cell cycle dysregulation, cell adhesion, and MAPK/NOTCH signaling. In contrast, tFL cells showed a higher expression of immune-evasion-related pathways, involving JAK-STAT and TGF-β pathways (Figure 3F). Together, these findings delineate both intra- and intertumoral heterogeneity and are consistent with a model in which tFL and GCB DLBCL may share a GC-related precursor state characterized by BCR activation [27], with secondary alterations potentially contributing to divergent disease phenotypes. These transcriptional states complement the clonal architectures defined by scDNA-seq and provide a framework for subsequent spatial analyses.

### 2.3. The Functional Diversity of Tumor-Infiltrating T Cells Shapes the Immunological Landscape of FL and DLBCL

**Figure 4:**
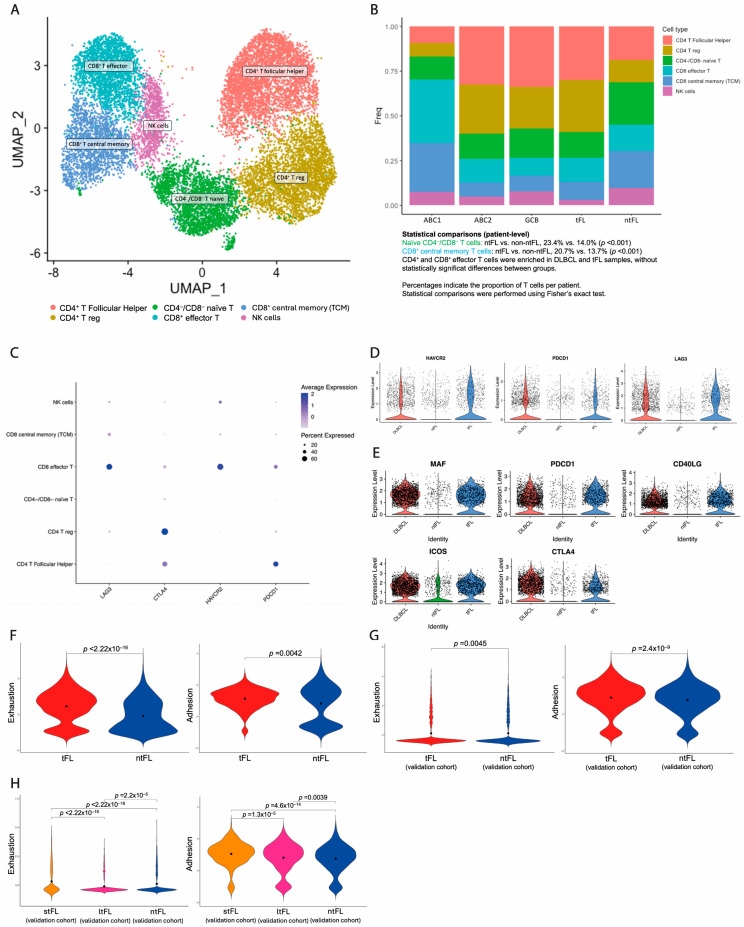
**T-cell heterogeneity across germinal center lymphomas.** (**A**) UMAP of T and NK cells derived from five lymphoma samples (ABC1, ABC2, GCB, tFL, and ntFL). Six immune subclusters were identified: CD4^+^ follicular helper T cells (Tfh), CD4^+^ regulatory T cells (Treg), CD4^−^/CD8^−^ naïve T cells, CD8^+^ effector T cells (Teff), CD8^+^ central memory T cells, and NK cells. (**B**) Bar plot showing the relative contribution of each T/NK-cell subcluster to individual patient samples (ABC1, ABC2, GCB, tFL, and ntFL). Colors correspond to the immune subclusters defined in (**A**). Percentages represent the proportion of total T/NK cells per patient. Statistical comparisons were performed at the single-cell level within individual samples (Fisher’s exact test); *p*-values reflect within-sample variability. (**C**) Dot plot showing the expression of immune checkpoint and exhaustion-associated genes (*LAG3*, *CTLA4*, *HAVCR2*, and *PDCD1*) across the identified T-cell subclusters. Dot color represents average normalized gene expression, while dot size indicates the proportion of cells expressing each gene within a given subcluster. (**D**) Violin plots showing the expression of exhaustion/immunosuppressive markers (*HAVCR2*, *PDCD1*, and *LAG3*) in CD8^+^ effector T cells and CD4^+^ regulatory T cells across samples (DLBCL, tFL, and ntFL). Differences reflect transcriptional variability at the single-cell level within individual samples and should be interpreted descriptively. (**E**) Violin plots showing the expression of adhesion-related genes (*CTLA4*, *MAF*, *PDCD1*, *CD40LG*, and *ICOS*) in CD4^+^ T follicular helper cells across DLBCL, tFL, and ntFL samples. Differences reflect transcriptional variability at the single-cell level within individual samples and should be interpreted descriptively. (**F**) Violin plots showing the exhaustion/immunosuppressive (CD8^+^ Teff and CD4^+^ Treg) and adhesion (CD4^+^ Tfh cells) signature scores in tFL and ntFL samples. Black dots indicate median values. Statistical comparisons were performed using a two-sided Wilcoxon rank-sum test at the single-cell level; *p*-values reflect cell-level variability within samples. (**G**) Violin plots depicting exhaustion/immunosuppressive (CD8^+^ Teff and CD4^+^ Treg) and adhesion (CD4^+^ Tfh cells) signature scores from an independent validation cohort, comparing tFL and ntFL. Black dots indicate median values. Statistical significance was assessed using the two-sided Wilcoxon rank-sum test. (**H**) Violin plots showing exhaustion/immunosuppressive (CD8^+^ Teff and CD4^+^ Treg) and adhesion (CD4^+^ Tfh cells) signature scores from an independent validation cohort, comparing short-term transformed FL (stFL), long-term transformed FL (ltFL), and non-transformed FL (ntFL) (stFL: transformation ≤ 12 months; ltFL: transformation > 12 months from diagnosis). Black dots indicate median values. Statistical significance was assessed using the two-sided Wilcoxon rank-sum test.

Tumor-infiltrating immune cells in the lymphoma microenvironment are highly diverse and influence tumor progression and therapy outcomes. T-cell reclustering resolved into six distinct subpopulations, defined by canonical marker expression. These included two CD4^+^ subclusters: follicular helper CD4^+^ T cells (CD4^+^ Tfh) (*CD4*, *CXCL13*, *TOX2*, *PDCD1*, *CXCR5*, *CD40LG*) and regulatory CD4^+^ T cells (CD4^+^ Treg) (*CD4*, *IL2RA*, *FOXP3*, *ICOS*, *TNFRSF1B*); two CD8^+^ subclusters: effector cytotoxic CD8^+^ T cells (CD8^+^ Teff) (*CD8A/B*, *GZMK*, *NKG7*, *PRF1*) and central memory CD8^+^ T cells (CD8^+^ TCM) (*CD8A/B*, *IL7R*, *CD44*, *ITGA1*); and one naïve CD4^−^CD8^−^ T-cell cluster (*SELL*, *CCR7*, *TCF7*, *LEF1*) and one NK cell cluster (*AOAH*, *SLAMF7*, *CD96*, *SAMD3*) (Figure 4A).

The distribution of T-cell subsets varied across patients. CD4^+^ and CD8^+^ Teff subclusters were more abundant in DLBCL samples. The tFL patient exhibited an expansion of CD4^+^ T cells, with similar proportions to those observed in the GCB DLBCL sample (59.1% and 58.9%). In contrast, the ntFL patient showed higher proportions of naïve CD4^−^CD8^−^ T cells (23.4% vs. 14.0%; *p* < 0.001) and CD8^+^ TCM cells (20.7% vs. 13.7%; *p* < 0.001) (Figure 4B). Given the limited cohort size, observations are descriptive; *p*-values reflect within-sample variability only.

Differential expression analysis across T-cell subclusters revealed distinct signaling programs. CD4^+^ Treg cells displayed immunosuppressive activity and transcriptional upregulation of genes linked to the IL-6/STAT3 signaling and the PD-1 pathway. The naïve CD4^−^CD8^−^ T-cell cluster upregulated genes involved in TGF-β signaling, a critical pathway for naïve T-cell differentiation into regulatory T cells [28]. Immune checkpoint receptors (*PDCD1*, *LAG3*, *HAVCR2/TIM-3*) were highly expressed in CD8^+^ Teff (Figure 4C). In CD8^+^ Teff, checkpoint co-expression is consistent with an exhaustion phenotype, whereas in CD4^+^ Treg it reflects an immunosuppressive state. CD8^+^ Teff and CD4^+^ Treg cells from DLBCL and tFL samples showed stronger exhaustion/immunosuppressive profiles compared to the ntFL sample (Figure 4D).

CD4^+^ Tfh cells were abundant across samples and exhibited a distinct transcriptomic profile [29], characterized by the overexpression of genes involved in adhesion between malignant B cells and CD4^+^ Tfh cells, such as *CTLA4*, *MAF*, *PDCD1*, *CD40LG*, and *ICOS*. Notably, a higher expression of these genes was observed in CD4^+^ Tfh cells from DLBCL and tFL samples compared to the ntFL sample (Figure 4E). These findings are consistent with prior evidence [19], linking BCR activation signatures to an “adhesion phenotype” in CD4^+^ Tfh cells.

Based on these overexpressed genes, we generated two transcriptional signatures: an exhaustion/immunosuppressive signature (assessed in CD8^+^ Teff and CD4^+^ Treg cells) and an adhesion signature (assessed in CD4^+^ Tfh cells). At the single-cell level, both signatures showed higher values in the tFL sample compared to the ntFL sample (exhaustion signature median: tFL 0.57 vs. ntFL −0.24; *p* < 2.22 × 10^−16^) (adhesion signature median: tFL 1.22 vs. ntFL 1.17; *p* = 0.0042) (Figure 4F). These *p*-values capture cell-level heterogeneity within individual samples and do not support between-group inferences. To validate these findings, we applied our signatures to an independent, previously published single-cell FL cohort [30]. Both signatures were significantly higher in FL patients who experienced transformation compared to those who did not (exhaustion signature median tFL 0.11 vs. ntFL −0.12; *p* = 0.0045) (adhesion signature median tFL 1.04 vs. ntFL 0.75; *p* = 2.4 × 10^−9^) (Figure 4G). Importantly, both signatures were also significantly higher in FL patients who experienced early transformation (stFL) compared to those who transformed later (ltFL) (exhaustion signature median: stFL 0.32 vs. ltFL −0.26; *p* = 1.3 × 10^−5^) (adhesion signature median: stFL 1.12 vs. ltFL 0.81; *p* < 2.22 × 10^−16^) (Figure 4H), supporting their association with early transformation. Overall, these findings suggest that T-cell functional diversity represents a key immunological layer within our framework. This layer integrates with the clonal architecture and malignant B-cell transcriptional programs and may contribute to the clinical differences observed across ntFL, tFL, and DLBCL.

### 2.4. Spatial Transcriptomics Defines Cellular Neighborhoods and Immune Niches

Spatial transcriptomic analysis revealed differences in the spatial organization of malignant and immune cell populations across the follicular lymphoma transformation spectrum. High-resolution spatial profiling of ntFL, tFL at diagnosis (tFL-FL), tFL after transformation (tFL-DLBCL), and GCB DLBCL paraffin blocks enabled the in situ characterization of tumor-immune organization (see Materials and Methods Section).

**Figure 5:**
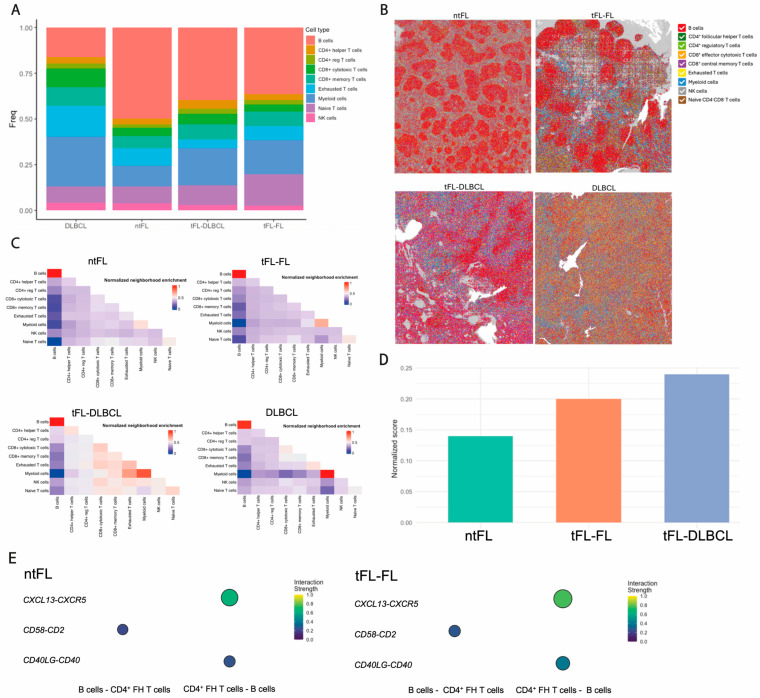
**Spatial transcriptomic analysis of lymphoma samples**. High-resolution spatial transcriptomics was performed on formalin-fixed paraffin-embedded (FFPE) tissue sections using Visium HD (10x Genomics). (**A**) Bar plot showing the number of inferred cell types per spatial bin across samples, reflecting the cellular complexity captured within individual 8 × 8 μm bins. (**B**) Representative spatial maps from ntFL, tFL prior to transformation (tFL-FL), tFL after transformation (tFL-DLBCL), and GCB DLBCL. Each bin is colored according to the dominant cell-type assignment based on scRNA-seq-derived gene signatures. (**C**) Bin-to-bin neighborhood enrichment analysis for ntFL, tFL-FL, tFL-DLBCL, and GCB DLBCL. Heatmaps depict normalized enrichment scores for spatial proximity between pairs of cell types, with higher values indicating preferential colocalization beyond random expectations. This analysis highlights spatial organization patterns and immune–tumor interactions across individual samples within the FL transformation spectrum. (**D**) Quantification of the spatial enrichment of exhausted/immunosuppressive CD8^+^ Teff and CD4^+^ Treg bins neighboring B-cell bins across ntFL, tFL-FL, and tFL-DLBCL samples. (**E**) Spatial ligand–receptor interaction analysis between CD4^+^ Tfh bins and B-cell bins in ntFL and tFL-FL. Circle size indicates interaction frequency, while color represents normalized interaction strength.

Cell-type mapping showed high concordance with expected cellular proportions (Figure 5A) and preserved tissue architecture across samples (Figure 5B). In ntFL, B-cell bins were predominantly surrounded by CD4^+^ Tfh and myeloid bins. In contrast, both tFL-FL and tFL-DLBCL samples exhibited B-cell bin neighborhoods enriched for exhausted/immunosuppressive T-cell populations (CD4^+^ Treg and CD8^+^ Teff) together with CD4^+^ Tfh bins, consistent with scRNA-seq findings. In GCB DLBCL, B-cell bins were most frequently neighbored by CD4^+^ Treg, CD4^+^ Tfh, and exhausted T bins.

Comparative neighborhood analysis suggested patterns consistent with remodeling of the tumor microenvironment along transformation. In ntFL, B-cell bins were more frequently surrounded by myeloid cell bins, including monocytes and granulocytes, whereas in tFL-FL, B-cell bin neighborhoods were enriched in CD8^+^ T-cell bins (effector and memory), exhausted T, and NK bins. Consistent with histopathological features, B-cell bins in DLBCL samples (tFL-DLBCL and GCB) exhibited fewer interactions with other B-cell bins and broader interactions with TME bins, reflecting the diffuse growth pattern. In contrast, FL samples (ntFL, tFL-FL) displayed B-cell bins forming enriched clusters with other B-cell bins (resembling follicular structures), alongside distinct neighborhoods enriched in T and myeloid bins (Figure 5C).

Quantitative spatial analysis suggested increased enrichment of exhausted/immunosuppressive CD8^+^ Teff and CD4^+^ Treg bins surrounding B-cell bins in tFL-FL compared to ntFL (Z-score 0.20 vs. 0.14), with a further increase following a transformation to DLBCL (tFL-DLBCL) (Z-score 0.24 vs. 0.20) (Figure 5D). Spatial ligand–receptor interaction analysis, focused on ntFL and tFL-FL samples, suggested enhanced interactions between CD4^+^ Tfh bins and B-cell bins in tFL-FL compared to ntFL, including CD40LG-CD40 (0.40 vs. 0.26), CXCL13-CXCR5 (0.76 vs. 0.65), and CD2-CD58 (0.27 vs. 0.22), consistent with the adhesion signature identified in CD4^+^ Tfh cells by scRNA-seq (Figure 5E). These spatial comparisons are descriptive; Z-scores do not support between-group inference.

Overall, these spatial analyses are consistent with our scDNA-seq and scRNA-seq findings, illustrating how mutationally defined malignant clones and transcriptionally distinct immune states are organized within tissue niches. This tri-modal framework suggests that clonal B cells reside within immunoregulatory neighborhoods enriched for exhausted T cells and adhesion-prone Tfh, providing a unified view of how genetics, transcriptional programs, and spatial architecture converge to shape FL and DLBCL.

## 3. Discussion

The current study demonstrates the feasibility of integrating scDNA-seq, scRNA-seq, and spatial transcriptomics in lymphoma samples to explore clonal precursors, malignant heterogeneity, and immune niches. It also highlights the potential of multi-omics approaches to investigate cellular interactions relevant to disease evolution. The integration of these technologies in this study suggests three main hypotheses: (i) tFL and GCB DLBCL may share transcriptional features consistent with a GC-related malignant program; (ii) tFL may harbor an immunoregulatory niche in which adhesion-phenotype Tfh cells and exhausted CD4^+^ Treg and CD8^+^ Teff cells are enriched and spatially proximate to malignant B cells; and (iii) spatial organization may contribute to shaping these interactions in situ. Transcriptional immune signatures derived from these observations were also identified in an independent single-cell FL cohort, where they were associated with early transformation.

A major limitation of this study is the small sample size and cross-sectional design, which preclude robust statistical comparisons and limit the generalizability of the findings. As a result, all observations should be interpreted as descriptive and hypothesis-generating rather than definitive evidence of disease-specific differences.

Single-cell profiling revealed marked intra- and intertumoral heterogeneity among malignant B cells within the analyzed samples. The observed transcriptional differences suggest that ntFL and tFL may display distinct biological features. The ntFL sample was enriched in LZ GC B cells, characterized by the overexpression of immune-related genes involved in cytokine and interferon signaling. This transcriptional pattern may partially reflect the influence of *EZH2* missense mutations, as identified in our scDNA-seq analysis. *EZH2* mutations enhance the repression of genes typically silenced in LZ centrocytes, regulating GC exit and terminal differentiation [3,31,32]. In addition, they impair the centrocyte recycling program [33]. Both mechanisms may contribute to LZ centrocyte expansion and lymphomagenesis.

In contrast, malignant B cells from the tFL and GCB DLBCL samples displayed transcriptional similarities, compatible with a potential shared precursor state [7,8], comprising intermediate DZ/LZ GC B cells and reflecting an active GC reaction with strong BCR signaling [34]. Holmes et al. associated this phenotype with the EZB DLBCL cluster [18], while Tsukamoto et al. reported enhanced BCR signaling in high-risk FL [35]. ABC-DLBCL samples preferentially engaged pre-memory and pre-plasma trajectories [3,18,36] and contained fewer GC-like states than FL and GCB DLBCL samples, suggesting entity-specific developmental patterns. These observations provide a framework for further investigation of cell-of-origin heterogeneity and its potential clinical implications.

The scDNA-seq data provide a plausible—albeit hypothesis-generating—genetic scaffold for these transcriptional programs. Early *KMT2D* mutations were detected in both tFL and GCB, in line with previous studies [7,25,37]. These mutations reduce H3K4 methylation at enhancer regions, repressing BCR signaling inhibitors and promoting BCR overexpression [3,31,37]. Together with the predominance of intermediate DZ/LZ GC B cells, these observations suggest a potential shared GC-related precursor for tFL and GCB DLBCL characterized by heightened BCR signaling. *EZH2* (tFL) and *ATM* (GCB) appear as secondary lesions with distinct pathway correlates (immune evasion versus cell cycle/DNA damage) and may contribute to the specific lymphoma phenotype. In our cohort, *ATM* mutations may contribute to GCB DLBCL through cell cycle disruption and DNA damage response mediated by MAPK [38,39], whereas *EZH2* mutations are associated with FL biology through enhanced immune evasion and metabolic regulation [40].

Additionally, our data suggest a dynamic and spatially organized interplay between malignant B cells and their immune microenvironment. The ntFL sample showed higher proportions of myeloid and naïve CD4^−^CD8^−^ T cells, whereas tFL and DLBCL samples were enriched for both CD4^+^ regulatory and CD8^+^ effector T cells with exhaustion features. The combined presence of immunosuppressive CD4^+^ Treg and exhausted CD8^+^ T cells—together with their spatial proximity to B-cell territories—was observed in samples with early transformation within this cohort. This pattern was also observed in an independent single-cell FL cohort [30] and supported by spatial transcriptomic analyses. A key finding was the high proportion of CD4^+^ Tfh cells and their spatial proximity to malignant B cells. These observations are consistent with prior studies suggesting that Tfh cells may support B-cell proliferation and survival, which may in turn reinforce Tfh differentiation pathways [41,42]. In Bcl-2 transgenic mice, CD4^+^ T-cell depletion results in reduced germinal center formation, supporting a role for CD4^+^ T cells in maintaining germinal center dynamics [43]. In both our cohort and the validation cohort, the adhesion-related genes [29,41,44,45,46,47] were upregulated in CD4^+^ Tfh cells from tFL, particularly in early transformed cases compared to ntFL. Spatial ligand–receptor analysis suggests enhanced Tfh-B-cell interactions in tFL. These findings point toward a potential association between this phenotype and early transformation.

Taken together, the expansion of CD4^+^ Tfh with an “adhesion phenotype”, along with immunosuppressive CD4^+^ reg T cells and exhausted CD8^+^ eff T cells, is consistent with a highly immunosuppressive niche. This network may represent a therapeutic vulnerability in GC lymphomas beyond traditional B-cell-directed approaches and may also have clinical relevance in the context of early transformation. Moreover, this deeper understanding of the TME may help inform future strategies for sequencing immunotherapy in clinical practice, as emerging evidence suggests these immune components may influence the efficacy of CAR-T-cell therapy and bispecific antibodies [17,48].

Beyond tumor cells and immune niches, we detected recurrent CH-associated mutations within non-malignant compartments. Specifically, *TET2*, *ASXL1*, and *DNMT3A* mutations were identified in CD19^−^, CD3^+^, CD2^+^, and CD7^+^ cells, suggesting that these alterations did not originate from the B-cell lymphoma clone. These findings indicate that CH-associated mutations may be present in non-malignant immune compartments and could be associated with microenvironmental alterations, as described in Hodgkin lymphomas [49,50]. Collectively, our data are consistent with the presence of CH-associated mutations in GC lymphomas, a feature that has been incompletely characterized to date [24,51]. While the presence of CH mutations in T cells raises the possibility of an association with GC lymphoma biology, including potential effects on B-cell proliferation and survival, further functional and longitudinal studies are required to determine their role in lymphomagenesis. Supporting this notion, follicular helper T-cell lymphomas (TFHLs), which frequently arise in the context of CH, often exhibit both polyclonal and monoclonal GC B-cell expansions. These B cells do not always harbor the same CH mutations as the TFHL clone, suggesting a Tfh-driven proliferative effect on B cells [52,53,54].

While mechanistic implications remain to be defined, these data suggest that CH may act as a potential modifier of the tumor–immune ecosystem. Given that some variant allele fractions approach platform detection limits, CH calls should be interpreted cautiously and validated functionally in future studies.

Building on this framework, this study demonstrates the feasibility of applying multi-omics technologies to diagnostic biopsies to explore clonal architecture and immune context. Larger longitudinal studies will be required to validate specific genetic or immune features as biomarkers of transformation risk. This work therefore provides proof-of-concept that a comprehensive multi-omics interrogation of patient samples is feasible and informative in a translational research setting. Beyond lymphoma, this approach illustrates how integrating single-cell and spatial analyses can refine our understanding of cancer biology by linking clonal genetics, transcriptional states, and spatial context. These insights may inform biomarker development and the rational design of immunotherapies.

## 4. Materials and Methods

### 4.1. Sample Collection and Single-Cell Preparation

Five lymph-node biopsies were collected: three DLBCL specimens (one GCB and two ABC: ABC1 and ABC2) and two FL specimens. One FL patient progressed to DLBCL 16 months post-diagnosis (tFL), while the other remained non-transformed FL (ntFL). Three tonsil samples from patients with tonsillitis served as controls. All samples were obtained at diagnosis, between February 2015 and September 2017, through surgical excisional lymph-node biopsy. Diagnoses were confirmed by expert hematopathologists following WHO classification criteria. Clinical characteristics are detailed in Table 1. Sample and patient data were provided by the Biobank of the University of Navarra and processed under standard operating procedures approved by the Ethical and Scientific Committees. All patients gave informed consent.

Lymph-node biopsies were divided into pieces, one of which was formalin-fixed and paraffin-embedded and used for diagnosis and for spatial transcriptomic analysis. A second portion of the lymph nodes was mechanically dissociated into a single-cell suspension, then cryopreserved with FBS + 10% DMSO and stored in liquid nitrogen. Upon thawing, cell viability was assessed using acridine orange/propidium iodide fluorescence staining in a Nexcellom Cellometer K2. Samples with less than 80% viable cells were stained with a cell viability dye (namely TO-PRO-3) and sorted to further enrich the sample in live cells. Cells were resuspended in PBS 1X, BSA 0.04% prior to scRNA-seq and/or scDNA-seq analysis.

### 4.2. Single-Cell DNA (scDNA-Seq) and Protein Sequencing

Single-cell DNA and protein sequencing were performed using Mission Bio single cell DNA and protein sequencing V2 kit in a Mission Bio Tapestri platform. For protein analysis, approximately 1 million thawed lymph-node cells were stained with a custom panel of four antibody–oligonucleotide conjugates CD5, CD10, CD19, CD30 (BioLegend Cat# 300647, RRID:AB_2892344; BioLegend Cat# 312239, RRID:AB_2892378; BioLegend Cat# 302277, RRID:AB_2892349; BioLegend Cat# 333923, RRID:AB_2904358; respectively).

About 150,000 single cells were encapsulated using a two-step microfluidic droplet workflow. A custom panel targeting 479 amplicons (175–275 bp) across 42 lymphoma-related genes was designed. Targeted regions were amplified using the Mission Bio Tapestri DNA Reagent Kit according to the manufacturer’s instructions, ensuring precise single-cell mutation capture with cell-specific barcoding. Four DNA-barcoded antibodies were used to target specific cell surface markers (CD5, CD19, CD10, CD30) (Supplementary Tables S2 and S3).

Library quality control and quantification were performed using Qubit 3.0 Fluorometer (Thermo Fisher Scientific; Waltham, MA, USA) and Agilent 4200 TapeStation System (Agilent; Santa Clara, CA, USA), respectively. Final libraries were pooled and sequenced on an Illumina NextSeq2000 (Read 1: 150; Read 2: 150; i7 index: 8; i5 index: 8). Average coverage was 70 reads/amplicons/cell and 750 reads/antibody/cell.

**Data analysis and clonal architecture.** Sequencing data were processed using Mission Bio’s Tapestri Pipeline. Data were analyzed using the Mission Bio portal and Tapestri Insights software (v2.2). Quality control was performed using the Tapestri Insight pipeline to remove low-quality and low-frequency variants. Variants were filtered using the following thresholds: genotype quality score < 30, real depth < 10 reads, single-cell variant allele frequency (VAF) < 25%, variant genotyped in <50% of the cells, cells with <50% of the genotypes present, and variant mutated in <0.5% of the cells. Only variants reported as pathogenic or probably pathogenic in clinical databases (COSMIC, CLINVAR, and Varsome) were retained. Germline variants were discarded.

Clonal architectures were defined by genotype clustering, including zygosity, and visualized using custom R scripts. We included somatic coding (nonsynonymous) variants and cells with complete genotypes. The minimum clone size was 10 cells. Small clones below the ADO (allele dropout) rate (<1%), genotyped as wild type (WT) or homozygous for a given variant but with decreased quality and read depth, were considered false positives. Clonal evolution was visualized in fish plots. We integrated mutation and protein data using Mosaic (v3.4).

**Clonal hematopoiesis (CH) analysis.** Three additional lymph-node samples (1 DLBCL, 2FL) (Table 2) were used for clonal hematopoiesis analysis using scDNA-seq. Samples were mechanically dissociated into single-cell suspensions and sequenced following the above procedure. A commercial myeloid Mission Bio panel was employed, covering hotspot mutations in 46 genes (312 amplicons), including those commonly associated with CH. In addition, 19 DNA-barcoded antibodies were used to target specific cell surface markers (Supplementary Tables S4 and S5) (CD2, CD3, CD7, CD10, CD11b, CD117, CD34, CD38, CD45RA, CD56, CD13, CD123, CD14, HLA-DR, CD19, CD33, CD22, two isotype controls) (BioLegend Cat# 309239, RRID:AB_2894548; BioLegend Cat# 300485, RRID:AB_2892342; BioLegend Cat# 343133, RRID:AB_2892413; BioLegend Cat# 312239, RRID:AB_2892378; BioLegend Cat# 301363, RRID:AB_2892345; BioLegend Cat# 313251, RRID:AB_2904340; BioLegend Cat# 343543, RRID:AB_2892416; BioLegend Cat# 303553, RRID:AB_2892351; BioLegend Cat# 304175, RRID:AB_2892356; BioLegend Cat# 362567, RRID:AB_2904383; BioLegend Cat# 301735, RRID:AB_2892346; BioLegend Cat# 306051, RRID:AB_2892367; BioLegend Cat# 301865, RRID:AB_2892347; BioLegend Cat# 361719, RRID:AB_2922573; BioLegend Cat# 302277, RRID:AB_2892349; BioLegend Cat# 366637, RRID:AB_2894561; BioLegend Cat# 363525, RRID:AB_2894556; respectively).

CH variants were selected if they resulted in a biological loss of function (e.g., missense, nonsense, and frameshift), were not reported as benign or probably benign in clinical databases (CLINVAR, COSMIC, and Varsome), or lacked evidence of benignity from in silico tools.

### 4.3. Single-Cell RNA Sequencing (scRNA-Seq) Analysis

**Library Preparation.** The transcriptome of 12,000 cells was analyzed using Single Cell 3′ Reagent Kits (v3.1, 10x Genomics). To this end, 17,000–20,000 cells were loaded at a concentration of 1000 cells/µL into a Chromium X instrument (10x Genomics) to capture single cells in gel bead-in-emulsions (GEMs). Each cell was encapsulated with primers containing a fixed Illumina Read 1 sequence, a cell-identifying 16-bp 10x barcode, a 12-nt Unique Molecular Identifier (UMI), and a poly-dT sequence. Following cell lysis, reverse transcription yielded full-length, barcoded cDNA. This cDNA was then released from the GEMs, PCR-amplified, and purified with magnetic beads (SPRIselect, Beckman Coulter). Enzymatic fragmentation and size selection were used to optimize cDNA size before library construction. Fragmented cDNA was then end-repaired, A-tailed, and ligated to Illumina adaptors. A final polymerase chain reaction amplification with barcoded primers allowed sample indexing. Library quality control and quantification were performed using a Qubit 3.0 Fluorometer (Thermo Fisher Scientific) and Agilent’s 4200 TapeStation System (Agilent), respectively. Sequencing was performed on an Illumina NextSeq2000 (Read 1: 28; Read 2: 90; i7 index: 10; i5 index: 10), targeting 20,000 reads/cell.

**Single-cell RNA sequencing data processing and analysis.** Raw sequencing reads were processed using Cell Ranger (version 7.0.0, 10x Genomics) and mapped to the GRCh38 human reference genome. Downstream analysis was performed using the Seurat R package (version 4.3.0). During the preprocessing steps, quality control filtering was applied to retain cells expressing between 200 and 6000 genes, and those with a mitochondrial transcript fraction of less than 15%. Furthermore, potential doublets were computationally identified and removed using the DoubletFinder package (version 2.0.3) assuming a doublet formation rate of 10%. The filtered data were normalized using the LogNormalize method. To mitigate the confounding effects of cell cycle heterogeneity, cell cycle scores (S and G2/M phases) were calculated using the CellCycleScoring function, and these scores were regressed out during data scaling. To overcome technical variation across different patient samples and sequencing runs, we performed batch-correction using the Harmony algorithm (version 0.1.1), integrating the datasets across the patient identifier variable. Dimensionality reduction was conducted via Principal Component Analysis (PCA), and the first 30 Harmony-adjusted principal components (PCs) were selected for downstream steps based on Elbow plot evaluation. For clustering, a shared nearest neighbor (SNN) graph was constructed (k-nearest neighbors = 20), followed by Louvain network detection using a resolution parameter of 0.3. Finally, UMAP generation for 2D visualization was computed using the same top 30 adjusted PCs, with standard configuration parameters (n_neighbors = 30, min_dist = 0.3).

**Differential gene expression analysis.** Seurat was used to identify DEGs within T- and B-cell subclusters. Genes were considered differentially expressed if they were detected in >20% of cells in at least one cluster, with a log fold change >0.2 and a Bonferroni-adjusted *p*-value < 0.05 (Wilcoxon rank-sum test). Given the study design, these analyses were performed at the single-cell level within individual samples and are intended for exploratory and descriptive purposes rather than formal statistical inference between disease groups.

**Gene set variation analysis (GSVA).** GSVA was performed using the scGSVA R package. Gene-cell matrices were converted to gene set-cell matrices, and GSVA scores were computed for gene sets with ≥5 detected genes. All other parameters were set to default. GSVA analyses were conducted at the single-cell level within individual samples and are interpreted descriptively.

**Exhaustion/immunosuppressive and adhesion signature scores.** Exhaustion/immunosuppressive and adhesion transcriptional signatures were computed using the AddModuleScore function in Seurat (v4.3.0), based on differentially expressed genes identified in our cohort. The exhaustion/immunosuppresive signature included *PDCD1*, *LAG3*, and *HAVCR2* and was evaluated in CD8^+^ effector and CD4^+^ regulatory T cells. The adhesion signature included *CD2*, *CTLA4*, *MAF*, *PDCD1*, *CD40LG*, and *ICOS*, and was evaluated in CD4^+^ follicular helper T cells.

**Validation in an independent single-cell cohort.** To validate our findings, we applied the exhaustion/immunosuppressive and adhesion transcriptional signatures to an independent single-cell RNA-sequencing dataset of follicular patients published by Sarkozy et al. This dataset comprises 11 FL patients with pre-/post-transformation paired biopsies, alongside an additional 11 FL patients who had no disease progression within six years. For validation, we included 9 pre-transformation FL biopsies (excluding tFL4 and tFL5, who had received treatment prior to biopsy) and 11 non-progressor FL biopsies. Patients were stratified into short-term (stFL) and long-term (ltFL) transformed FL based on time to transformation, defined as ≤12 months and >12 months from diagnosis, respectively. Gene expression matrices were downloaded from the European Genome-phenome Archive (EGAS00001007023) and imported into Seurat (v4.3.0). Cells were filtered and normalized using SCTransform. Cell types were annotated based on our single-cell reference dataset. Signature scores were calculated at the single-cell level and then aggregated per patient for comparative analysis between transformed and non-transformed groups.

### 4.4. Spatial Transcriptomics Analysis

**Spatial library preparation and sequencing.** Formalin-fixed paraffin-embedded (FFPE) lymph-node blocks were kindly provided by the Anatomical Pathology Department at the Clínica Universidad de Navarra. We included FFPE tissue blocks from ntFL, GCB DLBCL, and tFL at diagnosis (tFL-FL) and transformation (tFL-DLBCL). These samples were selected based on our preceding scRNA-seq and scDNA-seq analyses, which identified the most pronounced biological differences along the follicular transformation axis, particularly between the ntFL and tFL, with GCB DLBCL representing the closest transcriptional counterpart of tFL. ABC DLBCL samples were therefore not included in this analysis.

Prior to spatial transcriptomics experiments, RNA integrity was examined on 10 µm sections. RNA was extracted using the RNeasy FFPE Kit (Qiagen; Hilden, Germany) and evaluated with RNA ScreenTape Assay. Samples with DV200 > 30% were further sectioned. Five µm tissue sections were mounted onto microscopy slides, baked at 42 °C for 3 h, and dried overnight at 37 °C. Slides were deparaffinized, rehydrated, and H&E stained. Tissue imaging was performed using an Aperio CS2 Scanner (Leica Biosystems; Nussloch, Germany) at 40x magnification. Sections were destained and decrosslinked before library construction using the Visium HD Spatial Gene Expression Reagent Kit for FFPE Human Transcriptome (10x Genomics). Briefly, a whole transcriptome panel consisting of three probe pairs per gene was hybridized with target RNAs in the tissue sections, followed by ligation. Tissue slides and a Visium HD Slide were then loaded into a Visium CytAssist instrument (10x Genomics). Ligated probes were released and diffused onto Visium HD slides containing spatially barcoded oligonucleotides. Probes were thus spatially labeled through extension, then released from the slide, pooled, and PCR-indexed. Libraries were quantified with Qubit dsDNA HS Assay Kit and assessed with Agilent’s HS D1000 ScreenTape Assay. Sequencing was performed on an Illumina NextSeq2000 (Read 1: 43; Read 2: 50; i7 index: 10; i5 index: 10) at a minimum depth of 275 million reads per capture area.

**Spatial transcriptomics analysis.** Space Ranger platform (10x Genomics) was used to identify the tissue capture areas on the slide and assign reads to each bin based on spatial barcode information (8 × 8 μm and 16 × 16 μm). Quality control filters were applied based on the total number of bins, the number of read pairs in each bin, gene count, and UMI count. Counts were log-normalized and scaled.

We calculated standardized expression levels of each of 9 relevant cell-type gene signatures, derived from the paired scRNA-seq dataset, in each 8 × 8 μm bin using the AddModuleScore function from the Seurat package. Each bin was assigned to the cell type with the highest score. When multiple cell types shared the top score, bins were annotated with a combined label (<0.1%). Bins with negative scores across all cell types were excluded from downstream analysis (2.2–9.1%). Spatial data were visualized using Loupe Browser (v8.1, 10x Genomics).

Bin-to-bin neighborhood enrichment and ligand–receptor interaction analyses were performed using the SquidPY [55] framework, with parameter adjustments for Visium HD data. Given the continuous 8 × 8 μm grid in Visium HD, no spatial gap was assumed between adjacent bins. Neighborhood enrichment results were normalized and scaled to a 0–1 range across all samples to enable comparative analyses.

### 4.5. Quantification and Statistical Analysis

All statistical analyses were performed in R (v4.2.1). The *p*-values and adjusted *p*-values for the identification of differentially expressed genes in Figure 3 and Figure 4 were calculated using the two-sided Wilcoxon rank-sum test with Bonferroni correction for multiple comparisons. The *p*-values for the percentages of cells per sample were calculated using Fisher’s exact test.

Given the limited sample size, all statistical analyses were performed at the single-cell level within individual samples. Resulting *p*-values reflect within-sample variability and are descriptive only.

## 5. Conclusions

In summary, this study demonstrates the feasibility and translational potential of integrating scDNA-seq, scRNA-seq, and spatial transcriptomics in diagnostic GC lymphoma biopsies. Our findings suggest that immune microenvironmental remodeling may be associated with FL transformation. We describe an immunoregulatory niche observed in samples with early FL transformation, characterized by immunosuppressive CD4^+^ Tregs and exhausted CD8^+^ T cells, together with enhanced Tfh-B-cell interactions. This work provides a framework for understanding the organization of GC lymphomas and illustrates how the integration of single-cell and spatial analyses can generate biological insights, with potential clinical applications including biomarker development and rational design of immunotherapies.

## Figures and Tables

**Table 1:** Clinical characteristics of five lymphoma patients.

Sample ID	Diagnosis	Subtype	Biopsy	Gender	Age at Diagnosis (Years)	Stage at Diagnosis	Histological Transformation	Time from Diagnosis to Transformation (Months)
ABC1-DLBCL	DLBCL	ABC	Diagnostic	Female	73	IV	Not applicable	-
ABC2-DLBCL	DLBCL	ABC	Diagnostic	Female	63	IV	Not applicable	-
GCB-DLBCL	DLBCL	GCB	Diagnostic	Female	48	II	Not applicable	-
tFL	FL	Grade 2	Diagnostic	Male	48	IV	Yes	-
ntFL	FL	Grade 2	Diagnostic	Male	72	IV	No	16

**Table 2:** Clinical characteristics of three lymphoma patients included in the scDNA-seq CH analysis.

Sample ID	Diagnosis	Subtype	Biopsy	Gender	Age at Diagnosis (Years)	Stage at Diagnosis
CH1	FL	Grade 2	Diagnostic	Female	49	IV
CH2	FL	Grade 2	Diagnostic	Female	51	IV
CH3	DLBCL	GCB	Diagnostic	Male	84	II

## Data Availability

The data presented in this study are available on request from the corresponding author due to privacy restrictions related to patient genomic data.

## Supplementary Materials

The following supporting information can be downloaded at: https://www.mdpi.com/article/10.3390/cancers18071122/s1, Figure S1: Identification of malignant and non-malignant B-cell populations in integrated scRNA-seq data; Table S1: Clinical characteristics of non-malignant tonsillar control samples; Table S2: Custom GC-lymphoma panel covering 42 genes (479 amplicons; 175–275 bp) and 4 DNA-barcoded antibodies; Table S3: Amplicon coverage of genes included in the custom lymphoma scDNA-seq panel; Table S4: Commercial myeloid Mission Bio panel covering hotspot mutations in 46 genes (312 amplicons) and 19 DNA-barcoded antibodies; Table S5: Amplicon coverage of genes included in the myeloid-targeted scDNA-seq panel; Table S6: Differential expression analysis between malignant B-cell subclusters.
