# Supplementary material for: Integrated Single-Cell and Spatial Multi-Omics of Clonal Precursors and Immune Niches in Germinal Center Lymphomas
Source: Cancers (Basel). 2026 Mar 31;18(7):1122. doi: 10.3390/cancers18071122 (PMC13072214; doi:10.3390/cancers18071122)
Supplement: Supplementary file 1 [file cancers-18-01122-s001.zip › cancers-4212998-supplementary.pdf]

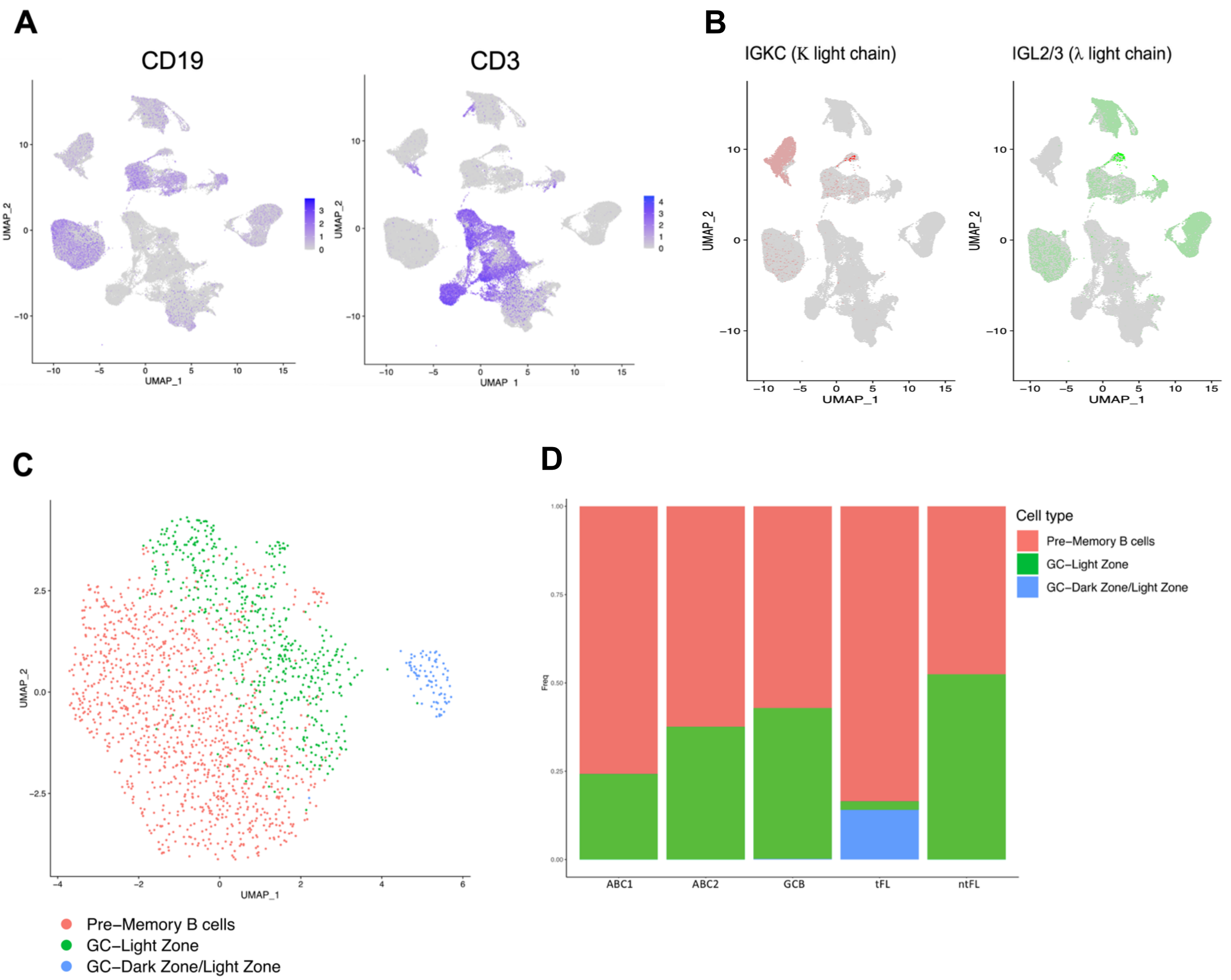

Supplementary Figure 1. **Identification of malignant and non-malignant B-cell populations in integrated scRNA-seq data (A)** UMAP of merged scRNA-seq data from five lymphoma samples (ABC1, ABC2, GCB, tFL, and ntFL) and three reactive tonsil controls showing the expression of CD19 and CD3. **(B)** Clonality of malignant B cells was independently confirmed by immunoglobulin light-chain restriction, assessed by the expression of IGKC (κ light chain) and IGLC2/3 (λ light chain). Malignant B-cell clusters exhibited strongly skewed light-chain expression (<1% or >91% IGKC (Igκ) / IGLC2/3 (Igλ)), consistent with clonal expansion. **(C)** UMAP of non-malignant B cells derived from the five lymphoma samples (ABC1, ABC2, GCB, tFL, ntFL). Three distinct non-malignant B-cell subclusters were identified: pre-memory B cell cluster, germinal-center light zone (GC-LZ) cluster and intermediate GC cluster. **(D)** Bar plot representing the relative contribution of non-malignant B-cell subclusters to each patient samples (ABC1, ABC2, GCB, tFL, and ntFL). Colors denote the three non-malignant B-cell subclusters defined in (C).

Supplementary Table 1. Clinical characteristics of non-malignant tonsillar control samples.

Sample	Sex	Age (years)	Diagnosis
Tonsil 1	Male	17	Chronic tonsillitis
Tonsil 2	Female	4	Chronic tonsillitis
Tonsil 3	Female	4	Chronic tonsillitis

*Tonsillar samples were included to assist in the identification of non-malignant cell populations and to distinguish malignant from non-malignant B cells in the scRNA-seq analysis. These samples represent an activated immune state and were not used for quantitative or differential comparisons with lymphoma samples.

Supplementary Table 2. Custom GC-lymphoma panel covering 42 genes (479 amplicons; 175-275 bp) and 4 DNA-barcoded antibodies.

42-gene lymphoma panel					
ARID1A	ARID1B	B2M	ATM	BCL2	BRAF
BTG	CARD11	CCND3	CD79A	CD79B	CDKN2A
CREBBP	CTSS	EP300	EZH2	FOXO1	GNA13
HIST1H1B	HIST1H1C	HIST1H1D	HIST1H1E	ID3	KMT2D
KRAS	MAP2K1	MYC	MYD88	NOTCH1	NOTCH2
PIK3CD	PIM1	PTEN	RHOA	SF3B1	SOCS1
STAT3	STAT6	TET2	TNFAIP3	TNFRSF14	TP53

4 DNA-barcoded antibodies			
CD19	CD10	CD5	CD30

Supplementary Table 3. Amplicon coverage of genes included in the custom lymphoma scDNA-seq panel.

target	chr	start	end	bases_covered	bases_missed	coverage_perc
TNFRSF14:p.M1L	chr1	2488104	2488104	1	0	100.0
TNFRSF14:p.M1V	chr1	2488104	2488104	1	0	100.0
TNFRSF14:p.M1I	chr1	2488106	2488106	1	0	100.0
TNFRSF14:p.W7*	chr1	2488123	2488123	1	0	100.0
TNFRSF14:p.W7*	chr1	2488124	2488124	1	0	100.0
TNFRSF14:p.P9T	chr1	2488128	2488128	1	0	100.0
TNFRSF14:p.W12*	chr1	2488138	2488138	1	0	100.0
TNFRSF14:p.W12*	chr1	2488139	2488139	1	0	100.0
TNFRSF14:p.S14C	chr1	2488144	2488144	1	0	100.0
TNFRSF14:c.43_47delACCCC	chr1	2488144	2488148	5	0	100.0
TNFRSF14:c.41_45delCCACC	chr1	2488144	2488148	5	0	100.0
TNFRSF14:c.43_44insC	chr1	2488147	2488147	1	0	100.0
TNFRSF14:p.D19H	chr1	2488158	2488158	1	0	100.0
TNFRSF14:c.69delG	chr1	2488172	2488172	1	0	100.0
TNFRSF14:c.69+2T>C	chr1	2488174	2488174	1	0	100.0
TNFRSF14:p.G60D	chr1	2489782	2489782	1	0	100.0
TNFRSF14:p.Y61C	chr1	2489785	2489785	1	0	100.0
TNFRSF14:p.E65*	chr1	2489796	2489796	1	0	100.0
TNFRSF14:p.G72D	chr1	2489818	2489818	1	0	100.0
TNFRSF14:c.225_226delTG	chr1	2489823	2489824	2	0	100.0
TNFRSF14:p.C78R	chr1	2489835	2489835	1	0	100.0
TNFRSF14:p.C78R	chr1	2489835	2489837	3	0	100.0
TNFRSF14:p.T82P	chr1	2489847	2489847	1	0	100.0
TNFRSF14:p.T82I	chr1	2489848	2489848	1	0	100.0
TNFRSF14:p.Y83D	chr1	2489850	2489850	1	0	100.0
TNFRSF14:p.S91C	chr1	2489874	2489874	1	0	100.0
TNFRSF14:p.C96R	chr1	2489889	2489889	1	0	100.0
TNFRSF14:p.C96R	chr1	2489889	2489891	3	0	100.0
TNFRSF14:p.Q97*	chr1	2489892	2489892	1	0	100.0
TNFRSF14:p.Q158*	chr1	2492074	2492074	1	0	100.0
TNFRSF14:p.C162*	chr1	2492087	2492088	2	0	100.0
TNFRSF14:p.C162*	chr1	2492088	2492088	1	0	100.0

TNFRSF14:p.Q180*	chr1	2492140	2492140	1	0	100.0
TNFRSF14:c.576_585del	chr1	2493136	2493145	10	0	100.0
TNFRSF14:p.W201*	chr1	2493162	2493162	1	0	100.0
TNFRSF14:p.W201*	chr1	2493163	2493163	1	0	100.0
TNFRSF14:p.W203*	chr1	2493168	2493168	1	0	100.0
TNFRSF14:c.608_612delGGTGG	chr1	2493168	2493172	5	0	100.0
TNFRSF14:p.W203*	chr1	2493169	2493169	1	0	100.0
TNFRSF14:p.W204*	chr1	2493171	2493171	1	0	100.0
TNFRSF14:p.W204*	chr1	2493172	2493172	1	0	100.0
TNFRSF14:p.V215D	chr1	2493204	2493204	1	0	100.0
TNFRSF14:p.G232S	chr1	2493254	2493254	1	0	100.0
TNFRSF14:p.G232S	chr1	2494304	2494304	1	0	100.0
TNFRSF14:p.V267M	chr1	2494659	2494659	1	0	100.0
PIK3CD:p.E1045K	chr1	9787030	9787030	1	0	100.0
ID3	chr1	23885450	23885510	61	0	100.0
ID3	chr1	23885617	23885917	263	38	87.37541528239203
ARID1A	chr1	27022894	27024031	59	1079	5.184534270650263
ARID1A	chr1	27056141	27056354	201	13	93.92523364485982
ARID1A	chr1	27057642	27058095	399	55	87.88546255506608
ARID1A:p.Q479*	chr1	27057727	27057727	1	0	100.0
ARID1A	chr1	27059166	27059283	118	0	100.0
ARID1A	chr1	27087346	27087587	208	34	85.9504132231405
ARID1A:p.G721R	chr1	27087587	27087587	1	0	100.0
ARID1A	chr1	27087874	27087964	91	0	100.0
ARID1A:p.G721R	chr1	27087876	27087876	1	0	100.0
ARID1A	chr1	27088642	27088810	169	0	100.0
ARID1A	chr1	27089463	27089776	275	39	87.57961783439491
ARID1A	chr1	27092711	27092857	147	0	100.0
ARID1A	chr1	27092947	27093057	111	0	100.0
ARID1A	chr1	27094280	27094490	197	14	93.36492890995261
ARID1A	chr1	27097609	27097817	207	2	99.04306220095694
ARID1A	chr1	27098990	27099123	134	0	100.0
ARID1A	chr1	27099302	27099478	177	0	100.0
ARID1A	chr1	27099836	27099987	152	0	100.0
ARID1A:p.R1276*	chr1	27099947	27099947	1	0	100.0
ARID1A	chr1	27100070	27100208	139	0	100.0
ARID1A	chr1	27100292	27100389	98	0	100.0
ARID1A	chr1	27100819	27101711	757	136	84.77043673012318
ARID1A:c.4834_4835delAA	chr1	27101552	27101553	2	0	100.0
ARID1A	chr1	27102067	27102198	132	0	100.0
ARID1A	chr1	27105513	27107247	1462	273	84.26512968299711
chr1:120457895-120459335	chr1	120457895	120459335	1206	235	83.69188063844553
NOTCH2:c.6908_6909delCC	chr1	120458436	120458437	2	0	100.0
NOTCH2:c.6880_6887delATAACCAC	chr1	120458458	120458465	8	0	100.0
NOTCH2:c.6876_6877insAG	chr1	120458469	120458469	1	0	100.0
NOTCH2:p.E2206*	chr1	120458729	120458729	1	0	100.0
NOTCH2:c.6404_6405delTG	chr1	120458940	120458941	2	0	100.0
NOTCH2:p.C284*	chr1	120529605	120529605	1	0	100.0
NOTCH2:p.C284*	chr1	120529605	120529606	2	0	100.0
CTSS	chr1	150705521	150705621	101	0	100.0
CTSS	chr1	150720252	150720355	104	0	100.0
CTSS	chr1	150722481	150722647	146	21	87.42514970059881
CTSS	chr1	150724256	150724484	209	20	91.26637554585153
CTSS	chr1	150727476	150727626	151	0	100.0
CTSS	chr1	150730333	150730456	124	0	100.0
CTSS	chr1	150737113	150737239	127	0	100.0
chr2:198266200-198266260	chr2	198266200	198266260	61	0	100.0
chr2:198266460-198266620	chr2	198266460	198266620	161	0	100.0
chr2:198266700-198266860	chr2	198266700	198266860	159	2	98.75776397515529
chr2:198267350-198267560	chr2	198267350	198267560	201	10	95.260663507109
chr2:198267670-198267760	chr2	198267670	198267760	91	0	100.0
chr2:198268300-198268500	chr2	198268300	198268500	195	6	97.01492537313433
MYD88:p.S219C	chr3	38182032	38182032	1	0	100.0
MYD88:p.S243N	chr3	38182292	38182292	1	0	100.0
chr3:38182605-38182785	chr3	38182605	38182785	174	7	96.13259668508287
MYD88:p.L265P	chr3	38182641	38182641	1	0	100.0
RHOA	chr3	49397641	49397815	175	0	100.0
RHOA	chr3	49398360	49398499	0	140	0.0
RHOA	chr3	49399928	49400059	132	0	100.0
RHOA	chr3	49405860	49405981	122	0	100.0
RHOA	chr3	49412866	49413022	157	0	100.0
chr4:106068032-106068136	chr4	106068032	106068136	105	0	100.0
TET2:c.1342delG	chr4	106156441	106156441	1	0	100.0
TET2:p.Q743*	chr4	106157326	106157326	1	0	100.0
TET2:p.P874A	chr4	106157719	106157719	1	0	100.0
TET2:p.Q886*	chr4	106157755	106157755	1	0	100.0
TET2:c.2741_2746delTTGCTCinsG	chr4	106157840	106157845	6	0	100.0
TET2:c.2752_2753delAG	chr4	106157851	106157852	2	0	100.0
TET2:c.3954+2T>G	chr4	106180928	106180928	1	0	100.0
TET2:p.P1419R	chr4	106193794	106193794	1	0	100.0
chr4:106196204-106197700	chr4	106196204	106197700	1252	245	83.63393453573815
TET2:p.R1516*	chr4	106196213	106196213	1	0	100.0
TET2:p.I1762R	chr4	106196952	106196952	1	0	100.0
HIST1H1C	chr6	26055967	26056656	591	99	85.65217391304348

HIST1H1E	chr6	26156558	26157278	618	103	85.71428571428571
HIST1H1D	chr6	26234439	26235161	610	113	84.37067773167358
HIST1H1B	chr6	27834569	27835307	629	110	85.11502029769959
PIM1:p.Q127*	chr6	37139039	37139039	1	0	100.0
PIM1:c.402delG	chr6	37139062	37139062	1	0	100.0
PIM1:p.E135K	chr6	37139063	37139063	1	0	100.0
PIM1:p.S146N	chr6	37139097	37139097	1	0	100.0
PIM1:p.W149C	chr6	37139107	37139107	1	0	100.0
PIM1:p.L182F	chr6	37139204	37139204	1	0	100.0
chr6:41903665-41903850	chr6	41903665	41903850	186	0	100.0
chr6:44232715-44233080	chr6	44232715	44233080	278	88	75.95628415300547
chr6:138188395-138188631	chr6	138188395	138188631	203	34	85.65400843881856
chr6:138192350-138192659	chr6	138192350	138192659	269	41	86.7741935483871
TNFAIP3:c.211_212delAG	chr6	138192575	138192576	2	0	100.0
TNFAIP3:c.262_263insA	chr6	138192627	138192627	1	0	100.0
TNFAIP3:c.291_295+4delGAACGGTAA	chr6	138192655	138192663	9	0	100.0
TNFAIP3:c.295+2T>G	chr6	138192661	138192661	1	0	100.0
TNFAIP3:c.301delG	chr6	138195987	138195987	1	0	100.0
TNFAIP3:c.397_398insA	chr6	138196084	138196084	1	0	100.0
chr6:138196824-138196972	chr6	138196824	138196972	149	0	100.0
TNFAIP3:c.533_534delAC	chr6	138196871	138196872	2	0	100.0
chr6:138197132-138197303	chr6	138197132	138197303	172	0	100.0
chr6:138198212-138198393	chr6	138198212	138198393	173	9	95.05494505494505
chr6:138199568-138200488	chr6	138199568	138200488	784	137	85.12486427795874
TNFAIP3:p.R439L	chr6	138199898	138199898	1	0	100.0
TNFAIP3:c.1363delG	chr6	138199945	138199945	1	0	100.0
chr6:138201207-138201389	chr6	138201207	138201389	183	0	100.0
chr6:138202170-138202480	chr6	138202170	138202480	272	39	87.45980707395498
TNFAIP3:c.2109_2110delTG	chr6	138202192	138202193	2	0	100.0
TNFAIP3:c.2290delG	chr6	138202373	138202373	1	0	100.0
ARID1B	chr6	157099063	157100605	396	1147	25.664290343486716
ARID1B	chr6	157150360	157150555	176	20	89.79591836734694
ARID1B	chr6	157192747	157192786	40	0	100.0
ARID1B	chr6	157222509	157222659	151	0	100.0
ARID1B	chr6	157256599	157256710	112	0	100.0
ARID1B	chr6	157405795	157406039	209	36	85.3061224489796
ARID1B	chr6	157431605	157431695	91	0	100.0
ARID1B	chr6	157454161	157454341	181	0	100.0
ARID1B	chr6	157469757	157470085	291	38	88.44984802431611
ARID1B	chr6	157488173	157488319	147	0	100.0
ARID1B	chr6	157495141	157495251	111	0	100.0
ARID1B	chr6	157495980	157496139	157	3	98.125
ARID1B	chr6	157502102	157502312	207	4	98.10426540284361
ARID1B	chr6	157505364	157505569	198	8	96.11650485436893
ARID1B	chr6	157510775	157510914	140	0	100.0
ARID1B	chr6	157511171	157511344	174	0	100.0
ARID1B	chr6	157517298	157517449	152	0	100.0
ARID1B	chr6	157519944	157520041	98	0	100.0
ARID1B	chr6	157521838	157522622	668	117	85.09554140127389
ARID1B	chr6	157524999	157525130	132	0	100.0
ARID1B	chr6	157527300	157529025	1451	275	84.06720741599074
CARD11	chr7	2946271	2946476	171	35	83.00970873786407
CARD11	chr7	2949683	2949799	117	0	100.0
CARD11	chr7	2951805	2951930	126	0	100.0
CARD11	chr7	2952920	2953100	168	13	92.81767955801105
CARD11	chr7	2954870	2955006	137	0	100.0
CARD11	chr7	2956923	2957019	97	0	100.0
CARD11	chr7	2958124	2958221	98	0	100.0
CARD11	chr7	2959005	2959246	213	29	88.01652892561984
CARD11:p.L251P	chr7	2959177	2959177	1	0	100.0
CARD11	chr7	2962267	2962394	128	0	100.0
CARD11	chr7	2962765	2962967	191	12	94.08866995073892
CARD11	chr7	2963866	2963999	121	13	90.29850746268657
CARD11	chr7	2966372	2966425	54	0	100.0
CARD11	chr7	2968231	2968332	102	0	100.0
CARD11	chr7	2969625	2969708	84	0	100.0
CARD11	chr7	2972168	2972220	53	0	100.0
CARD11	chr7	2974086	2974263	170	8	95.50561797752809
CARD11	chr7	2976670	2976868	178	21	89.44723618090453
CARD11	chr7	2977540	2977666	127	0	100.0
CARD11:p.R379W	chr7	2977549	2977549	1	0	100.0
CARD11:p.M360V	chr7	2977606	2977606	1	0	100.0
CARD11:p.D357G	chr7	2977614	2977614	1	0	100.0
CARD11:c.1030_1032delAAG	chr7	2977652	2977654	3	0	100.0
CARD11	chr7	2978312	2978465	148	6	96.1038961038961
CARD11:p.R309H	chr7	2978404	2978404	1	0	100.0
CARD11	chr7	2979382	2979562	171	10	94.47513812154696
CARD11:p.V266G	chr7	2979450	2979450	1	0	100.0
CARD11:p.L251P	chr7	2979495	2979495	1	0	100.0
CARD11:p.S250P	chr7	2979499	2979499	1	0	100.0
CARD11:p.Q249P	chr7	2979501	2979501	1	0	100.0
CARD11:p.K238T	chr7	2979534	2979534	1	0	100.0
CARD11:p.D230N	chr7	2979559	2979559	1	0	100.0
CARD11	chr7	2983845	2984171	289	38	88.37920489296636

CARD11:p.R149H	chr7	2984084	2984084	1	0	100.0
CARD11:p.F130V	chr7	2984142	2984142	1	0	100.0
CARD11:p.T128M	chr7	2984147	2984147	1	0	100.0
CARD11:p.G126E	chr7	2984152	2984153	2	0	100.0
CARD11:p.G123D	chr7	2984162	2984162	1	0	100.0
CARD11:p.G123S	chr7	2984162	2984163	2	0	100.0
CARD11:p.G123S	chr7	2984163	2984163	1	0	100.0
CARD11	chr7	2985452	2985590	139	0	100.0
CARD11:p.T117A	chr7	2985462	2985462	1	0	100.0
CARD11:p.S116P	chr7	2985465	2985465	1	0	100.0
CARD11:p.F115I	chr7	2985468	2985468	1	0	100.0
CARD11:p.E93Q	chr7	2985534	2985534	1	0	100.0
CARD11	chr7	2987208	2987421	205	9	95.79439252336448
CARD11	chr7	2998133	2998140	8	0	100.0
chr7:140453070-140453200	chr7	140453070	140453200	131	0	100.0
BRAF:p.V600E	chr7	140453136	140453136	1	0	100.0
BRAF:p.D594G	chr7	140453154	140453154	1	0	100.0
BRAF:p.G469A	chr7	140477800	140477800	1	0	100.0
BRAF:p.G469V	chr7	140477800	140477800	1	0	100.0
chr7:140481370-140481500	chr7	140481370	140481500	131	0	100.0
BRAF:p.G469A	chr7	140481402	140481402	1	0	100.0
BRAF:p.G469V	chr7	140481402	140481402	1	0	100.0
BRAF:p.G469A	chr7	140481411	140481411	1	0	100.0
BRAF:p.G469V	chr7	140481411	140481411	1	0	100.0
EZH2	chr7	148504737	148504798	62	0	100.0
EZH2:p.A682G	chr7	148504781	148504781	1	0	100.0
EZH2	chr7	148506162	148506247	86	0	100.0
EZH2:p.M706V	chr7	148506242	148506242	1	0	100.0
EZH2	chr7	148506401	148506482	82	0	100.0
EZH2:p.A682G	chr7	148506467	148506467	1	0	100.0
EZH2	chr7	148507424	148507506	83	0	100.0
EZH2	chr7	148508716	148508812	97	0	100.0
EZH2:p.Y646C	chr7	148508727	148508727	1	0	100.0
EZH2:p.Y646F	chr7	148508727	148508727	1	0	100.0
EZH2:p.Y646S	chr7	148508727	148508727	1	0	100.0
EZH2:p.Y646S	chr7	148508727	148508728	2	0	100.0
EZH2:p.Y646H	chr7	148508728	148508728	1	0	100.0
EZH2:p.Y646N	chr7	148508728	148508728	1	0	100.0
EZH2:p.E645D	chr7	148508729	148508729	1	0	100.0
EZH2:p.N640K	chr7	148508744	148508744	1	0	100.0
EZH2	chr7	148511050	148511229	180	0	100.0
EZH2	chr7	148512005	148512131	127	0	100.0
EZH2	chr7	148512597	148512638	42	0	100.0
EZH2	chr7	148513775	148513870	96	0	100.0
EZH2	chr7	148514313	148514483	171	0	100.0
EZH2	chr7	148514968	148515209	211	31	87.1900826446281
EZH2	chr7	148516687	148516779	93	0	100.0
EZH2	chr7	148523545	148523724	167	13	92.77777777777777
EZH2	chr7	148524255	148524358	104	0	100.0
EZH2	chr7	148525831	148525972	142	0	100.0
EZH2:p.D183E	chr7	148525881	148525881	1	0	100.0
EZH2:p.D183E	chr7	148525908	148525908	1	0	100.0
EZH2	chr7	148526819	148526940	122	0	100.0
EZH2	chr7	148529725	148529842	118	0	100.0
EZH2	chr7	148543561	148543690	130	0	100.0
EZH2	chr7	148544273	148544390	118	0	100.0
chr8:128748800-128748870	chr8	128748800	128748870	71	0	100.0
MYC:p.N9K	chr8	128748866	128748866	1	0	100.0
chr8:128750496-128751265	chr8	128750496	128751265	654	116	84.93506493506493
MYC:p.N9K	chr8	128750535	128750535	1	0	100.0
MYC:p.E39A	chr8	128750624	128750624	1	0	100.0
MYC:p.L40M	chr8	128750626	128750626	1	0	100.0
MYC:p.P57S	chr8	128750632	128750632	1	0	100.0
MYC:p.P57S	chr8	128750632	128750633	2	0	100.0
MYC:p.P57S	chr8	128750635	128750635	1	0	100.0
MYC:p.P57S	chr8	128750635	128750637	3	0	100.0
MYC:p.P57S	chr8	128750677	128750677	0	1	0.0
MYC:p.P57S	chr8	128750677	128750678	0	2	0.0
MYC:p.T58A	chr8	128750680	128750680	1	0	100.0
MYC:p.T58I	chr8	128750681	128750681	1	0	100.0
MYC:p.T95A	chr8	128750791	128750791	1	0	100.0
MYC:p.S203N	chr8	128751116	128751116	1	0	100.0
MYC:p.E257Q	chr8	128751232	128751232	1	0	100.0
MYC:p.E257Q	chr8	128751235	128751235	1	0	100.0
chr8:128752630-128753220	chr8	128752630	128753220	514	77	86.97123519458545
MYC:p.H307Y	chr8	128752758	128752758	1	0	100.0
CDKN2A	chr9	21968227	21968241	15	0	100.0
CDKN2A	chr9	21968723	21968770	48	0	100.0
CDKN2A	chr9	21970900	21971207	70	238	22.727272727272727
CDKN2A	chr9	21974475	21974826	276	76	78.4090909090909
CDKN2A	chr9	21994137	21994330	184	10	94.84536082474227
chr9:139390500-139392050	chr9	139390500	139392050	1205	346	77.69181173436493
chr9:139397620-139397800	chr9	139397620	139397800	164	17	90.60773480662984
chr9:139399100-139399600	chr9	139399100	139399600	388	113	77.44510978043913

NOTCH1:p.D259N	chr9	139413985	139413985	1	0	100.0
PTEN:c.6_27del	chr10	89623712	89623733	22	0	100.0
chr10:89624200-89624305	chr10	89624200	89624305	106	0	100.0
PTEN:c.6_27del	chr10	89624235	89624256	22	0	100.0
PTEN:p.S10N	chr10	89624255	89624255	1	0	100.0
chr10:89653781-89653866	chr10	89653781	89653866	86	0	100.0
chr10:89685269-89685314	chr10	89685269	89685314	46	0	100.0
chr10:89690802-89690846	chr10	89690802	89690846	45	0	100.0
chr10:89692769-89693008	chr10	89692769	89693008	204	36	85.0
chr10:89711874-89712016	chr10	89711874	89712016	143	0	100.0
PTEN:p.R173H	chr10	89711900	89711900	1	0	100.0
PTEN:c.6_27del	chr10	89711979	89712000	22	0	100.0
PTEN:p.S10N	chr10	89712002	89712002	1	0	100.0
chr10:89717609-89717776	chr10	89717609	89717776	168	0	100.0
PTEN:p.R233*	chr10	89717672	89717672	1	0	100.0
PTEN:p.K342N	chr10	89720875	89720875	1	0	100.0
chr10:89725000-89725250	chr10	89725000	89725250	210	41	83.66533864541833
chr11:108093793-108093913	chr11	108093793	108093913	121	0	100.0
chr11:108098330-108098430	chr11	108098330	108098430	83	18	82.17821782178218
chr11:108098502-108098615	chr11	108098502	108098615	114	0	100.0
chr11:108099904-108100050	chr11	108099904	108100050	136	11	92.51700680272108
chr11:108106396-108106561	chr11	108106396	108106561	162	4	97.59036144578313
chr11:108114650-108114850	chr11	108114650	108114850	158	43	78.60696517412936
chr11:108115514-108115753	chr11	108115514	108115753	207	33	86.25
chr11:108117690-108117854	chr11	108117690	108117854	165	0	100.0
chr11:108119659-108119829	chr11	108119659	108119829	147	24	85.96491228070175
ATM:p.Q466*	chr11	108121588	108121588	1	0	100.0
chr11:108122563-108122758	chr11	108122563	108122758	190	6	96.93877551020408
chr11:108123543-108123639	chr11	108123543	108123639	97	0	100.0
ATM:p.P604S	chr11	108123551	108123551	1	0	100.0
ATM:p.P604S	chr11	108123551	108123552	2	0	100.0
chr11:108124540-108124766	chr11	108124540	108124766	196	31	86.34361233480176
chr11:108126941-108127067	chr11	108126941	108127067	115	12	90.55118110236221
chr11:108128207-108128333	chr11	108128207	108128333	127	0	100.0
chr11:108129712-108129802	chr11	108129712	108129802	91	0	100.0
chr11:108137897-108138069	chr11	108137897	108138069	173	0	100.0
ATM:p.F858L	chr11	108138003	108138003	1	0	100.0
ATM:p.F858L	chr11	108138005	108138005	1	0	100.0
chr11:108139136-108139336	chr11	108139136	108139336	183	18	91.04477611940298
chr11:108141790-108141873	chr11	108141790	108141873	83	1	98.80952380952381
chr11:108141977-108142133	chr11	108141977	108142133	157	0	100.0
ATM:p.H996Q	chr11	108142044	108142044	1	0	100.0
chr11:108143258-108143334	chr11	108143258	108143334	77	0	100.0
ATM:p.M1040V	chr11	108143299	108143299	1	0	100.0
chr11:108143448-108143579	chr11	108143448	108143579	132	0	100.0
chr11:108150217-108150335	chr11	108150217	108150335	119	0	100.0
chr11:108151721-108151895	chr11	108151721	108151895	160	15	91.42857142857143
chr11:108153436-108153606	chr11	108153436	108153606	170	1	99.41520467836257
chr11:108154953-108155200	chr11	108154953	108155200	204	44	82.25806451612904
chr11:108158326-108158442	chr11	108158326	108158442	117	0	100.0
chr11:108159703-108159830	chr11	108159703	108159830	128	0	100.0
ATM:p.H1380Y	chr11	108159732	108159732	1	0	100.0
ATM:p.I1407S	chr11	108159813	108159814	2	0	100.0
ATM:p.I1407S	chr11	108159814	108159814	1	0	100.0
chr11:108160328-108160528	chr11	108160328	108160528	181	20	90.04975124378109
ATM:p.K1454N	chr11	108160454	108160454	1	0	100.0
ATM:p.F1463S	chr11	108160479	108160480	2	0	100.0
ATM:p.F1463S	chr11	108160480	108160480	1	0	100.0
ATM:p.Y1475C	chr11	108160516	108160516	1	0	100.0
chr11:108163345-108163520	chr11	108163345	108163520	176	0	100.0
chr11:108164039-108164204	chr11	108164039	108164204	159	7	95.78313253012048
chr11:108165653-108165786	chr11	108165653	108165786	134	0	100.0
chr11:108168013-108168109	chr11	108168013	108168109	97	0	100.0
chr11:108170440-108170612	chr11	108170440	108170612	169	4	97.6878612716763
chr11:108172374-108172516	chr11	108172374	108172516	143	0	100.0
chr11:108173579-108173756	chr11	108173579	108173756	173	5	97.19101123595506
ATM:p.N1801Y	chr11	108173661	108173661	1	0	100.0
chr11:108175401-108175579	chr11	108175401	108175579	175	4	97.76536312849161
chr11:108178623-108178711	chr11	108178623	108178711	89	0	100.0
chr11:108180886-108181042	chr11	108180886	108181042	149	8	94.90445859872611
chr11:108183137-108183225	chr11	108183137	108183225	89	0	100.0
chr11:108186549-108186638	chr11	108186549	108186638	90	0	100.0
chr11:108186737-108186840	chr11	108186737	108186840	104	0	100.0
ATM:p.G2063E	chr11	108186830	108186830	1	0	100.0
chr11:108188099-108188248	chr11	108188099	108188248	146	4	97.33333333333333
chr11:108190680-108190785	chr11	108190680	108190785	106	0	100.0
chr11:108192027-108192147	chr11	108192027	108192147	121	0	100.0
chr11:108196036-108196271	chr11	108196036	108196271	206	30	87.28813559322033
chr11:108196784-108196952	chr11	108196784	108196952	169	0	100.0
chr11:108198371-108198485	chr11	108198371	108198485	115	0	100.0
chr11:108199747-108199965	chr11	108199747	108199965	203	16	92.69406392694064
chr11:108200940-108201148	chr11	108200940	108201148	199	10	95.2153110047847
chr11:108202170-108202284	chr11	108202170	108202284	115	0	100.0
chr11:108202605-108202764	chr11	108202605	108202764	158	2	98.75

ATM:p.Q2593*	chr11	108202753	108202753	1	0	100.0
chr11:108203488-108203627	chr11	108203488	108203627	140	0	100.0
chr11:108204612-108204695	chr11	108204612	108204695	84	0	100.0
chr11:108205695-108205836	chr11	108205695	108205836	142	0	100.0
chr11:108206571-108206688	chr11	108206571	108206688	118	0	100.0
chr11:108213948-108214098	chr11	108213948	108214098	151	0	100.0
chr11:108216469-108216635	chr11	108216469	108216635	131	36	78.44311377245509
chr11:108218005-108218092	chr11	108218005	108218092	88	0	100.0
chr11:108224492-108224607	chr11	108224492	108224607	116	0	100.0
chr11:108225537-108225601	chr11	108225537	108225601	65	0	100.0
chr11:108235808-108235945	chr11	108235808	108235945	138	0	100.0
chr11:108236051-108236250	chr11	108236051	108236250	193	7	96.5
ATM:p.R3008H	chr11	108236087	108236087	1	0	100.0
ATM:p.G3051V	chr11	108236216	108236216	1	0	100.0
chr12:25378540-25378720	chr12	25378540	25378720	178	3	98.34254143646409
chr12:25380160-25380346	chr12	25380160	25380346	179	8	95.72192513368984
chr12:25398190-25398340	chr12	25398190	25398340	151	0	100.0
KMT2D:c.16469_16470delAA	chr12	49415877	49415878	2	0	100.0
KMT2D:p.R5282*	chr12	49418670	49418670	1	0	100.0
KMT2D:p.C5092F	chr12	49420474	49420474	1	0	100.0
KMT2D:c.15251delT	chr12	49420498	49420498	1	0	100.0
KMT2D:p.G5039V	chr12	49420633	49420633	1	0	100.0
KMT2D:c.15029delC	chr12	49420720	49420720	1	0	100.0
KMT2D:p.L4993Q	chr12	49420771	49420771	1	0	100.0
KMT2D:c.14840_14843dupCCTC	chr12	49420906	49420906	1	0	100.0
KMT2D:c.14515+1G>C	chr12	49421791	49421791	1	0	100.0
KMT2D:p.W4730*	chr12	49422905	49422905	1	0	100.0
KMT2D:p.W4730*	chr12	49422906	49422906	1	0	100.0
KMT2D:c.14017_14018delAG	chr12	49423241	49423242	2	0	100.0
KMT2D:p.Q4220*	chr12	49425830	49425830	1	0	100.0
KMT2D:p.G4046E	chr12	49426351	49426351	1	0	100.0
KMT2D:p.Q3971*	chr12	49426577	49426577	1	0	100.0
KMT2D:p.Q3860*	chr12	49426910	49426910	1	0	100.0
KMT2D:c.11065dupG	chr12	49427423	49427423	1	0	100.0
KMT2D:p.Q3575*	chr12	49427867	49427867	1	0	100.0
KMT2D:p.Q3471*	chr12	49428394	49428394	1	0	100.0
KMT2D:c.9185delA	chr12	49431954	49431954	1	0	100.0
KMT2D:p.R2915*	chr12	49432396	49432396	1	0	100.0
KMT2D:p.R2734*	chr12	49433247	49433247	1	0	100.0
KMT2D:c.8118_8119insT	chr12	49433329	49433329	1	0	100.0
KMT2D:p.R2645*	chr12	49433620	49433620	1	0	100.0
KMT2D:p.Q2514*	chr12	49434013	49434013	1	0	100.0
KMT2D:p.R2471*	chr12	49434142	49434142	1	0	100.0
KMT2D:c.6957_6964delGGAGTTAA	chr12	49434589	49434596	8	0	100.0
KMT2D:p.Q2237*	chr12	49434844	49434844	1	0	100.0
KMT2D:c.6285_6348del	chr12	49435205	49435268	64	0	100.0
KMT2D:c.5754_5758delTACGC	chr12	49436548	49436552	5	0	100.0
KMT2D:c.5548_5549delGGinsC	chr12	49436954	49436955	2	0	100.0
KMT2D:p.R1615*	chr12	49438647	49438647	1	0	100.0
KMT2D:c.4785_4786delTG	chr12	49438704	49438705	2	0	100.0
KMT2D:p.Q1545*	chr12	49439908	49439908	1	0	100.0
KMT2D:c.4479delT	chr12	49440147	49440147	1	0	100.0
KMT2D:c.4395dupC	chr12	49440415	49440415	1	0	100.0
KMT2D:p.H1453R	chr12	49440452	49440452	1	0	100.0
KMT2D:p.G1389R	chr12	49441819	49441819	1	0	100.0
KMT2D:c.3225_3226delTG	chr12	49444145	49444146	2	0	100.0
KMT2D:p.P511R	chr12	49445934	49445934	1	0	100.0
KMT2D:p.S504P	chr12	49445956	49445956	1	0	100.0
KMT2D:c.839+2T>A	chr12	49447257	49447257	1	0	100.0
KMT2D:p.G236E	chr12	49447391	49447391	1	0	100.0
STAT6:p.D419G	chr12	57496661	57496661	1	0	100.0
BTG1	chr12	92537855	92538223	328	41	88.88888888888889
BTG1	chr12	92539163	92539311	135	14	90.60402684563758
FOXO1	chr13	41133659	41134997	1139	200	85.06348020911128
FOXO1:p.S502S	chr13	41134122	41134122	1	0	100.0
FOXO1:p.A458V	chr13	41134255	41134255	1	0	100.0
FOXO1:p.N216K	chr13	41134980	41134980	1	0	100.0
FOXO1	chr13	41239719	41240349	158	473	25.039619651347067
B2M:p.M1I	chr15	45003747	45003747	1	0	100.0
B2M:p.L7*	chr15	45003764	45003764	1	0	100.0
B2M:p.L12Q	chr15	45003779	45003779	1	0	100.0
B2M:c.37_40delCTCT	chr15	45003781	45003784	4	0	100.0
B2M:p.C45*	chr15	45007687	45007688	2	0	100.0
B2M:p.C45*	chr15	45007688	45007688	1	0	100.0
B2M:c.302_303delGT	chr15	45007855	45007856	2	0	100.0
chr15:66679675-66679825	chr15	66679675	66679825	151	0	100.0
chr15:66727375-66727575	chr15	66727375	66727575	201	0	100.0
chr15:66729070-66729250	chr15	66729070	66729250	181	0	100.0
chr15:66735610-66735700	chr15	66735610	66735700	91	0	100.0
chr15:66736980-66737060	chr15	66736980	66737060	81	0	100.0
chr15:66774055-66774240	chr15	66774055	66774240	186	0	100.0
chr15:66777320-66777540	chr15	66777320	66777540	210	11	95.02262443438914
chr15:66779550-66779640	chr15	66779550	66779640	91	0	100.0
CREBBP	chr16	3777718	3779875	1647	511	76.32066728452271

CREBBP	chr16	3781192	3781474	244	39	86.21908127208481
CREBBP:c.4792_4793insA	chr16	3781459	3781459	1	0	100.0
CREBBP	chr16	3781776	3781938	163	0	100.0
CREBBP:c.4792_4793insA	chr16	3781875	3781875	1	0	100.0
CREBBP	chr16	3786036	3786204	169	0	100.0
CREBBP:p.L1499R	chr16	3786155	3786155	1	0	100.0
CREBBP	chr16	3786650	3786816	167	0	100.0
CREBBP:p.Y1503H	chr16	3786704	3786704	1	0	100.0
CREBBP:p.Y1503N	chr16	3786704	3786704	1	0	100.0
CREBBP:p.W1502C	chr16	3786705	3786705	1	0	100.0
CREBBP:p.W1502R	chr16	3786707	3786707	1	0	100.0
CREBBP:p.L1499R	chr16	3786715	3786715	1	0	100.0
CREBBP:p.P1488T	chr16	3786749	3786749	1	0	100.0
CREBBP:p.F1484S	chr16	3786760	3786760	1	0	100.0
CREBBP:p.F1484S	chr16	3786760	3786761	2	0	100.0
CREBBP:p.I1483S	chr16	3786763	3786763	1	0	100.0
CREBBP:p.I1483N	chr16	3786763	3786763	1	0	100.0
CREBBP:p.I1483S	chr16	3786763	3786764	2	0	100.0
CREBBP:p.Y1482N	chr16	3786767	3786767	1	0	100.0
CREBBP:c.4394+1G>T	chr16	3788559	3788559	1	0	100.0
CREBBP	chr16	3788559	3788673	115	0	100.0
CREBBP:p.Y1460*	chr16	3788574	3788574	1	0	100.0
CREBBP:p.Y1450N	chr16	3788606	3788606	1	0	100.0
CREBBP:p.Y1450D	chr16	3788606	3788606	1	0	100.0
CREBBP:p.R1446H	chr16	3788617	3788617	1	0	100.0
CREBBP:p.Y1433H	chr16	3788657	3788657	1	0	100.0
CREBBP	chr16	3789578	3789725	148	0	100.0
CREBBP:p.C1408Y	chr16	3789636	3789636	1	0	100.0
CREBBP	chr16	3790399	3790550	152	0	100.0
CREBBP:p.V1371D	chr16	3790421	3790421	1	0	100.0
CREBBP:p.N1350I	chr16	3790484	3790484	1	0	100.0
CREBBP:p.T1332P	chr16	3790539	3790539	1	0	100.0
CREBBP:c.3982+2T>A	chr16	3794893	3794893	1	0	100.0
CREBBP	chr16	3794894	3794962	69	0	100.0
CREBBP	chr16	3795277	3795355	79	0	100.0
CREBBP	chr16	3799627	3799684	58	0	100.0
CREBBP	chr16	3801726	3801807	82	0	100.0
CREBBP	chr16	3807288	3807377	90	0	100.0
CREBBP	chr16	3807809	3808049	203	38	84.23236514522821
CREBBP:c.3565delA	chr16	3807854	3807854	1	0	100.0
CREBBP	chr16	3808854	3808973	120	0	100.0
CREBBP:p.Q1104*	chr16	3808914	3808914	1	0	100.0
CREBBP	chr16	3817720	3817910	178	13	93.19371727748691
CREBBP:c.3250_3250+1insA	chr16	3817721	3817721	1	0	100.0
CREBBP	chr16	3819174	3819354	181	0	100.0
CREBBP	chr16	3820570	3820987	380	38	90.9090909090909
CREBBP	chr16	3823751	3823931	181	0	100.0
CREBBP	chr16	3824569	3824694	126	0	100.0
CREBBP	chr16	3827613	3827658	46	0	100.0
CREBBP	chr16	3828011	3828183	159	14	91.90751445086705
CREBBP	chr16	3828700	3828818	119	0	100.0
CREBBP	chr16	3830732	3830879	148	0	100.0
CREBBP	chr16	3831204	3831307	104	0	100.0
CREBBP	chr16	3832684	3832927	210	34	86.06557377049181
CREBBP	chr16	3841981	3842095	115	0	100.0
CREBBP	chr16	3843386	3843627	206	36	85.12396694214875
CREBBP	chr16	3860603	3860780	178	0	100.0
CREBBP	chr16	3900297	3901010	601	113	84.17366946778712
CREBBP	chr16	3929832	3929917	86	0	100.0
SOCS1	chr16	11348273	11349335	772	291	72.62464722483537
SOCS1:p.Q210H	chr16	11348706	11348706	1	0	100.0
SOCS1:p.F207F	chr16	11348715	11348715	1	0	100.0
SOCS1:p.S206P	chr16	11348720	11348720	1	0	100.0
SOCS1:p.S205N	chr16	11348722	11348722	1	0	100.0
TP53	chr17	7572926	7573008	83	0	100.0
TP53:p.D259E	chr17	7572936	7572936	1	0	100.0
TP53:p.D259D	chr17	7572936	7572936	1	0	100.0
TP53:p.D352N	chr17	7572938	7572938	1	0	100.0
TP53:c.724_739del	chr17	7572973	7572988	16	0	100.0
TP53	chr17	7573926	7574033	108	0	100.0
TP53:p.E358K	chr17	7573955	7573955	1	0	100.0
TP53:p.D352N	chr17	7573973	7573973	1	0	100.0
TP53:c.522_539del	chr17	7574011	7574028	18	0	100.0
TP53	chr17	7576536	7576584	49	0	100.0
TP53:c.522_539del	chr17	7576562	7576579	18	0	100.0
TP53	chr17	7576624	7576657	34	0	100.0
TP53:c.522_539del	chr17	7576635	7576652	18	0	100.0
TP53	chr17	7576852	7576926	75	0	100.0
TP53:c.448_460del	chr17	7576909	7576921	13	0	100.0
TP53:c.522_539del	chr17	7576911	7577020	71	39	64.54545454545455
TP53	chr17	7577018	7577155	138	0	100.0
TP53:p.K305N	chr17	7577023	7577023	1	0	100.0
TP53:p.P142H	chr17	7577035	7577036	2	0	100.0
TP53:p.E298K	chr17	7577046	7577046	1	0	100.0

TP53:p.E294*	chr17	7577058	7577058	1	0	100.0
TP53:p.K132N	chr17	7577065	7577065	1	0	100.0
TP53:p.K132E	chr17	7577067	7577067	1	0	100.0
TP53:p.R158H	chr17	7577069	7577069	1	0	100.0
TP53:p.R290H	chr17	7577069	7577069	1	0	100.0
TP53:p.R290C	chr17	7577070	7577070	1	0	100.0
TP53:p.R158C	chr17	7577070	7577070	1	0	100.0
TP53:c.862_863delAA	chr17	7577075	7577076	2	0	100.0
TP53:p.E286G	chr17	7577081	7577081	1	0	100.0
TP53:p.E286V	chr17	7577081	7577081	1	0	100.0
TP53:c.448_460del	chr17	7577082	7577094	13	0	100.0
TP53:c.724_739del	chr17	7577082	7577097	16	0	100.0
TP53:p.E285K	chr17	7577085	7577085	1	0	100.0
TP53:c.455delC	chr17	7577087	7577087	1	0	100.0
TP53:p.R283L	chr17	7577090	7577090	1	0	100.0
TP53:p.R282Q	chr17	7577093	7577093	1	0	100.0
TP53:p.R282W	chr17	7577094	7577094	1	0	100.0
TP53:p.R280S	chr17	7577098	7577098	1	0	100.0
TP53:p.R280I	chr17	7577099	7577099	1	0	100.0
TP53:p.R280S	chr17	7577099	7577100	2	0	100.0
TP53:p.C238Y	chr17	7577108	7577108	1	0	100.0
TP53:p.C277F	chr17	7577108	7577108	1	0	100.0
TP53:p.R273P	chr17	7577120	7577120	1	0	100.0
TP53:p.R273C	chr17	7577121	7577121	1	0	100.0
TP53:p.S261R	chr17	7577155	7577155	1	0	100.0
TP53	chr17	7577498	7577608	111	0	100.0
TP53:p.S261R	chr17	7577500	7577500	1	0	100.0
TP53:p.D259E	chr17	7577504	7577504	1	0	100.0
TP53:p.D259D	chr17	7577504	7577504	1	0	100.0
TP53:p.L257P	chr17	7577511	7577511	1	0	100.0
TP53:p.I254S	chr17	7577520	7577520	1	0	100.0
TP53:p.I254N	chr17	7577520	7577520	1	0	100.0
TP53:p.I254S	chr17	7577520	7577521	2	0	100.0
TP53:p.I254V	chr17	7577521	7577521	1	0	100.0
TP53:p.I251F	chr17	7577530	7577530	1	0	100.0
TP53:p.P250N	chr17	7577532	7577533	2	0	100.0
TP53:p.R249K	chr17	7577535	7577535	1	0	100.0
TP53:p.R249G	chr17	7577536	7577536	1	0	100.0
TP53:p.R248Q	chr17	7577538	7577538	1	0	100.0
TP53:p.R248W	chr17	7577539	7577539	1	0	100.0
TP53:p.R248G	chr17	7577539	7577539	1	0	100.0
TP53:c.448_460del	chr17	7577542	7577554	13	0	100.0
TP53:c.724_739del	chr17	7577542	7577557	16	0	100.0
TP53:p.M246V	chr17	7577545	7577545	1	0	100.0
TP53:p.G245D	chr17	7577547	7577547	1	0	100.0
TP53:p.G245V	chr17	7577547	7577547	1	0	100.0
TP53:p.G245S	chr17	7577547	7577548	2	0	100.0
TP53:p.G245S	chr17	7577548	7577548	1	0	100.0
TP53:p.G244G	chr17	7577549	7577549	1	0	100.0
TP53:p.G244D	chr17	7577550	7577550	1	0	100.0
TP53:p.S241P	chr17	7577560	7577560	1	0	100.0
TP53:p.S240R	chr17	7577561	7577561	1	0	100.0
TP53:p.S240R	chr17	7577563	7577563	1	0	100.0
TP53:p.N239D	chr17	7577566	7577566	1	0	100.0
TP53:p.C238Y	chr17	7577568	7577568	1	0	100.0
TP53:p.M237I	chr17	7577570	7577570	1	0	100.0
TP53:p.Y234N	chr17	7577581	7577581	1	0	100.0
TP53:p.C229*	chr17	7577594	7577594	1	0	100.0
TP53:p.C229*	chr17	7577594	7577595	2	0	100.0
TP53:p.G187S	chr17	7577604	7577605	2	0	100.0
TP53:p.G187S	chr17	7577605	7577605	1	0	100.0
TP53	chr17	7578176	7578289	114	0	100.0
TP53:p.E221K	chr17	7578188	7578188	1	0	100.0
TP53:p.Y220S	chr17	7578190	7578190	1	0	100.0
TP53:p.Y220C	chr17	7578190	7578190	1	0	100.0
TP53:p.Y220S	chr17	7578190	7578191	2	0	100.0
TP53:c.522_539del	chr17	7578193	7578210	18	0	100.0
TP53:p.V218V	chr17	7578195	7578195	1	0	100.0
TP53:p.V218G	chr17	7578196	7578196	1	0	100.0
TP53:p.V216E	chr17	7578202	7578202	1	0	100.0
TP53:p.V216M	chr17	7578203	7578203	1	0	100.0
TP53:p.S215R	chr17	7578204	7578204	1	0	100.0
TP53:p.S215I	chr17	7578205	7578205	1	0	100.0
TP53:p.S215R	chr17	7578206	7578206	1	0	100.0
TP53:p.R213L	chr17	7578211	7578211	1	0	100.0
TP53:p.R213*	chr17	7578212	7578212	1	0	100.0
TP53:p.T211I	chr17	7578217	7578217	1	0	100.0
TP53:p.D208V	chr17	7578226	7578226	1	0	100.0
TP53:p.Y205C	chr17	7578235	7578235	1	0	100.0
TP53:p.Y205H	chr17	7578236	7578236	1	0	100.0
TP53:p.R196*	chr17	7578263	7578263	1	0	100.0
TP53:p.I195L	chr17	7578264	7578266	3	0	100.0
TP53:c.577_585delCATCTTATC	chr17	7578264	7578272	9	0	100.0
TP53:p.I195L	chr17	7578266	7578266	1	0	100.0

TP53:p.H193R	chr17	7578271	7578271	1	0	100.0
TP53:p.H193P	chr17	7578271	7578271	1	0	100.0
TP53:p.H193N	chr17	7578272	7578272	1	0	100.0
TP53:p.H193D	chr17	7578272	7578272	1	0	100.0
TP53:c.448_460del	chr17	7578272	7578284	13	0	100.0
TP53:c.455delC	chr17	7578278	7578278	1	0	100.0
TP53:p.G187S	chr17	7578289	7578289	1	0	100.0
TP53	chr17	7578370	7578554	185	0	100.0
TP53:p.G187S	chr17	7578371	7578371	1	0	100.0
TP53:c.522_539del	chr17	7578384	7578401	18	0	100.0
TP53:p.H179Q	chr17	7578393	7578393	1	0	100.0
TP53:p.C176R	chr17	7578402	7578404	3	0	100.0
TP53:p.C176F	chr17	7578403	7578403	1	0	100.0
TP53:p.C176Y	chr17	7578403	7578403	1	0	100.0
TP53:p.C176G	chr17	7578404	7578404	1	0	100.0
TP53:p.C176R	chr17	7578404	7578404	1	0	100.0
TP53:p.R175P	chr17	7578406	7578406	1	0	100.0
TP53:p.R175H	chr17	7578406	7578406	1	0	100.0
TP53:p.V173A	chr17	7578412	7578412	1	0	100.0
TP53:p.V173L	chr17	7578413	7578413	1	0	100.0
TP53:p.V173M	chr17	7578413	7578413	1	0	100.0
TP53:p.E171*	chr17	7578419	7578419	1	0	100.0
TP53:p.Q167H	chr17	7578429	7578429	1	0	100.0
TP53:p.I162I	chr17	7578444	7578444	1	0	100.0
TP53:p.R158H	chr17	7578457	7578457	1	0	100.0
TP53:p.R158C	chr17	7578458	7578458	1	0	100.0
TP53:p.V157G	chr17	7578460	7578460	1	0	100.0
TP53:p.V157F	chr17	7578461	7578461	1	0	100.0
TP53:c.448_460del	chr17	7578470	7578482	13	0	100.0
TP53:c.455delC	chr17	7578475	7578475	1	0	100.0
TP53:p.S149S	chr17	7578483	7578483	1	0	100.0
TP53:p.S149S	chr17	7578484	7578485	2	0	100.0
TP53:p.D148E	chr17	7578486	7578486	1	0	100.0
TP53:p.W146*	chr17	7578492	7578492	1	0	100.0
TP53:p.W146*	chr17	7578493	7578493	1	0	100.0
TP53:p.L145P	chr17	7578496	7578496	1	0	100.0
TP53:p.V143V	chr17	7578501	7578501	1	0	100.0
TP53:p.V143M	chr17	7578503	7578503	1	0	100.0
TP53:p.P142H	chr17	7578505	7578505	1	0	100.0
TP53:p.C141R	chr17	7578507	7578509	3	0	100.0
TP53:p.C141R	chr17	7578509	7578509	1	0	100.0
TP53:p.K132N	chr17	7578513	7578513	1	0	100.0
TP53:p.K132E	chr17	7578515	7578515	1	0	100.0
TP53:p.A138T	chr17	7578518	7578518	1	0	100.0
TP53:p.C135F	chr17	7578526	7578526	1	0	100.0
TP53:p.K132N	chr17	7578534	7578534	1	0	100.0
TP53:c.391_393delAAC	chr17	7578535	7578537	3	0	100.0
TP53:p.K132E	chr17	7578536	7578536	1	0	100.0
TP53	chr17	7579311	7579590	240	40	85.71428571428571
TP53	chr17	7579699	7579721	23	0	100.0
TP53	chr17	7579838	7579912	75	0	100.0
chr17:40474290-40474515	chr17	40474290	40474515	207	19	91.59292035398231
chr17:40475015-40475170	chr17	40475015	40475170	156	0	100.0
chr17:40476970-40477090	chr17	40476970	40477090	121	0	100.0
chr17:40478120-40478230	chr17	40478120	40478230	111	0	100.0
chr17:40481420-40481480	chr17	40481420	40481480	61	0	100.0
chr17:40481560-40481670	chr17	40481560	40481670	111	0	100.0
chr17:40481760-40481800	chr17	40481760	40481800	41	0	100.0
chr17:40483480-40483560	chr17	40483480	40483560	81	0	100.0
chr17:40485670-40485800	chr17	40485670	40485800	131	0	100.0
chr17:62006580-62006700	chr17	62006580	62006700	121	0	100.0
CD79B:c.619_620insT	chr17	62006657	62006657	1	0	100.0
CD79B:c.619_620insT	chr17	62006660	62006660	1	0	100.0
CD79B:c.612_613insA	chr17	62006664	62006664	1	0	100.0
CD79B:c.612_613insA	chr17	62006667	62006667	1	0	100.0
CD79B:c.596delT	chr17	62006680	62006680	1	0	100.0
CD79B:p.L199R	chr17	62006680	62006680	1	0	100.0
chr17:62006780-62006840	chr17	62006780	62006840	61	0	100.0
CD79B:p.Y196R	chr17	62006797	62006799	3	0	100.0
CD79B:p.Y196F	chr17	62006798	62006798	1	0	100.0
CD79B:p.Y196C	chr17	62006798	62006798	1	0	100.0
CD79B:p.Y196S	chr17	62006798	62006798	1	0	100.0
CD79B:p.Y196R	chr17	62006798	62006799	2	0	100.0
CD79B:p.Y196S	chr17	62006798	62006799	2	0	100.0
CD79B:p.Y196H	chr17	62006799	62006799	1	0	100.0
CD79B:p.Y196N	chr17	62006799	62006799	1	0	100.0
CD79B:p.E192*	chr17	62006811	62006811	1	0	100.0
CD79B:p.E191K	chr17	62006814	62006814	1	0	100.0
CD79B:p.E192*	chr17	62006814	62006814	1	0	100.0
CD79B:c.550-1G>A	chr17	62006836	62006836	1	0	100.0
chr17:62007120-62007260	chr17	62007120	62007260	141	0	100.0
CD79B:p.D159N	chr17	62007204	62007204	1	0	100.0
CD79B:c.102_105delGTAC	chr17	62008708	62008711	4	0	100.0
GNA13	chr17	63010374	63010947	487	87	84.84320557491289

GNA13:c.561+2T>A	chr17	63014369	63014369	1	0	100.0
GNA13	chr17	63014370	63014421	52	0	100.0
GNA13	chr17	63049619	63049846	202	26	88.59649122807018
GNA13:p.I158K	chr17	63049657	63049657	1	0	100.0
GNA13	chr17	63052428	63052711	214	70	75.35211267605634
GNA13:p.S31F	chr17	63052620	63052620	1	0	100.0
BCL2	chr18	60795857	60795992	136	0	100.0
BCL2:p.S213A	chr18	60795941	60795941	1	0	100.0
BCL2	chr18	60985186	60985899	458	256	64.14565826330532
BCL2:p.H184N	chr18	60985350	60985350	1	0	100.0
BCL2:p.E179D	chr18	60985363	60985363	1	0	100.0
BCL2:p.A174V	chr18	60985379	60985379	1	0	100.0
BCL2:p.I173V	chr18	60985383	60985383	1	0	100.0
BCL2:p.E165D	chr18	60985405	60985405	1	0	100.0
BCL2:p.N163K	chr18	60985411	60985411	1	0	100.0
BCL2:p.N163T	chr18	60985412	60985412	1	0	100.0
BCL2:p.E136D	chr18	60985492	60985492	1	0	100.0
BCL2:p.V134A	chr18	60985499	60985499	1	0	100.0
BCL2:p.A131D	chr18	60985508	60985508	1	0	100.0
BCL2:p.A131V	chr18	60985508	60985508	1	0	100.0
BCL2:p.A131G	chr18	60985508	60985508	1	0	100.0
BCL2:p.A131T	chr18	60985509	60985509	1	0	100.0
BCL2:p.R129H	chr18	60985514	60985514	1	0	100.0
BCL2:p.L119V	chr18	60985545	60985545	1	0	100.0
BCL2:p.Q118R	chr18	60985547	60985547	1	0	100.0
BCL2:p.A45T	chr18	60985767	60985767	1	0	100.0
BCL2:p.G33R	chr18	60985803	60985803	1	0	100.0
BCL2:p.D31E	chr18	60985807	60985807	1	0	100.0
BCL2:p.W30C	chr18	60985810	60985810	1	0	100.0
BCL2:p.K22R	chr18	60985835	60985835	1	0	100.0
BCL2:p.K17E	chr18	60985851	60985851	1	0	100.0
BCL2:p.T7I	chr18	60985880	60985880	1	0	100.0
BCL2:p.T7P	chr18	60985881	60985881	1	0	100.0
BCL2:p.T7A	chr18	60985881	60985881	1	0	100.0
BCL2:p.R6T	chr18	60985883	60985883	1	0	100.0
BCL2:p.R6G	chr18	60985884	60985884	1	0	100.0
BCL2:p.G5V	chr18	60985886	60985886	1	0	100.0
BCL2:p.A4V	chr18	60985889	60985889	1	0	100.0
BCL2:p.A4G	chr18	60985889	60985889	1	0	100.0
BCL2:p.H3D	chr18	60985893	60985893	1	0	100.0
BCL2:p.A2S	chr18	60985894	60985896	3	0	100.0
BCL2:p.A2S	chr18	60985896	60985896	1	0	100.0
BCL2:p.A2T	chr18	60985896	60985896	1	0	100.0
chr19:42384730-42384810	chr19	42384730	42384810	81	0	100.0
chr19:42384925-42385050	chr19	42384925	42385050	126	0	100.0
CD79A:c.568-2A>G	chr19	42384932	42384932	1	0	100.0
EP300	chr22	41489008	41489102	95	0	100.0
EP300	chr22	41513190	41513825	557	79	87.57861635220125
EP300	chr22	41521867	41522044	159	19	89.32584269662921
EP300	chr22	41523490	41523752	225	38	85.55133079847909
EP300	chr22	41525893	41526007	115	0	100.0
EP300:p.L415P	chr22	41525969	41525969	1	0	100.0
EP300	chr22	41527391	41527637	209	38	84.61538461538461
EP300	chr22	41531816	41531910	95	0	100.0
EP300	chr22	41533656	41533794	139	0	100.0
EP300	chr22	41536143	41536261	119	0	100.0
EP300	chr22	41537051	41537226	170	6	96.5909090909091
EP300	chr22	41542742	41542820	79	0	100.0
EP300	chr22	41543840	41543950	111	0	100.0
EP300	chr22	41545041	41545179	139	0	100.0
EP300:c.2379+2T>G	chr22	41545181	41545181	1	0	100.0
EP300	chr22	41545764	41546202	393	46	89.52164009111617
EP300	chr22	41547836	41548016	181	0	100.0
EP300	chr22	41548209	41548354	146	0	100.0
EP300	chr22	41550998	41551117	120	0	100.0
EP300	chr22	41553172	41553412	205	36	85.06224066390041
EP300:c.3442_3447delGTCTTT	chr22	41553353	41553358	6	0	100.0
EP300	chr22	41554415	41554504	90	0	100.0
EP300	chr22	41556645	41556726	82	0	100.0
EP300	chr22	41558726	41558783	58	0	100.0
EP300	chr22	41560056	41560134	79	0	100.0
EP300	chr22	41562602	41562670	69	0	100.0
EP300	chr22	41564452	41564603	152	0	100.0
EP300	chr22	41564724	41564871	148	0	100.0
EP300	chr22	41565506	41565620	115	0	100.0
EP300	chr22	41566409	41566575	167	0	100.0
EP300:p.W1466G	chr22	41566519	41566519	1	0	100.0
EP300:p.Y1467N	chr22	41566522	41566522	1	0	100.0
EP300	chr22	41568502	41568667	166	0	100.0
EP300	chr22	41569626	41569788	163	0	100.0
EP300	chr22	41572250	41572532	246	37	86.92579505300354
EP300	chr22	41572776	41574960	1828	357	83.66132723112128

Supplementary Table 4. Commercial myeloid Mission Bio panel covering hotspot mutations in 46 genes (312 amplicons) and 19 DNA-barcoded antibodies.

46-gene clonal hematopoiesis panel					
ASXL1	BCOR	BRAF	CALR	CBFB	CBL
CYP4F3	CHEK2	DNMT3A	ETV6	EZH2	FLT3
GATA2	IDH1	IDH2	IL6R	IP6K1	JAK2
KIT	KMT2A	KRAS	MEIS2	MYC	MYH11
NF1	NPM1	NRAS	PHF6	PPM1D	PTPN11
RAD21	RUNX1	SETBP1	SF3A1	SF3B1	SMC1A
SRSF2	STAG2	TET2	TP53	TRPC4	U2AF1
UBA1	WT1	ZEB2	ZRSR2		

19 DNA-barcoded antibodies					
CD2	CD3	CD7	CD10	CD11b	CD117
CD34	CD38	CD45RA	CD56	CD13	CD123
CD14	HLA-DR	CD19	CD33	CD22	2 isotype controls

Supplementary Table 5. Amplicon coverage of genes included in the myeloid-targeted scDNA-seq panel.

target	chr	start	end	b_covered	b_missed	coverage_perc
chr1:36932124-36932136	chr1	36932124	36932136	13	0	100.0
chr1:36932218-36932229	chr1	36932218	36932229	12	0	100.0
chr1:36932236-36932247	chr1	36932236	36932247	12	0	100.0
chr1:36932248-36932259	chr1	36932248	36932259	12	0	100.0
chr1:36933192-36933203	chr1	36933192	36933203	12	0	100.0
chr1:36933428-36933439	chr1	36933428	36933439	12	0	100.0
chr1:43803517-43803528	chr1	43803517	43803528	12	0	100.0
chr1:43803794-43803805	chr1	43803794	43803805	12	0	100.0
chr1:43805154-43805165	chr1	43805154	43805165	12	0	100.0
chr1:43805713-43805724	chr1	43805713	43805724	12	0	100.0
chr1:43815002-43815013	chr1	43815002	43815013	12	0	100.0
chr1:43815002-43815014	chr1	43815002	43815014	13	0	100.0
chr1:43815003-43815014	chr1	43815003	43815014	12	0	100.0
chr1:43818300-43818311	chr1	43818300	43818311	12	0	100.0
chr1:43818304-43818315	chr1	43818304	43818315	12	0	100.0
chr1:43818425-43818436	chr1	43818425	43818436	12	0	100.0
chr1:115252198-115252209	chr1	115252198	115252209	12	0	100.0
chr1:115256515-115256526	chr1	115256515	115256526	12	0	100.0
chr1:115256522-115256533	chr1	115256522	115256533	12	0	100.0
chr1:115256523-115256534	chr1	115256523	115256534	12	0	100.0
chr1:115256524-115256535	chr1	115256524	115256535	12	0	100.0
chr1:115258668-115258679	chr1	115258668	115258679	12	0	100.0
chr1:115258738-115258749	chr1	115258738	115258749	12	0	100.0
chr1:115258739-115258750	chr1	115258739	115258750	12	0	100.0
chr1:115258741-115258752	chr1	115258741	115258752	12	0	100.0
chr1:115258742-115258753	chr1	115258742	115258753	12	0	100.0
chr1:154380480-154380490	chr1	154380480	154380490	11	0	100.0
chr2:25457170-25457181	chr2	25457170	25457181	12	0	100.0
chr2:25457186-25457197	chr2	25457186	25457197	12	0	100.0
chr2:25457236-25457247	chr2	25457236	25457247	12	0	100.0
chr2:25457237-25457248	chr2	25457237	25457248	12	0	100.0
chr2:25458540-25458550	chr2	25458540	25458550	11	0	100.0
chr2:25458589-25458600	chr2	25458589	25458600	12	0	100.0
chr2:25458604-25458618	chr2	25458604	25458618	15	0	100.0
chr2:25458682-25458693	chr2	25458682	25458693	12	0	100.0
chr2:25459798-25459809	chr2	25459798	25459809	12	0	100.0
chr2:25459843-25459854	chr2	25459843	25459854	12	0	100.0
chr2:25461998-25462009	chr2	25461998	25462009	12	0	100.0
chr2:25462006-25462017	chr2	25462006	25462017	12	0	100.0
chr2:25462051-25462062	chr2	25462051	25462062	12	0	100.0
chr2:25463175-25463186	chr2	25463175	25463186	12	0	100.0
chr2:25463176-25463187	chr2	25463176	25463187	12	0	100.0
chr2:25463178-25463189	chr2	25463178	25463189	12	0	100.0
chr2:25463280-25463291	chr2	25463280	25463291	12	0	100.0
chr2:25463292-25463305	chr2	25463292	25463305	14	0	100.0
chr2:25463302-25463313	chr2	25463302	25463313	12	0	100.0
chr2:25463477-25463487	chr2	25463477	25463487	11	0	100.0
chr2:25463526-25463537	chr2	25463526	25463537	12	0	100.0
chr2:25463529-25463540	chr2	25463529	25463540	12	0	100.0

chr2:25463530-25463541	chr2	25463530	25463541	12	0	100.0
chr2:25463535-25463546	chr2	25463535	25463546	12	0	100.0
chr2:25463561-25463572	chr2	25463561	25463572	12	0	100.0
chr2:25463573-25463584	chr2	25463573	25463584	12	0	100.0
chr2:25463577-25463588	chr2	25463577	25463588	12	0	100.0
chr2:25463578-25463589	chr2	25463578	25463589	12	0	100.0
chr2:25463595-25463606	chr2	25463595	25463606	12	0	100.0
chr2:25464444-25464455	chr2	25464444	25464455	12	0	100.0
chr2:25464499-25464511	chr2	25464499	25464511	13	0	100.0
chr2:25464502-25464513	chr2	25464502	25464513	12	0	100.0
chr2:25464520-25464531	chr2	25464520	25464531	12	0	100.0
chr2:25464527-25464539	chr2	25464527	25464539	13	0	100.0
chr2:25464531-25464542	chr2	25464531	25464542	12	0	100.0
chr2:25464538-25464549	chr2	25464538	25464549	12	0	100.0
chr2:25466760-25466771	chr2	25466760	25466771	12	0	100.0
chr2:25466791-25466802	chr2	25466791	25466802	12	0	100.0
chr2:25466793-25466804	chr2	25466793	25466804	12	0	100.0
chr2:25466794-25466805	chr2	25466794	25466805	12	0	100.0
chr2:25467036-25467059	chr2	25467036	25467059	24	0	100.0
chr2:25467067-25467078	chr2	25467067	25467078	12	0	100.0
chr2:25467135-25467146	chr2	25467135	25467146	12	0	100.0
chr2:25467201-25467212	chr2	25467201	25467212	12	0	100.0
chr2:25467402-25467413	chr2	25467402	25467413	12	0	100.0
chr2:25467430-25467441	chr2	25467430	25467441	12	0	100.0
chr2:25467442-25467453	chr2	25467442	25467453	12	0	100.0
chr2:25467443-25467454	chr2	25467443	25467454	12	0	100.0
chr2:25467462-25467473	chr2	25467462	25467473	12	0	100.0
chr2:25467464-25467476	chr2	25467464	25467476	13	0	100.0
chr2:25467471-25467484	chr2	25467471	25467484	14	0	100.0
chr2:25467476-25467489	chr2	25467476	25467489	14	0	100.0
chr2:25467487-25467498	chr2	25467487	25467498	12	0	100.0
chr2:25468115-25468126	chr2	25468115	25468126	12	0	100.0
chr2:25468154-25468168	chr2	25468154	25468168	15	0	100.0
chr2:25468168-25468179	chr2	25468168	25468179	120	0	100.0
chr2:25468882-25468893	chr2	25468882	25468893	12	0	100.0
chr2:25468886-25468897	chr2	25468886	25468897	12	0	100.0
chr2:25468928-25468939	chr2	25468928	25468939	12	0	100.0
chr2:25469019-25469035	chr2	25469019	25469035	17	0	100.0
chr2:25469021-25469032	chr2	25469021	25469032	12	0	100.0
chr2:25469524-25469535	chr2	25469524	25469535	12	0	100.0
chr2:25469525-25469536	chr2	25469525	25469536	12	0	100.0
chr2:25469913-25469924	chr2	25469913	25469924	12	0	100.0
chr2:25469918-25469930	chr2	25469918	25469930	13	0	100.0
chr2:25469952-25469963	chr2	25469952	25469963	12	0	100.0
chr2:25469970-25469981	chr2	25469970	25469981	12	0	100.0
chr2:25470491-25470502	chr2	25470491	25470502	12	0	100.0
chr2:25470492-25470503	chr2	25470492	25470503	12	0	100.0
chr2:25470526-25470537	chr2	25470526	25470537	12	0	100.0
chr2:25470588-25470599	chr2	25470588	25470599	12	0	100.0
chr2:25470613-25470624	chr2	25470613	25470624	12	0	100.0
chr2:25470899-25470910	chr2	25470899	25470910	12	0	100.0
chr2:145164660-145164670	chr2	145164660	145164670	11	0	100.0
chr2:198265520-198265530	chr2	198265520	198265530	11	0	100.0
chr2:198265544-198265555	chr2	198265544	198265555	12	0	100.0
chr2:198266476-198266489	chr2	198266476	198266489	14	0	100.0
chr2:198266599-198266610	chr2	198266599	198266610	12	0	100.0
chr2:198266605-198266616	chr2	198266605	198266616	12	0	100.0
chr2:198266707-198266718	chr2	198266707	198266718	12	0	100.0
chr2:198266828-198266839	chr2	198266828	198266839	12	0	100.0
chr2:198267353-198267364	chr2	198267353	198267364	12	0	100.0
chr2:198267354-198267365	chr2	198267354	198267365	12	0	100.0
chr2:198267365-198267376	chr2	198267365	198267376	12	0	100.0
chr2:198267366-198267377	chr2	198267366	198267377	12	0	100.0
chr2:198267379-198267390	chr2	198267379	198267390	12	0	100.0
chr2:198267478-198267489	chr2	198267478	198267489	12	0	100.0
chr2:198267485-198267496	chr2	198267485	198267496	12	0	100.0
chr2:198267699-198267710	chr2	198267699	198267710	12	0	100.0
chr2:198283227-198283238	chr2	198283227	198283238	12	0	100.0
chr2:209113106-209113117	chr2	209113106	209113117	12	0	100.0
chr2:209113107-209113118	chr2	209113107	209113118	12	0	100.0
MYD88	chr3	38180152	38180519	329	39	89.402173913
chr3:38180347-38180357	chr3	38180347	38180357	11	0	100.0
MYD88	chr3	38181354	38181489	136	0	100.0
chr3:38181418-38181428	chr3	38181418	38181428	11	0	100.0
MYD88	chr3	38181878	38182083	206	0	100.0
MYD88	chr3	38182247	38182339	93	0	100.0
chr3:38182254-38182264	chr3	38182254	38182264	11	0	100.0
MYD88	chr3	38182622	38182777	156	0	100.0
chr3:38182636-38182646	chr3	38182636	38182646	11	0	100.0
chr3:38182752-38182762	chr3	38182752	38182762	11	0	100.0
chr3:49784771-49784781	chr3	49784771	49784781	11	0	100.0
chr3:128200129-128200142	chr3	128200129	128200142	14	0	100.0
chr3:128200130-128200141	chr3	128200130	128200141	12	0	100.0
chr3:128200133-128200149	chr3	128200133	128200149	17	0	100.0
chr3:128200134-128200150	chr3	128200134	128200150	17	0	100.0
chr3:128200676-128200687	chr3	128200676	128200687	12	0	100.0
chr3:128200685-128200696	chr3	128200685	128200696	12	0	100.0
chr3:128200714-128200725	chr3	128200714	128200725	12	0	100.0
chr3:128200715-128200726	chr3	128200715	128200726	12	0	100.0
chr3:128200721-128200732	chr3	128200721	128200732	12	0	100.0

chr3:128200724-128200735	chr3	128200724	128200735	12	0	100.0
chr3:128200730-128200741	chr3	128200730	128200741	12	0	100.0
chr3:128200736-128200748	chr3	128200736	128200748	13	0	100.0
chr3:128202752-128202763	chr3	128202752	128202763	12	0	100.0
chr3:128202761-128202773	chr3	128202761	128202773	13	0	100.0
chr3:128202765-128202776	chr3	128202765	128202776	12	0	100.0
chr3:128202791-128202802	chr3	128202791	128202802	12	0	100.0
chr3:128202794-128202805	chr3	128202794	128202805	12	0	100.0
chr3:128202800-128202811	chr3	128202800	128202811	12	0	100.0
chr3:128204836-128204847	chr3	128204836	128204847	12	0	100.0
chr3:128205188-128205199	chr3	128205188	128205199	12	0	100.0
chr3:128205646-128205658	chr3	128205646	128205658	13	0	100.0
chr4:55564663-55564674	chr4	55564663	55564674	12	0	100.0
chr4:55594191-55594202	chr4	55594191	55594202	12	0	100.0
chr4:55599314-55599325	chr4	55599314	55599325	12	0	100.0
chr4:55599315-55599326	chr4	55599315	55599326	12	0	100.0
chr4:55599334-55599345	chr4	55599334	55599345	12	0	100.0
chr4:106155264-106155276	chr4	106155264	106155276	13	0	100.0
chr4:106155343-106155354	chr4	106155343	106155354	12	0	100.0
chr4:106155406-106155417	chr4	106155406	106155417	12	0	100.0
chr4:106155433-106155444	chr4	106155433	106155444	12	0	100.0
chr4:106155437-106155448	chr4	106155437	106155448	12	0	100.0
chr4:106155502-106155513	chr4	106155502	106155513	12	0	100.0
chr4:106155534-106155546	chr4	106155534	106155546	13	0	100.0
chr4:106155559-106155570	chr4	106155559	106155570	11	1	91.6666666667
chr4:106155657-106155694	chr4	106155657	106155694	38	0	100.0
chr4:106155743-106155754	chr4	106155743	106155754	12	0	100.0
chr4:106155747-106155758	chr4	106155747	106155758	12	0	100.0
chr4:106155772-106155784	chr4	106155772	106155784	13	0	100.0
chr4:106155823-106155834	chr4	106155823	106155834	1	11	8.33333333333
chr4:106155846-106155858	chr4	106155846	106155858	13	0	100.0
chr4:106155870-106155881	chr4	106155870	106155881	12	0	100.0
chr4:106155932-106155944	chr4	106155932	106155944	13	0	100.0
chr4:106155939-106155951	chr4	106155939	106155951	13	0	100.0
chr4:106155979-106155991	chr4	106155979	106155991	13	0	100.0
chr4:106156037-106156048	chr4	106156037	106156048	8	4	66.6666666667
chr4:106156042-106156053	chr4	106156042	106156053	12	0	100.0
chr4:106156066-106156077	chr4	106156066	106156077	12	0	100.0
chr4:106156089-106156100	chr4	106156089	106156100	12	0	100.0
chr4:106156128-106156140	chr4	106156128	106156140	13	0	100.0
chr4:106156158-106156170	chr4	106156158	106156170	13	0	100.0
chr4:106156174-106156185	chr4	106156174	106156185	12	0	100.0
chr4:106156240-106156251	chr4	106156240	106156251	11	1	91.6666666667
chr4:106156307-106156318	chr4	106156307	106156318	12	0	100.0
chr4:106156345-106156359	chr4	106156345	106156359	15	0	100.0
chr4:106156359-106156370	chr4	106156359	106156370	12	0	100.0
chr4:106156390-106156401	chr4	106156390	106156401	12	0	100.0
chr4:106156436-106156447	chr4	106156436	106156447	12	0	100.0
chr4:106156453-106156464	chr4	106156453	106156464	12	0	100.0
chr4:106156621-106156632	chr4	106156621	106156632	12	0	100.0
chr4:106156671-106156682	chr4	106156671	106156682	12	0	100.0
chr4:106156723-106156734	chr4	106156723	106156734	12	0	100.0
chr4:106156741-106156752	chr4	106156741	106156752	12	0	100.0
chr4:106156752-106156764	chr4	106156752	106156764	13	0	100.0
chr4:106156813-106156824	chr4	106156813	106156824	12	0	100.0
chr4:106156821-106156835	chr4	106156821	106156835	15	0	100.0
chr4:106156822-106156833	chr4	106156822	106156833	12	0	100.0
chr4:106156859-106156871	chr4	106156859	106156871	13	0	100.0
chr4:106156917-106156928	chr4	106156917	106156928	12	0	100.0
chr4:106156929-106156941	chr4	106156929	106156941	13	0	100.0
chr4:106156945-106156956	chr4	106156945	106156956	12	0	100.0
chr4:106156987-106156998	chr4	106156987	106156998	12	0	100.0
chr4:106157048-106157059	chr4	106157048	106157059	12	0	100.0
chr4:106157063-106157074	chr4	106157063	106157074	12	0	100.0
chr4:106157161-106157172	chr4	106157161	106157172	12	0	100.0
chr4:106157180-106157191	chr4	106157180	106157191	12	0	100.0
chr4:106157200-106157213	chr4	106157200	106157213	14	0	100.0
chr4:106157215-106157229	chr4	106157215	106157229	15	0	100.0
chr4:106157316-106157327	chr4	106157316	106157327	12	0	100.0
chr4:106157320-106157331	chr4	106157320	106157331	12	0	100.0
chr4:106157334-106157346	chr4	106157334	106157346	13	0	100.0
chr4:106157340-106157357	chr4	106157340	106157357	18	0	100.0
chr4:106157398-106157409	chr4	106157398	106157409	12	0	100.0
chr4:106157440-106157451	chr4	106157440	106157451	12	0	100.0
chr4:106157550-106157561	chr4	106157550	106157561	12	0	100.0
chr4:106157577-106157588	chr4	106157577	106157588	12	0	100.0
chr4:106157578-106157589	chr4	106157578	106157589	12	0	100.0
chr4:106157581-106157595	chr4	106157581	106157595	15	0	100.0
chr4:106157639-106157651	chr4	106157639	106157651	13	0	100.0
chr4:106157661-106157672	chr4	106157661	106157672	12	0	100.0
chr4:106157692-106157703	chr4	106157692	106157703	12	0	100.0
chr4:106157785-106157796	chr4	106157785	106157796	12	0	100.0
chr4:106157809-106157832	chr4	106157809	106157832	24	0	100.0
chr4:106157839-106157850	chr4	106157839	106157850	12	0	100.0
chr4:106157849-106157879	chr4	106157849	106157879	31	0	100.0
chr4:106157857-106157868	chr4	106157857	106157868	12	0	100.0
chr4:106157920-106157931	chr4	106157920	106157931	12	0	100.0
chr4:106157932-106157943	chr4	106157932	106157943	12	0	100.0
chr4:106157971-106157982	chr4	106157971	106157982	12	0	100.0
chr4:106157992-106158003	chr4	106157992	106158003	8	4	66.6666666667

chr4:106158193-106158204	chr4	106158193	106158204	12	0	100.0
chr4:106158195-106158207	chr4	106158195	106158207	13	0	100.0
chr4:106158231-106158243	chr4	106158231	106158243	13	0	100.0
chr4:106158279-106158291	chr4	106158279	106158291	13	0	100.0
chr4:106158373-106158384	chr4	106158373	106158384	12	0	100.0
chr4:106158403-106158415	chr4	106158403	106158415	13	0	100.0
chr4:106158405-106158417	chr4	106158405	106158417	13	0	100.0
chr4:106158406-106158417	chr4	106158406	106158417	12	0	100.0
chr4:106158436-106158447	chr4	106158436	106158447	12	0	100.0
chr4:106158453-106158464	chr4	106158453	106158464	12	0	100.0
chr4:106158456-106158468	chr4	106158456	106158468	13	0	100.0
chr4:106158503-106158515	chr4	106158503	106158515	13	0	100.0
chr4:106162492-106162504	chr4	106162492	106162504	13	0	100.0
chr4:106162505-106162530	chr4	106162505	106162530	26	0	100.0
chr4:106162522-106162533	chr4	106162522	106162533	12	0	100.0
chr4:106162547-106162558	chr4	106162547	106162558	12	0	100.0
chr4:106162579-106162590	chr4	106162579	106162590	12	0	100.0
chr4:106162580-106162591	chr4	106162580	106162591	12	0	100.0
chr4:106164014-106164025	chr4	106164014	106164025	12	0	100.0
chr4:106164023-106164034	chr4	106164023	106164034	12	0	100.0
chr4:106164065-106164076	chr4	106164065	106164076	12	0	100.0
chr4:106164079-106164090	chr4	106164079	106164090	12	0	100.0
chr4:106164079-106164091	chr4	106164079	106164091	13	0	100.0
chr4:106164745-106164756	chr4	106164745	106164756	12	0	100.0
chr4:106164761-106164772	chr4	106164761	106164772	12	0	100.0
chr4:106164763-106164774	chr4	106164763	106164774	12	0	100.0
chr4:106164772-106164783	chr4	106164772	106164783	12	0	100.0
chr4:106164781-106164792	chr4	106164781	106164792	12	0	100.0
chr4:106164788-106164799	chr4	106164788	106164799	12	0	100.0
chr4:106164855-106164870	chr4	106164855	106164870	16	0	100.0
chr4:106164860-106164871	chr4	106164860	106164871	12	0	100.0
chr4:106164888-106164900	chr4	106164888	106164900	13	0	100.0
chr4:106164896-106164917	chr4	106164896	106164917	22	0	100.0
chr4:106164908-106164919	chr4	106164908	106164919	12	0	100.0
chr4:106164922-106164933	chr4	106164922	106164933	12	0	100.0
chr4:106180772-106180783	chr4	106180772	106180783	12	0	100.0
chr4:106180784-106180795	chr4	106180784	106180795	12	0	100.0
chr4:106180789-106180800	chr4	106180789	106180800	12	0	100.0
chr4:106180790-106180801	chr4	106180790	106180801	12	0	100.0
chr4:106180811-106180822	chr4	106180811	106180822	12	0	100.0
chr4:106180817-106180828	chr4	106180817	106180828	12	0	100.0
chr4:106180824-106180835	chr4	106180824	106180835	12	0	100.0
chr4:106180846-106180857	chr4	106180846	106180857	12	0	100.0
chr4:106180856-106180867	chr4	106180856	106180867	12	0	100.0
chr4:106180859-106180870	chr4	106180859	106180870	12	0	100.0
chr4:106180864-106180875	chr4	106180864	106180875	12	0	100.0
chr4:106180920-106180931	chr4	106180920	106180931	12	0	100.0
chr4:106180922-106180933	chr4	106180922	106180933	12	0	100.0
chr4:106182951-106182963	chr4	106182951	106182963	13	0	100.0
chr4:106182975-106182987	chr4	106182975	106182987	13	0	100.0
chr4:106182986-106182997	chr4	106182986	106182997	12	0	100.0
chr4:106183000-106183011	chr4	106183000	106183011	12	0	100.0
chr4:106190787-106190798	chr4	106190787	106190798	12	0	100.0
chr4:106190792-106190803	chr4	106190792	106190803	12	0	100.0
chr4:106190798-106190809	chr4	106190798	106190809	12	0	100.0
chr4:106190812-106190824	chr4	106190812	106190824	13	0	100.0
chr4:106190813-106190824	chr4	106190813	106190824	12	0	100.0
chr4:106190822-106190833	chr4	106190822	106190833	12	0	100.0
chr4:106190842-106190853	chr4	106190842	106190853	12	0	100.0
chr4:106190843-106190857	chr4	106190843	106190857	15	0	100.0
chr4:106190849-106190860	chr4	106190849	106190860	12	0	100.0
chr4:106190854-106190865	chr4	106190854	106190865	12	0	100.0
chr4:106190861-106190872	chr4	106190861	106190872	12	0	100.0
chr4:106190867-106190878	chr4	106190867	106190878	12	0	100.0
chr4:106193725-106193736	chr4	106193725	106193736	12	0	100.0
chr4:106193788-106193799	chr4	106193788	106193799	12	0	100.0
chr4:106193795-106193807	chr4	106193795	106193807	13	0	100.0
chr4:106193796-106193808	chr4	106193796	106193808	13	0	100.0
chr4:106193838-106193849	chr4	106193838	106193849	12	0	100.0
chr4:106193886-106193897	chr4	106193886	106193897	12	0	100.0
chr4:106193911-106193922	chr4	106193911	106193922	12	0	100.0
chr4:106193925-106193936	chr4	106193925	106193936	12	0	100.0
chr4:106193989-106194000	chr4	106193989	106194000	12	0	100.0
chr4:106193990-106194004	chr4	106193990	106194004	15	0	100.0
chr4:106193993-106194005	chr4	106193993	106194005	13	0	100.0
chr4:106194006-106194017	chr4	106194006	106194017	12	0	100.0
chr4:106194007-106194019	chr4	106194007	106194019	13	0	100.0
chr4:106196202-106196214	chr4	106196202	106196214	13	0	100.0
chr4:106196207-106196218	chr4	106196207	106196218	12	0	100.0
chr4:106196253-106196265	chr4	106196253	106196265	13	0	100.0
chr4:106196255-106196266	chr4	106196255	106196266	12	0	100.0
chr4:106196270-106196281	chr4	106196270	106196281	12	0	100.0
chr4:106196279-106196290	chr4	106196279	106196290	12	0	100.0
chr4:106196392-106196403	chr4	106196392	106196403	12	0	100.0
chr4:106196481-106196492	chr4	106196481	106196492	12	0	100.0
chr4:106196482-106196494	chr4	106196482	106196494	13	0	100.0
chr4:106196483-106196495	chr4	106196483	106196495	13	0	100.0
chr4:106196489-106196500	chr4	106196489	106196500	12	0	100.0
chr4:106196621-106196632	chr4	106196621	106196632	12	0	100.0
chr4:106196706-106196717	chr4	106196706	106196717	12	0	100.0

chr4:106196717-106196733	chr4	106196717	106196733	17	0	100.0
chr4:106196719-106196730	chr4	106196719	106196730	12	0	100.0
chr4:106196768-106196779	chr4	106196768	106196779	12	0	100.0
chr4:106196828-106196839	chr4	106196828	106196839	12	0	100.0
chr4:106197108-106197119	chr4	106197108	106197119	12	0	100.0
chr4:106197163-106197174	chr4	106197163	106197174	12	0	100.0
chr4:106197266-106197277	chr4	106197266	106197277	12	0	100.0
chr4:106197270-106197281	chr4	106197270	106197281	12	0	100.0
chr4:106197276-106197287	chr4	106197276	106197287	12	0	100.0
chr4:106197279-106197290	chr4	106197279	106197290	12	0	100.0
chr4:106197296-106197307	chr4	106197296	106197307	12	0	100.0
chr4:106197344-106197355	chr4	106197344	106197355	12	0	100.0
chr4:106197354-106197365	chr4	106197354	106197365	12	0	100.0
chr4:106197357-106197368	chr4	106197357	106197368	12	0	100.0
chr4:106197397-106197408	chr4	106197397	106197408	12	0	100.0
chr4:153241633-153241643	chr4	153241633	153241643	11	0	100.0
chr5:149450099-149450110	chr5	149450099	149450110	12	0	100.0
chr5:149488626-149488636	chr5	149488626	149488636	11	0	100.0
chr5:170819940-170819951	chr5	170819940	170819951	12	0	100.0
chr5:170827186-170827197	chr5	170827186	170827197	12	0	100.0
chr5:170827186-170827198	chr5	170827186	170827198	13	0	100.0
chr5:170837537-170837549	chr5	170837537	170837549	13	0	100.0
chr5:170837538-170837550	chr5	170837538	170837550	13	0	100.0
chr5:170837539-170837551	chr5	170837539	170837551	13	0	100.0
chr5:170837542-170837553	chr5	170837542	170837553	12	0	100.0
chr5:170837545-170837557	chr5	170837545	170837557	13	0	100.0
chr7:101454359-101454369	chr7	101454359	101454369	0	11	0.0
BRAF	chr7	140426293	140426316	24	0	100.0
BRAF	chr7	140434396	140434570	156	19	89.1428571429
BRAF	chr7	140439611	140439746	136	0	100.0
BRAF	chr7	140449086	140449218	133	0	100.0
BRAF	chr7	140453074	140453193	120	0	100.0
chr7:140453129-140453139	chr7	140453129	140453139	11	0	100.0
chr7:140453131-140453141	chr7	140453131	140453141	11	0	100.0
chr7:140453140-140453150	chr7	140453140	140453150	11	0	100.0
chr7:140453144-140453154	chr7	140453144	140453154	11	0	100.0
chr7:140453150-140453160	chr7	140453150	140453160	11	0	100.0
chr7:140453188-140453198	chr7	140453188	140453198	11	0	100.0
BRAF	chr7	140453986	140454033	48	0	100.0
BRAF	chr7	140476711	140476888	178	0	100.0
BRAF	chr7	140477790	140477875	86	0	100.0
chr7:140477832-140477842	chr7	140477832	140477842	11	0	100.0
BRAF	chr7	140481375	140481493	119	0	100.0
chr7:140481397-140481407	chr7	140481397	140481407	11	0	100.0
chr7:140481406-140481416	chr7	140481406	140481416	11	0	100.0
BRAF	chr7	140482820	140482957	138	0	100.0
BRAF	chr7	140487347	140487384	38	0	100.0
BRAF	chr7	140494107	140494267	161	0	100.0
BRAF	chr7	140500161	140500281	121	0	100.0
BRAF	chr7	140501211	140501360	149	1	99.3333333333
BRAF	chr7	140507759	140507862	104	0	100.0
BRAF	chr7	140508691	140508795	105	0	100.0
BRAF	chr7	140534408	140534672	221	44	83.3962264151
BRAF	chr7	140549910	140550012	101	2	98.0582524272
BRAF	chr7	140624365	140624503	0	139	0.0
chr7:148504710-148504720	chr7	148504710	148504720	11	0	100.0
chr7:148504755-148504766	chr7	148504755	148504766	12	0	100.0
chr7:148504779-148504790	chr7	148504779	148504790	12	0	100.0
chr7:148504789-148504800	chr7	148504789	148504800	12	0	100.0
chr7:148504790-148504801	chr7	148504790	148504801	12	0	100.0
chr7:148504791-148504802	chr7	148504791	148504802	12	0	100.0
chr7:148504793-148504804	chr7	148504793	148504804	12	0	100.0
chr7:148504794-148504805	chr7	148504794	148504805	12	0	100.0
chr7:148504848-148504858	chr7	148504848	148504858	11	0	100.0
chr7:148506156-148506167	chr7	148506156	148506167	12	0	100.0
chr7:148506161-148506172	chr7	148506161	148506172	12	0	100.0
chr7:148506422-148506433	chr7	148506422	148506433	12	0	100.0
chr7:148506471-148506482	chr7	148506471	148506482	12	0	100.0
chr7:148507429-148507442	chr7	148507429	148507442	14	0	100.0
chr7:148507469-148507480	chr7	148507469	148507480	12	0	100.0
chr7:148507470-148507481	chr7	148507470	148507481	12	0	100.0
chr7:148508710-148508721	chr7	148508710	148508721	12	0	100.0
chr7:148511070-148511081	chr7	148511070	148511081	12	0	100.0
chr7:148511143-148511154	chr7	148511143	148511154	12	0	100.0
chr7:148511183-148511194	chr7	148511183	148511194	12	0	100.0
chr7:148511188-148511199	chr7	148511188	148511199	12	0	100.0
chr7:148513770-148513781	chr7	148513770	148513781	12	0	100.0
chr7:148514315-148514326	chr7	148514315	148514326	12	0	100.0
chr7:148514451-148514541	chr7	148514451	148514541	91	0	100.0
chr7:148514983-148514997	chr7	148514983	148514997	15	0	100.0
chr7:148515133-148515157	chr7	148515133	148515157	25	0	100.0
chr7:148516679-148516691	chr7	148516679	148516691	13	0	100.0
chr7:148523566-148523577	chr7	148523566	148523577	12	0	100.0
chr7:148523585-148523596	chr7	148523585	148523596	12	0	100.0
chr7:148523611-148523622	chr7	148523611	148523622	12	0	100.0
chr7:148524259-148524270	chr7	148524259	148524270	12	0	100.0
chr7:148525832-148525843	chr7	148525832	148525843	12	0	100.0
chr7:148525908-148525919	chr7	148525908	148525919	12	0	100.0
chr7:148525967-148525978	chr7	148525967	148525978	12	0	100.0
chr7:148526863-148526874	chr7	148526863	148526874	12	0	100.0

chr7:148526898-148526920	chr7	148526898	148526920	23	0	100.0
chr7:148529736-148529747	chr7	148529736	148529747	12	0	100.0
chr7:148543572-148543595	chr7	148543572	148543595	24	0	100.0
chr7:148543615-148543626	chr7	148543615	148543626	12	0	100.0
chr7:151845411-151845422	chr7	151845411	151845422	12	0	100.0
chr7:151949057-151949068	chr7	151949057	151949068	12	0	100.0
chr7:151949710-151949721	chr7	151949710	151949721	12	0	100.0
chr8:117859923-117859934	chr8	117859923	117859934	12	0	100.0
chr8:117861264-117861275	chr8	117861264	117861275	12	0	100.0
chr8:117862895-117862907	chr8	117862895	117862907	13	0	100.0
chr8:117864207-117864218	chr8	117864207	117864218	12	0	100.0
chr8:117868506-117868518	chr8	117868506	117868518	13	0	100.0
chr8:117874076-117874088	chr8	117874076	117874088	13	0	100.0
chr8:117875379-117875391	chr8	117875379	117875391	13	0	100.0
chr8:117875403-117875415	chr8	117875403	117875415	13	0	100.0
chr8:117875467-117875478	chr8	117875467	117875478	12	0	100.0
chr8:117878818-117878829	chr8	117878818	117878829	12	0	100.0
chr8:117878851-117878863	chr8	117878851	117878863	13	0	100.0
chr8:117878898-117878910	chr8	117878898	117878910	13	0	100.0
chr8:117878909-117878920	chr8	117878909	117878920	12	0	100.0
MYC	chr8	128748839	128748869	31	0	100.0
MYC	chr8	128750493	128751265	657	116	84.9935316947
chr8:128750561-128750571	chr8	128750561	128750571	11	0	100.0
chr8:128750679-128750689	chr8	128750679	128750689	10	1	90.9090909091
chr8:128750682-128750692	chr8	128750682	128750692	11	0	100.0
MYC	chr8	128752641	128753204	487	77	86.3475177305
chr8:128752705-128752715	chr8	128752705	128752715	11	0	100.0
chr8:137051381-137051391	chr8	137051381	137051391	11	0	100.0
chr9:5054868-5054879	chr9	5054868	5054879	12	0	100.0
chr9:5070020-5070032	chr9	5070020	5070032	13	0	100.0
chr9:5073764-5073775	chr9	5073764	5073775	12	0	100.0
chr9:5078355-5078366	chr9	5078355	5078366	12	0	100.0
chr9:5078356-5078367	chr9	5078356	5078367	12	0	100.0
chr9:5081722-5081733	chr9	5081722	5081733	12	0	100.0
chr9:5081774-5081784	chr9	5081774	5081784	11	0	100.0
chr9:5089720-5089731	chr9	5089720	5089731	12	0	100.0
chr9:139387840-139387850	chr9	139387840	139387850	11	0	100.0
PTEN	chr10	89623706	89623860	123	32	79.3548387097
PTEN	chr10	89623861	89624305	352	93	79.1011235955
PTEN	chr10	89653781	89653866	86	0	100.0
PTEN	chr10	89685269	89685314	46	0	100.0
PTEN	chr10	89690802	89690846	45	0	100.0
PTEN	chr10	89692769	89693008	204	36	85.0
chr10:89711871-89711881	chr10	89711871	89711881	11	0	100.0
PTEN	chr10	89711874	89712016	143	0	100.0
PTEN	chr10	89717609	89717776	168	0	100.0
PTEN	chr10	89720650	89720875	198	28	87.610619469
chr10:89720665-89720675	chr10	89720665	89720675	3	8	27.2727272727
PTEN	chr10	89725043	89725229	173	14	92.513368984
chr10:112342312-112342323	chr10	112342312	112342323	12	0	100.0
chr10:112350855-112350866	chr10	112350855	112350866	12	0	100.0
chr10:112360190-112360201	chr10	112360190	112360201	12	0	100.0
chr10:112360232-112360245	chr10	112360232	112360245	14	0	100.0
chr10:112361832-112361843	chr10	112361832	112361843	12	0	100.0
chr10:129653914-129653924	chr10	129653914	129653924	11	0	100.0
chr11:1568087-1568097	chr11	1568087	1568097	0	11	0.0
chr11:32410660-32410671	chr11	32410660	32410671	12	0	100.0
chr11:32413554-32413565	chr11	32413554	32413565	12	0	100.0
chr11:32413559-32413570	chr11	32413559	32413570	12	0	100.0
chr11:32413560-32413571	chr11	32413560	32413571	12	0	100.0
chr11:32414244-32414255	chr11	32414244	32414255	12	0	100.0
chr11:32414245-32414256	chr11	32414245	32414256	12	0	100.0
chr11:32414256-32414267	chr11	32414256	32414267	12	0	100.0
chr11:32414257-32414268	chr11	32414257	32414268	12	0	100.0
chr11:32417891-32417926	chr11	32417891	32417926	36	0	100.0
chr11:32417901-32417913	chr11	32417901	32417913	13	0	100.0
chr11:32417903-32417915	chr11	32417903	32417915	13	0	100.0
chr11:32417904-32417915	chr11	32417904	32417915	12	0	100.0
chr11:32417905-32417917	chr11	32417905	32417917	13	0	100.0
chr11:32417907-32417919	chr11	32417907	32417919	13	0	100.0
chr11:32417908-32417919	chr11	32417908	32417919	12	0	100.0
chr11:32417929-32417940	chr11	32417929	32417940	12	0	100.0
chr11:32417937-32417948	chr11	32417937	32417948	12	0	100.0
chr11:32417938-32417949	chr11	32417938	32417949	12	0	100.0
chr11:32417941-32417952	chr11	32417941	32417952	12	0	100.0
chr11:32417941-32417953	chr11	32417941	32417953	13	0	100.0
chr11:32456308-32456320	chr11	32456308	32456320	13	0	100.0
chr11:108117848-108117859	chr11	108117848	108117859	12	0	100.0
chr11:108199949-108199960	chr11	108199949	108199960	12	0	100.0
chr11:108201083-108201094	chr11	108201083	108201094	12	0	100.0
chr11:108202163-108202177	chr11	108202163	108202177	15	0	100.0
chr11:108205789-108205800	chr11	108205789	108205800	12	0	100.0
chr11:108213998-108214009	chr11	108213998	108214009	12	0	100.0
chr11:108235834-108235845	chr11	108235834	108235845	12	0	100.0
chr11:118344949-118344960	chr11	118344949	118344960	12	0	100.0
chr11:118375269-118375280	chr11	118375269	118375280	12	0	100.0
chr11:118376959-118376970	chr11	118376959	118376970	12	0	100.0
chr11:118380686-118380746	chr11	118380686	118380746	61	0	100.0
chr11:119142581-119142592	chr11	119142581	119142592	12	0	100.0
chr11:119145534-119145545	chr11	119145534	119145545	12	0	100.0

chr11:119145535-119145546	chr11	119145535	119145546	12	0	100.0
chr11:119145606-119145617	chr11	119145606	119145617	12	0	100.0
chr11:119148530-119148541	chr11	119148530	119148541	12	0	100.0
chr11:119148538-119148557	chr11	119148538	119148557	20	0	100.0
chr11:119148567-119148577	chr11	119148567	119148577	11	0	100.0
chr11:119148868-119148879	chr11	119148868	119148879	12	0	100.0
chr11:119148885-119148896	chr11	119148885	119148896	12	0	100.0
chr11:119148886-119148897	chr11	119148886	119148897	12	0	100.0
chr11:119148904-119148915	chr11	119148904	119148915	12	0	100.0
chr11:119148913-119148924	chr11	119148913	119148924	12	0	100.0
chr11:119148916-119148927	chr11	119148916	119148927	12	0	100.0
chr11:119148918-119148929	chr11	119148918	119148929	12	0	100.0
chr11:119148924-119148935	chr11	119148924	119148935	12	0	100.0
chr11:119148925-119148936	chr11	119148925	119148936	12	0	100.0
chr11:119148960-119148971	chr11	119148960	119148971	12	0	100.0
chr11:119148967-119148978	chr11	119148967	119148978	12	0	100.0
chr11:119148972-119148983	chr11	119148972	119148983	12	0	100.0
chr11:119148976-119148987	chr11	119148976	119148987	12	0	100.0
chr11:119148985-119148996	chr11	119148985	119148996	12	0	100.0
chr11:119149232-119149243	chr11	119149232	119149243	12	0	100.0
chr11:119149233-119149244	chr11	119149233	119149244	12	0	100.0
chr11:119149235-119149246	chr11	119149235	119149246	12	0	100.0
chr11:119149245-119149256	chr11	119149245	119149256	12	0	100.0
chr11:119155726-119155738	chr11	119155726	119155738	13	0	100.0
chr11:119155736-119155747	chr11	119155736	119155747	12	0	100.0
chr11:119168104-119168115	chr11	119168104	119168115	12	0	100.0
chr12:11992082-11992093	chr12	11992082	11992093	12	0	100.0
chr12:12006359-12006370	chr12	12006359	12006370	12	0	100.0
chr12:12006446-12006458	chr12	12006446	12006458	13	0	100.0
chr12:12006491-12006502	chr12	12006491	12006502	12	0	100.0
chr12:12022424-12022436	chr12	12022424	12022436	13	0	100.0
chr12:12022477-12022488	chr12	12022477	12022488	12	0	100.0
chr12:12037468-12037479	chr12	12037468	12037479	12	0	100.0
chr12:12037469-12037480	chr12	12037469	12037480	12	0	100.0
chr12:12037487-12037498	chr12	12037487	12037498	12	0	100.0
chr12:12037501-12037512	chr12	12037501	12037512	12	0	100.0
chr12:12038921-12038932	chr12	12038921	12038932	12	0	100.0
chr12:12043867-12043878	chr12	12043867	12043878	12	0	100.0
chr12:12043872-12043889	chr12	12043872	12043889	18	0	100.0
chr12:22808266-22808276	chr12	22808266	22808276	0	11	0.0
chr12:25378642-25378653	chr12	25378642	25378653	12	0	100.0
chr12:25380202-25380213	chr12	25380202	25380213	12	0	100.0
chr12:25380248-25380259	chr12	25380248	25380259	12	0	100.0
chr12:25380269-25380280	chr12	25380269	25380280	12	0	100.0
chr12:25380270-25380281	chr12	25380270	25380281	12	0	100.0
chr12:25380273-25380284	chr12	25380273	25380284	12	0	100.0
chr12:25380279-25380290	chr12	25380279	25380290	12	0	100.0
chr12:25398214-25398225	chr12	25398214	25398225	12	0	100.0
chr12:25398260-25398271	chr12	25398260	25398271	12	0	100.0
chr12:25398274-25398286	chr12	25398274	25398286	13	0	100.0
chr12:25398275-25398286	chr12	25398275	25398286	12	0	100.0
chr12:25398278-25398289	chr12	25398278	25398289	12	0	100.0
chr12:25398279-25398290	chr12	25398279	25398290	12	0	100.0
chr12:49415636-49415647	chr12	49415636	49415647	12	0	100.0
chr12:49425071-49425083	chr12	49425071	49425083	13	0	100.0
chr12:49427893-49427904	chr12	49427893	49427904	12	0	100.0
chr12:49428188-49428200	chr12	49428188	49428200	13	0	100.0
chr12:49432384-49432395	chr12	49432384	49432395	12	0	100.0
chr12:49433614-49433625	chr12	49433614	49433625	12	0	100.0
chr12:49434945-49434956	chr12	49434945	49434956	12	0	100.0
chr12:49435866-49435877	chr12	49435866	49435877	12	0	100.0
chr12:49436010-49436021	chr12	49436010	49436021	12	0	100.0
chr12:49444370-49444381	chr12	49444370	49444381	12	0	100.0
chr12:49445307-49445331	chr12	49445307	49445331	25	0	100.0
chr12:112884192-112884203	chr12	112884192	112884203	12	0	100.0
chr12:112888156-112888171	chr12	112888156	112888171	16	0	100.0
chr12:112888157-112888168	chr12	112888157	112888168	12	0	100.0
chr12:112888159-112888170	chr12	112888159	112888170	12	0	100.0
chr12:112888160-112888171	chr12	112888160	112888171	12	0	100.0
chr12:112888183-112888194	chr12	112888183	112888194	12	0	100.0
chr12:112888191-112888202	chr12	112888191	112888202	12	0	100.0
chr12:112888192-112888203	chr12	112888192	112888203	12	0	100.0
chr12:112888193-112888204	chr12	112888193	112888204	12	0	100.0
chr12:112888196-112888207	chr12	112888196	112888207	12	0	100.0
chr12:112888204-112888215	chr12	112888204	112888215	12	0	100.0
chr12:112910779-112910790	chr12	112910779	112910790	12	0	100.0
chr12:112926845-112926856	chr12	112926845	112926856	12	0	100.0
chr12:112926882-112926893	chr12	112926882	112926893	12	0	100.0
chr12:112926891-112926902	chr12	112926891	112926902	12	0	100.0
chr13:28589774-28589785	chr13	28589774	28589785	12	0	100.0
chr13:28592606-28592617	chr13	28592606	28592617	12	0	100.0
chr13:28592614-28592625	chr13	28592614	28592625	12	0	100.0
chr13:28592634-28592645	chr13	28592634	28592645	12	0	100.0
chr13:28592635-28592646	chr13	28592635	28592646	12	0	100.0
chr13:28592636-28592647	chr13	28592636	28592647	12	0	100.0
chr13:28608211-28608223	chr13	28608211	28608223	13	0	100.0
chr13:28608212-28608224	chr13	28608212	28608224	13	0	100.0
chr13:28608213-28608225	chr13	28608213	28608225	13	0	100.0
chr13:28608214-28608226	chr13	28608214	28608226	13	0	100.0
chr13:28608223-28608235	chr13	28608223	28608235	13	0	100.0

chr13:28608230-28608242	chr13	28608230	28608242	13	0	100.0
chr13:28608232-28608244	chr13	28608232	28608244	13	0	100.0
chr13:28608236-28608248	chr13	28608236	28608248	13	0	100.0
chr13:28608238-28608250	chr13	28608238	28608250	13	0	100.0
chr13:28608239-28608251	chr13	28608239	28608251	13	0	100.0
chr13:28608242-28608254	chr13	28608242	28608254	13	0	100.0
chr13:28608247-28608259	chr13	28608247	28608259	13	0	100.0
chr13:28608248-28608260	chr13	28608248	28608260	13	0	100.0
chr13:28608249-28608261	chr13	28608249	28608261	13	0	100.0
chr13:28608250-28608262	chr13	28608250	28608262	13	0	100.0
chr13:28608251-28608263	chr13	28608251	28608263	13	0	100.0
chr13:28608252-28608264	chr13	28608252	28608264	13	0	100.0
chr13:28608253-28608265	chr13	28608253	28608265	13	0	100.0
chr13:28608256-28608268	chr13	28608256	28608268	13	0	100.0
chr13:28608262-28608274	chr13	28608262	28608274	13	0	100.0
chr13:28608265-28608277	chr13	28608265	28608277	13	0	100.0
chr13:28608266-28608278	chr13	28608266	28608278	13	0	100.0
chr13:28608270-28608282	chr13	28608270	28608282	13	0	100.0
chr13:28608271-28608283	chr13	28608271	28608283	13	0	100.0
chr13:28608275-28608286	chr13	28608275	28608286	12	0	100.0
chr13:28608280-28608292	chr13	28608280	28608292	13	0	100.0
chr13:28608317-28608329	chr13	28608317	28608329	13	0	100.0
chr13:38359921-38359931	chr13	38359921	38359931	11	0	100.0
chr15:37273286-37273296	chr15	37273286	37273296	11	0	100.0
chr15:90628276-90628287	chr15	90628276	90628287	12	0	100.0
chr15:90631832-90631843	chr15	90631832	90631843	12	0	100.0
chr15:90631928-90631939	chr15	90631928	90631939	12	0	100.0
chr15:90631929-90631940	chr15	90631929	90631940	12	0	100.0
MYH11	chr16	15797847	15797980	134	0	100.0
MYH11	chr16	15802667	15802698	32	0	100.0
MYH11	chr16	15808765	15808938	165	9	94.8275862069
MYH11	chr16	15809020	15809129	110	0	100.0
MYH11	chr16	15810996	15811205	206	4	98.0952380952
chr16:15811017-15811027	chr16	15811017	15811027	11	0	100.0
chr16:15811018-15811028	chr16	15811018	15811028	11	0	100.0
chr16:15811056-15811066	chr16	15811056	15811066	11	0	100.0
chr16:15811057-15811067	chr16	15811057	15811067	11	0	100.0
MYH11	chr16	15812171	15812295	125	0	100.0
chr16:15813071-15813081	chr16	15813071	15813081	11	0	100.0
MYH11	chr16	15813076	15813165	90	0	100.0
MYH11	chr16	15813441	15813570	130	0	100.0
chr16:15813519-15813529	chr16	15813519	15813529	11	0	100.0
MYH11	chr16	15814007	15814169	163	0	100.0
MYH11	chr16	15814695	15814908	197	17	92.0560747664
chr16:15814726-15814736	chr16	15814726	15814736	11	0	100.0
MYH11	chr16	15815278	15815491	208	6	97.1962616822
MYH11	chr16	15818017	15818266	213	37	85.2
chr16:15818135-15818145	chr16	15818135	15818145	11	0	100.0
chr16:15818136-15818146	chr16	15818136	15818146	11	0	100.0
MYH11	chr16	15818503	15818656	154	0	100.0
chr16:15818542-15818552	chr16	15818542	15818552	11	0	100.0
chr16:15818648-15818658	chr16	15818648	15818658	11	0	100.0
MYH11	chr16	15818744	15818849	106	0	100.0
MYH11	chr16	15820704	15820911	197	11	94.7115384615
MYH11	chr16	15826420	15826565	146	0	100.0
MYH11	chr16	15829222	15829435	200	14	93.4579439252
MYH11	chr16	15831305	15831477	173	0	100.0
MYH11	chr16	15832421	15832545	124	1	99.2
MYH11	chr16	15833907	15834045	133	6	95.6834532374
MYH11	chr16	15835319	15835526	190	18	91.3461538462
MYH11	chr16	15835616	15835748	133	0	100.0
MYH11	chr16	15838985	15839094	110	0	100.0
chr16:15839029-15839039	chr16	15839029	15839039	11	0	100.0
MYH11	chr16	15841426	15841587	162	0	100.0
chr16:15841458-15841468	chr16	15841458	15841468	11	0	100.0
MYH11	chr16	15841730	15841800	71	0	100.0
MYH11	chr16	15841903	15842025	123	0	100.0
MYH11	chr16	15843994	15844188	177	18	90.7692307692
MYH11	chr16	15847250	15847365	116	0	100.0
MYH11	chr16	15850197	15850371	159	16	90.8571428571
chr16:15850198-15850208	chr16	15850198	15850208	11	0	100.0
chr16:15850199-15850209	chr16	15850199	15850209	11	0	100.0
MYH11	chr16	15851683	15851857	160	15	91.4285714286
MYH11	chr16	15853432	15853585	154	0	100.0
MYH11	chr16	15854396	15854515	120	0	100.0
MYH11	chr16	15857652	15857748	97	0	100.0
MYH11	chr16	15865425	15865569	145	0	100.0
MYH11	chr16	15869934	15870033	100	0	100.0
chr16:15869953-15869963	chr16	15869953	15869963	11	0	100.0
MYH11	chr16	15872636	15872700	65	0	100.0
MYH11	chr16	15876241	15876334	94	0	100.0
MYH11	chr16	15878554	15878575	22	0	100.0
MYH11	chr16	15880486	15880589	104	0	100.0
MYH11	chr16	15892516	15892544	29	0	100.0
MYH11	chr16	15917111	15917268	158	0	100.0
chr16:15917192-15917202	chr16	15917192	15917202	11	0	100.0
MYH11	chr16	15931764	15932109	308	38	89.0173410405
chr16:63753544-63753554	chr16	63753544	63753554	11	0	100.0
chr16:67063634-67063645	chr16	67063634	67063645	12	0	100.0
chr16:67063711-67063722	chr16	67063711	67063722	12	0	100.0

chr16:67063711-67063723	chr16	67063711	67063723	13	0	100.0
chr17:7573976-7573988	chr17	7573976	7573988	13	0	100.0
chr17:7573981-7573992	chr17	7573981	7573992	12	0	100.0
chr17:7573992-7574004	chr17	7573992	7574004	13	0	100.0
chr17:7574019-7574030	chr17	7574019	7574030	12	0	100.0
chr17:7577026-7577037	chr17	7577026	7577037	12	0	100.0
chr17:7577075-7577086	chr17	7577075	7577086	12	0	100.0
chr17:7577076-7577087	chr17	7577076	7577087	12	0	100.0
chr17:7577087-7577099	chr17	7577087	7577099	13	0	100.0
chr17:7577088-7577099	chr17	7577088	7577099	12	0	100.0
chr17:7577090-7577101	chr17	7577090	7577101	12	0	100.0
chr17:7577094-7577105	chr17	7577094	7577105	12	0	100.0
chr17:7577100-7577111	chr17	7577100	7577111	12	0	100.0
chr17:7577102-7577113	chr17	7577102	7577113	12	0	100.0
chr17:7577114-7577125	chr17	7577114	7577125	12	0	100.0
chr17:7577115-7577126	chr17	7577115	7577126	12	0	100.0
chr17:7577118-7577129	chr17	7577118	7577129	12	0	100.0
chr17:7577136-7577147	chr17	7577136	7577147	12	0	100.0
chr17:7577138-7577149	chr17	7577138	7577149	12	0	100.0
chr17:7577509-7577522	chr17	7577509	7577522	14	0	100.0
chr17:7577530-7577541	chr17	7577530	7577541	12	0	100.0
chr17:7577532-7577543	chr17	7577532	7577543	12	0	100.0
chr17:7577533-7577544	chr17	7577533	7577544	12	0	100.0
chr17:7577541-7577552	chr17	7577541	7577552	12	0	100.0
chr17:7577545-7577556	chr17	7577545	7577556	12	0	100.0
chr17:7577551-7577562	chr17	7577551	7577562	12	0	100.0
chr17:7577553-7577564	chr17	7577553	7577564	12	0	100.0
chr17:7577557-7577568	chr17	7577557	7577568	12	0	100.0
chr17:7577561-7577572	chr17	7577561	7577572	12	0	100.0
chr17:7577562-7577573	chr17	7577562	7577573	12	0	100.0
chr17:7577575-7577586	chr17	7577575	7577586	12	0	100.0
chr17:7578184-7578195	chr17	7578184	7578195	12	0	100.0
chr17:7578197-7578208	chr17	7578197	7578208	12	0	100.0
chr17:7578198-7578209	chr17	7578198	7578209	12	0	100.0
chr17:7578202-7578213	chr17	7578202	7578213	12	0	100.0
chr17:7578204-7578217	chr17	7578204	7578217	14	0	100.0
chr17:7578206-7578217	chr17	7578206	7578217	12	0	100.0
chr17:7578247-7578258	chr17	7578247	7578258	12	0	100.0
chr17:7578254-7578265	chr17	7578254	7578265	12	0	100.0
chr17:7578256-7578267	chr17	7578256	7578267	12	0	100.0
chr17:7578257-7578268	chr17	7578257	7578268	12	0	100.0
chr17:7578259-7578270	chr17	7578259	7578270	12	0	100.0
chr17:7578262-7578273	chr17	7578262	7578273	12	0	100.0
chr17:7578275-7578286	chr17	7578275	7578286	12	0	100.0
chr17:7578364-7578375	chr17	7578364	7578375	12	0	100.0
chr17:7578382-7578393	chr17	7578382	7578393	12	0	100.0
chr17:7578387-7578398	chr17	7578387	7578398	12	0	100.0
chr17:7578388-7578399	chr17	7578388	7578399	12	0	100.0
chr17:7578389-7578400	chr17	7578389	7578400	12	0	100.0
chr17:7578390-7578401	chr17	7578390	7578401	12	0	100.0
chr17:7578397-7578408	chr17	7578397	7578408	12	0	100.0
chr17:7578398-7578409	chr17	7578398	7578409	12	0	100.0
chr17:7578400-7578411	chr17	7578400	7578411	12	0	100.0
chr17:7578407-7578418	chr17	7578407	7578418	12	0	100.0
chr17:7578436-7578447	chr17	7578436	7578447	12	0	100.0
chr17:7578437-7578448	chr17	7578437	7578448	12	0	100.0
chr17:7578448-7578460	chr17	7578448	7578460	13	0	100.0
chr17:7578449-7578460	chr17	7578449	7578460	12	0	100.0
chr17:7578451-7578462	chr17	7578451	7578462	12	0	100.0
chr17:7578451-7578463	chr17	7578451	7578463	13	0	100.0
chr17:7578457-7578468	chr17	7578457	7578468	12	0	100.0
chr17:7578490-7578501	chr17	7578490	7578501	12	0	100.0
chr17:7578497-7578508	chr17	7578497	7578508	12	0	100.0
chr17:7578501-7578512	chr17	7578501	7578512	12	0	100.0
chr17:7578521-7578532	chr17	7578521	7578532	12	0	100.0
chr17:7578528-7578539	chr17	7578528	7578539	12	0	100.0
chr17:7578547-7578558	chr17	7578547	7578558	12	0	100.0
chr17:7578549-7578560	chr17	7578549	7578560	12	0	100.0
chr17:7579368-7579379	chr17	7579368	7579379	12	0	100.0
chr17:7579380-7579391	chr17	7579380	7579391	12	0	100.0
chr17:7579383-7579394	chr17	7579383	7579394	12	0	100.0
chr17:7579408-7579419	chr17	7579408	7579419	12	0	100.0
chr17:7579432-7579444	chr17	7579432	7579444	13	0	100.0
chr17:7579466-7579476	chr17	7579466	7579476	11	0	100.0
chr17:7579504-7579516	chr17	7579504	7579516	13	0	100.0
chr17:7579525-7579536	chr17	7579525	7579536	12	0	100.0
chr17:7579532-7579544	chr17	7579532	7579544	13	0	100.0
chr17:29508736-29508747	chr17	29508736	29508747	12	0	100.0
chr17:29508769-29508779	chr17	29508769	29508779	11	0	100.0
chr17:29528099-29528111	chr17	29528099	29528111	13	0	100.0
chr17:29553450-29553465	chr17	29553450	29553465	16	0	100.0
chr17:29553471-29553483	chr17	29553471	29553483	13	0	100.0
chr17:29553479-29553489	chr17	29553479	29553489	11	0	100.0
chr17:29553575-29553587	chr17	29553575	29553587	13	0	100.0
chr17:29556986-29556997	chr17	29556986	29556997	12	0	100.0
chr17:29559185-29559197	chr17	29559185	29559197	13	0	100.0
chr17:29562741-29562752	chr17	29562741	29562752	12	0	100.0
chr17:29562780-29562791	chr17	29562780	29562791	12	0	100.0
chr17:29579966-29579977	chr17	29579966	29579977	12	0	100.0
chr17:29587408-29587419	chr17	29587408	29587419	12	0	100.0

chr17:29588745-29588756	chr17	29588745	29588756	12	0	100.0
chr17:29588870-29588881	chr17	29588870	29588881	12	0	100.0
chr17:29652879-29652890	chr17	29652879	29652890	12	0	100.0
chr17:29665104-29665115	chr17	29665104	29665115	12	0	100.0
chr17:29670036-29670047	chr17	29670036	29670047	12	0	100.0
chr17:29676131-29676142	chr17	29676131	29676142	12	0	100.0
chr17:29679240-29679250	chr17	29679240	29679250	11	0	100.0
chr17:29679292-29679303	chr17	29679292	29679303	12	0	100.0
chr17:29684308-29684319	chr17	29684308	29684319	12	0	100.0
chr17:29701023-29701034	chr17	29701023	29701034	12	0	100.0
STAT3	chr17	40467762	40467818	57	0	100.0
STAT3	chr17	40468806	40468919	114	0	100.0
STAT3	chr17	40469199	40469242	44	0	100.0
STAT3	chr17	40474299	40474512	205	9	95.7943925234
chr17:40474414-40474424	chr17	40474414	40474424	11	0	100.0
STAT3	chr17	40475021	40475161	141	0	100.0
chr17:40475027-40475037	chr17	40475027	40475037	11	0	100.0
STAT3	chr17	40475277	40475372	96	0	100.0
STAT3	chr17	40475590	40475643	54	0	100.0
STAT3	chr17	40476728	40476864	137	0	100.0
STAT3	chr17	40476980	40477079	100	0	100.0
STAT3	chr17	40478133	40478217	85	0	100.0
STAT3	chr17	40481427	40481475	49	0	100.0
STAT3	chr17	40481571	40481665	95	0	100.0
STAT3	chr17	40481764	40481794	31	0	100.0
STAT3	chr17	40483489	40483549	61	0	100.0
STAT3	chr17	40485690	40485783	94	0	100.0
STAT3	chr17	40485908	40486067	160	0	100.0
STAT3	chr17	40489452	40489604	153	0	100.0
STAT3	chr17	40489780	40489875	96	0	100.0
STAT3	chr17	40490748	40490830	83	0	100.0
STAT3	chr17	40491331	40491427	97	0	100.0
STAT3	chr17	40497576	40497675	100	0	100.0
STAT3	chr17	40498586	40498731	146	0	100.0
STAT3	chr17	40500406	40500534	129	0	100.0
PPM1D	chr17	58677775	58678247	343	130	72.5158562368
chr17:58678032-58678042	chr17	58678032	58678042	11	0	100.0
PPM1D	chr17	58700881	58701110	207	23	90.0
PPM1D	chr17	58711213	58711338	126	0	100.0
PPM1D	chr17	58725252	58725443	185	7	96.3541666667
PPM1D	chr17	58733959	58734202	209	35	85.6557377049
PPM1D	chr17	58740355	58740913	472	87	84.4364937388
chr17:58740434-58740444	chr17	58740434	58740444	11	0	100.0
chr17:58740618-58740628	chr17	58740618	58740628	11	0	100.0
chr17:58740619-58740629	chr17	58740619	58740629	11	0	100.0
chr17:58740804-58740814	chr17	58740804	58740814	11	0	100.0
chr17:74732930-74732964	chr17	74732930	74732964	8	27	22.8571428571
chr17:74732931-74732964	chr17	74732931	74732964	8	26	23.5294117647
chr17:74732950-74732961	chr17	74732950	74732961	5	7	41.6666666667
chr17:74732951-74732961	chr17	74732951	74732961	5	6	45.4545454545
chr17:74732953-74732964	chr17	74732953	74732964	8	4	66.6666666667
chr17:74732953-74732965	chr17	74732953	74732965	9	4	69.2307692308
chr17:74732954-74732964	chr17	74732954	74732964	8	3	72.7272727273
chr17:74732954-74732965	chr17	74732954	74732965	9	3	75.0
chr17:74732954-74732965	chr17	74732954	74732965	9	3	75.0
chr17:74732955-74732965	chr17	74732955	74732965	9	2	81.8181818182
chr18:42531215-42531226	chr18	42531215	42531226	12	0	100.0
chr18:42531901-42531912	chr18	42531901	42531912	12	0	100.0
chr18:42531902-42531913	chr18	42531902	42531913	12	0	100.0
chr18:42531905-42531916	chr18	42531905	42531916	12	0	100.0
chr18:42531907-42531918	chr18	42531907	42531918	12	0	100.0
chr18:42531908-42531919	chr18	42531908	42531919	12	0	100.0
chr18:42531911-42531922	chr18	42531911	42531922	12	0	100.0
chr18:42531913-42531924	chr18	42531913	42531924	12	0	100.0
chr19:13054559-13054621	chr19	13054559	13054621	63	0	100.0
chr19:13054563-13054607	chr19	13054563	13054607	45	0	100.0
chr19:13054596-13054617	chr19	13054596	13054617	22	0	100.0
chr19:13054621-13054633	chr19	13054621	13054633	13	0	100.0
chr19:15755106-15755116	chr19	15755106	15755116	11	0	100.0
chr19:24520426-24520436	chr19	24520426	24520436	11	0	100.0
chr19:33792210-33792260	chr19	33792210	33792260	51	0	100.0
chr19:33792244-33792256	chr19	33792244	33792256	13	0	100.0
chr19:33792359-33792370	chr19	33792359	33792370	12	0	100.0
chr19:33792369-33792380	chr19	33792369	33792380	12	0	100.0
chr19:33792376-33792387	chr19	33792376	33792387	12	0	100.0
chr19:33792377-33792389	chr19	33792377	33792389	13	0	100.0
chr19:33792378-33792390	chr19	33792378	33792390	13	0	100.0
chr19:33792381-33792409	chr19	33792381	33792409	29	0	100.0
chr19:33792404-33792415	chr19	33792404	33792415	12	0	100.0
chr19:33792417-33792428	chr19	33792417	33792428	12	0	100.0
chr19:33792425-33792436	chr19	33792425	33792436	12	0	100.0
chr19:33792439-33792451	chr19	33792439	33792451	13	0	100.0
chr19:33792459-33792470	chr19	33792459	33792470	12	0	100.0
chr19:33792496-33792508	chr19	33792496	33792508	0	13	0.0
chr19:33792497-33792508	chr19	33792497	33792508	0	12	0.0
chr19:33792629-33792641	chr19	33792629	33792641	13	0	100.0
chr19:33792630-33792641	chr19	33792630	33792641	12	0	100.0
chr19:33792663-33792675	chr19	33792663	33792675	2	11	15.3846153846
chr19:33792664-33792675	chr19	33792664	33792675	2	10	16.6666666667
chr19:33792725-33792737	chr19	33792725	33792737	13	0	100.0

chr19:33792726-33792737	chr19	33792726	33792737	12	0	100.0
chr19:33792772-33792783	chr19	33792772	33792783	12	0	100.0
chr19:33792773-33792783	chr19	33792773	33792783	11	0	100.0
chr19:33792877-33792889	chr19	33792877	33792889	13	0	100.0
chr19:33792878-33792889	chr19	33792878	33792889	12	0	100.0
chr19:33792878-33792890	chr19	33792878	33792890	13	0	100.0
chr19:33792879-33792890	chr19	33792879	33792890	12	0	100.0
chr19:33792886-33792898	chr19	33792886	33792898	13	0	100.0
chr19:33792887-33792898	chr19	33792887	33792898	12	0	100.0
chr19:33793023-33793035	chr19	33793023	33793035	13	0	100.0
chr19:33793024-33793035	chr19	33793024	33793035	12	0	100.0
chr19:33793056-33793068	chr19	33793056	33793068	13	0	100.0
chr19:33793057-33793068	chr19	33793057	33793068	12	0	100.0
chr19:33793068-33793079	chr19	33793068	33793079	12	0	100.0
chr19:33793069-33793079	chr19	33793069	33793079	11	0	100.0
chr19:33793120-33793153	chr19	33793120	33793153	22	12	64.7058823529
chr19:33793121-33793153	chr19	33793121	33793153	22	11	66.6666666667
chr19:33793154-33793177	chr19	33793154	33793177	24	0	100.0
chr19:33793155-33793177	chr19	33793155	33793177	23	0	100.0
chr19:33793237-33793258	chr19	33793237	33793258	22	0	100.0
chr19:33793238-33793250	chr19	33793238	33793250	13	0	100.0
chr19:33793238-33793258	chr19	33793238	33793258	21	0	100.0
chr19:33793239-33793250	chr19	33793239	33793250	12	0	100.0
chr19:33793246-33793258	chr19	33793246	33793258	13	0	100.0
chr19:33793247-33793258	chr19	33793247	33793258	12	0	100.0
chr19:33793270-33793284	chr19	33793270	33793284	15	0	100.0
chr19:33793271-33793284	chr19	33793271	33793284	14	0	100.0
chr19:36211654-36211665	chr19	36211654	36211665	12	0	100.0
chr19:56172535-56172546	chr19	56172535	56172546	12	0	100.0
chr19:56173944-56173969	chr19	56173944	56173969	26	0	100.0
chr20:31021243-31021258	chr20	31021243	31021258	16	0	100.0
chr20:31021244-31021255	chr20	31021244	31021255	12	0	100.0
chr20:31021270-31021282	chr20	31021270	31021282	13	0	100.0
chr20:31022228-31022239	chr20	31022228	31022239	12	0	100.0
chr20:31022245-31022275	chr20	31022245	31022275	31	0	100.0
chr20:31022280-31022292	chr20	31022280	31022292	13	0	100.0
chr20:31022282-31022293	chr20	31022282	31022293	12	0	100.0
chr20:31022283-31022294	chr20	31022283	31022294	12	0	100.0
chr20:31022397-31022430	chr20	31022397	31022430	8	26	23.5294117647
chr20:31022435-31022447	chr20	31022435	31022447	13	0	100.0
chr20:31022446-31022458	chr20	31022446	31022458	13	0	100.0
chr20:31022496-31022514	chr20	31022496	31022514	19	0	100.0
chr20:31022500-31022514	chr20	31022500	31022514	15	0	100.0
chr20:31022505-31022517	chr20	31022505	31022517	13	0	100.0
chr20:31022541-31022553	chr20	31022541	31022553	13	0	100.0
chr20:31022542-31022553	chr20	31022542	31022553	12	0	100.0
chr20:31022567-31022579	chr20	31022567	31022579	12	1	92.3076923077
chr20:31022575-31022586	chr20	31022575	31022586	12	0	100.0
chr20:31022586-31022597	chr20	31022586	31022597	12	0	100.0
chr20:31022590-31022607	chr20	31022590	31022607	18	0	100.0
chr20:31022618-31022629	chr20	31022618	31022629	12	0	100.0
chr20:31022629-31022640	chr20	31022629	31022640	12	0	100.0
chr20:31022631-31022642	chr20	31022631	31022642	12	0	100.0
chr20:31022691-31022702	chr20	31022691	31022702	12	0	100.0
chr20:31022699-31022710	chr20	31022699	31022710	12	0	100.0
chr20:31022739-31022750	chr20	31022739	31022750	12	0	100.0
chr20:31022761-31022773	chr20	31022761	31022773	13	0	100.0
chr20:31022772-31022783	chr20	31022772	31022783	12	0	100.0
chr20:31022826-31022837	chr20	31022826	31022837	12	0	100.0
chr20:31022832-31022843	chr20	31022832	31022843	12	0	100.0
chr20:31022841-31022852	chr20	31022841	31022852	12	0	100.0
chr20:31022847-31022858	chr20	31022847	31022858	12	0	100.0
chr20:31022878-31022889	chr20	31022878	31022889	0	12	0.0
chr20:31022893-31022904	chr20	31022893	31022904	1	11	8.33333333333
chr20:31022916-31022927	chr20	31022916	31022927	12	0	100.0
chr20:31022931-31022942	chr20	31022931	31022942	12	0	100.0
chr20:31022943-31022955	chr20	31022943	31022955	13	0	100.0
chr20:31022976-31022987	chr20	31022976	31022987	12	0	100.0
chr20:31022985-31022996	chr20	31022985	31022996	12	0	100.0
chr20:31023102-31023113	chr20	31023102	31023113	12	0	100.0
chr20:31023138-31023149	chr20	31023138	31023149	12	0	100.0
chr20:31023237-31023248	chr20	31023237	31023248	12	0	100.0
chr20:31023282-31023293	chr20	31023282	31023293	12	0	100.0
chr20:31023382-31023393	chr20	31023382	31023393	12	0	100.0
chr20:31023402-31023413	chr20	31023402	31023413	12	0	100.0
chr20:31023609-31023620	chr20	31023609	31023620	12	0	100.0
chr20:31023624-31023635	chr20	31023624	31023635	12	0	100.0
chr20:31023658-31023670	chr20	31023658	31023670	13	0	100.0
chr20:31023691-31023706	chr20	31023691	31023706	16	0	100.0
chr20:31024067-31024078	chr20	31024067	31024078	12	0	100.0
chr20:31024110-31024121	chr20	31024110	31024121	12	0	100.0
chr20:57470734-57470745	chr20	57470734	57470745	12	0	100.0
chr20:57484414-57484425	chr20	57484414	57484425	12	0	100.0
chr20:57484415-57484426	chr20	57484415	57484426	12	0	100.0
chr20:57485389-57485400	chr20	57485389	57485400	12	0	100.0
chr21:36164399-36164409	chr21	36164399	36164409	11	0	100.0
chr21:36164428-36164439	chr21	36164428	36164439	12	0	100.0
chr21:36164429-36164439	chr21	36164429	36164439	11	0	100.0
chr21:36164592-36164610	chr21	36164592	36164610	19	0	100.0
chr21:36164593-36164610	chr21	36164593	36164610	18	0	100.0

chr21:36164598-36164672	chr21	36164598	36164672	75	0	100.0
chr21:36164599-36164672	chr21	36164599	36164672	74	0	100.0
chr21:36164603-36164618	chr21	36164603	36164618	16	0	100.0
chr21:36164604-36164618	chr21	36164604	36164618	15	0	100.0
chr21:36164606-36164618	chr21	36164606	36164618	13	0	100.0
chr21:36164607-36164618	chr21	36164607	36164618	12	0	100.0
chr21:36164658-36164670	chr21	36164658	36164670	13	0	100.0
chr21:36164659-36164670	chr21	36164659	36164670	12	0	100.0
chr21:36164667-36164692	chr21	36164667	36164692	26	0	100.0
chr21:36164668-36164692	chr21	36164668	36164692	25	0	100.0
chr21:36164680-36164692	chr21	36164680	36164692	13	0	100.0
chr21:36164681-36164693	chr21	36164681	36164693	13	0	100.0
chr21:36164713-36164803	chr21	36164713	36164803	21	70	23.0769230769
chr21:36164714-36164803	chr21	36164714	36164803	20	70	22.2222222222
chr21:36164793-36164823	chr21	36164793	36164823	18	13	58.064516129
chr21:36164827-36164839	chr21	36164827	36164839	13	0	100.0
chr21:36164832-36164844	chr21	36164832	36164844	13	0	100.0
chr21:36164845-36164857	chr21	36164845	36164857	13	0	100.0
chr21:36164872-36164884	chr21	36164872	36164884	13	0	100.0
chr21:36164875-36164892	chr21	36164875	36164892	18	0	100.0
chr21:36164876-36164892	chr21	36164876	36164892	17	0	100.0
chr21:36164879-36164890	chr21	36164879	36164890	12	0	100.0
chr21:36164880-36164890	chr21	36164880	36164890	11	0	100.0
chr21:36164881-36164893	chr21	36164881	36164893	13	0	100.0
chr21:36164882-36164893	chr21	36164882	36164893	12	0	100.0
chr21:36171589-36171601	chr21	36171589	36171601	13	0	100.0
chr21:36171601-36171612	chr21	36171601	36171612	12	0	100.0
chr21:36171606-36171618	chr21	36171606	36171618	13	0	100.0
chr21:36171624-36171636	chr21	36171624	36171636	13	0	100.0
chr21:36171692-36171704	chr21	36171692	36171704	13	0	100.0
chr21:36171745-36171756	chr21	36171745	36171756	12	0	100.0
chr21:36206700-36206711	chr21	36206700	36206711	12	0	100.0
chr21:36206756-36206768	chr21	36206756	36206768	13	0	100.0
chr21:36206773-36206797	chr21	36206773	36206797	25	0	100.0
chr21:36206780-36206804	chr21	36206780	36206804	25	0	100.0
chr21:36206796-36206807	chr21	36206796	36206807	12	0	100.0
chr21:36206829-36206841	chr21	36206829	36206841	13	0	100.0
chr21:36206869-36206880	chr21	36206869	36206880	12	0	100.0
chr21:36206884-36206895	chr21	36206884	36206895	12	0	100.0
chr21:36231767-36231778	chr21	36231767	36231778	12	0	100.0
chr21:36231768-36231779	chr21	36231768	36231779	12	0	100.0
chr21:36231776-36231787	chr21	36231776	36231787	12	0	100.0
chr21:36231777-36231788	chr21	36231777	36231788	12	0	100.0
chr21:36231782-36231793	chr21	36231782	36231793	12	0	100.0
chr21:36231785-36231796	chr21	36231785	36231796	12	0	100.0
chr21:36231786-36231797	chr21	36231786	36231797	12	0	100.0
chr21:36231807-36231819	chr21	36231807	36231819	13	0	100.0
chr21:36231815-36231827	chr21	36231815	36231827	13	0	100.0
chr21:36231829-36231841	chr21	36231829	36231841	13	0	100.0
chr21:36231871-36231882	chr21	36231871	36231882	12	0	100.0
chr21:36252846-36252857	chr21	36252846	36252857	12	0	100.0
chr21:36252859-36252870	chr21	36252859	36252870	12	0	100.0
chr21:36252860-36252871	chr21	36252860	36252871	12	0	100.0
chr21:36252863-36252874	chr21	36252863	36252874	12	0	100.0
chr21:36252871-36252882	chr21	36252871	36252882	12	0	100.0
chr21:36252872-36252883	chr21	36252872	36252883	12	0	100.0
chr21:36252910-36252922	chr21	36252910	36252922	13	0	100.0
chr21:36252917-36252954	chr21	36252917	36252954	38	0	100.0
chr21:36252931-36252943	chr21	36252931	36252943	13	0	100.0
chr21:36252934-36252945	chr21	36252934	36252945	12	0	100.0
chr21:36252969-36252981	chr21	36252969	36252981	13	0	100.0
chr21:36252988-36253000	chr21	36252988	36253000	13	0	100.0
chr21:36253002-36253014	chr21	36253002	36253014	13	0	100.0
chr21:36253005-36253016	chr21	36253005	36253016	12	0	100.0
chr21:36259151-36259162	chr21	36259151	36259162	11	1	91.6666666667
chr21:36259152-36259162	chr21	36259152	36259162	11	0	100.0
chr21:36259160-36259171	chr21	36259160	36259171	12	0	100.0
chr21:36259166-36259177	chr21	36259166	36259177	12	0	100.0
chr21:36259167-36259178	chr21	36259167	36259178	12	0	100.0
chr21:36259169-36259181	chr21	36259169	36259181	13	0	100.0
chr21:36259170-36259182	chr21	36259170	36259182	13	0	100.0
chr21:36259173-36259185	chr21	36259173	36259185	13	0	100.0
chr21:36259184-36259195	chr21	36259184	36259195	12	0	100.0
chr21:36259193-36259204	chr21	36259193	36259204	12	0	100.0
chr21:36259198-36259209	chr21	36259198	36259209	12	0	100.0
chr21:36259219-36259230	chr21	36259219	36259230	12	0	100.0
chr21:36259223-36259234	chr21	36259223	36259234	12	0	100.0
chr21:36259231-36259243	chr21	36259231	36259243	13	0	100.0
chr21:36259235-36259247	chr21	36259235	36259247	13	0	100.0
chr21:36259239-36259254	chr21	36259239	36259254	16	0	100.0
ERG	chr21	39739556	39739570	15	0	100.0
ERG	chr21	39755324	39755845	444	78	85.0574712644
chr21:39755727-39755737	chr21	39755727	39755737	11	0	100.0
ERG	chr21	39762916	39762964	49	0	100.0
ERG	chr21	39763580	39763637	58	0	100.0
ERG	chr21	39764297	39764366	70	0	100.0
ERG	chr21	39772283	39772567	246	39	86.3157894737
ERG	chr21	39774478	39774559	82	0	100.0
chr21:39774506-39774516	chr21	39774506	39774516	11	0	100.0
chr21:39774538-39774548	chr21	39774538	39774548	11	0	100.0

ERG	chr21	39775427	39775631	201	4	98.0487804878
ERG	chr21	39795331	39795483	153	0	100.0
ERG	chr21	39817326	39817544	211	8	96.3470319635
ERG	chr21	39870286	39870304	19	0	100.0
ERG	chr21	39947585	39947624	40	0	100.0
chr21:44514766-44514778	chr21	44514766	44514778	13	0	100.0
chr21:44514771-44514782	chr21	44514771	44514782	12	0	100.0
chr21:44514774-44514785	chr21	44514774	44514785	12	0	100.0
chr21:44524450-44524461	chr21	44524450	44524461	12	0	100.0
chr22:29090055-29090066	chr22	29090055	29090066	12	0	100.0
chr22:29090083-29090094	chr22	29090083	29090094	12	0	100.0
chr22:29091776-29091787	chr22	29091776	29091787	12	0	100.0
chr22:29091792-29091803	chr22	29091792	29091803	12	0	100.0
chr22:29099486-29099497	chr22	29099486	29099497	12	0	100.0
chr22:29105987-29105998	chr22	29105987	29105998	12	0	100.0
chr22:29121268-29121279	chr22	29121268	29121279	12	0	100.0
chr22:29130384-29130395	chr22	29130384	29130395	12	0	100.0
chr22:30738581-30738591	chr22	30738581	30738591	11	0	100.0
chrX:15808626-15808638	chrX	15808626	15808638	13	0	100.0
chrX:15809089-15809101	chrX	15809089	15809101	13	0	100.0
chrX:15821813-15821824	chrX	15821813	15821824	12	0	100.0
chrX:15822266-15822278	chrX	15822266	15822278	13	0	100.0
chrX:15827339-15827350	chrX	15827339	15827350	12	0	100.0
chrX:15827383-15827394	chrX	15827383	15827394	12	0	100.0
chrX:15833804-15833815	chrX	15833804	15833815	12	0	100.0
chrX:15833808-15833819	chrX	15833808	15833819	12	0	100.0
chrX:15833936-15833947	chrX	15833936	15833947	12	0	100.0
chrX:15833956-15833967	chrX	15833956	15833967	12	0	100.0
chrX:15834008-15834019	chrX	15834008	15834019	12	0	100.0
chrX:15836735-15836746	chrX	15836735	15836746	12	0	100.0
chrX:15836738-15836754	chrX	15836738	15836754	17	0	100.0
chrX:15836744-15836755	chrX	15836744	15836755	12	0	100.0
chrX:15838325-15838338	chrX	15838325	15838338	14	0	100.0
chrX:15838360-15838370	chrX	15838360	15838370	11	0	100.0
chrX:15838364-15838375	chrX	15838364	15838375	12	0	100.0
chrX:15838368-15838379	chrX	15838368	15838379	12	0	100.0
chrX:15838377-15838390	chrX	15838377	15838390	14	0	100.0
chrX:15840929-15840940	chrX	15840929	15840940	12	0	100.0
chrX:15841224-15841236	chrX	15841224	15841236	13	0	100.0
chrX:39911450-39911462	chrX	39911450	39911462	13	0	100.0
chrX:39911479-39911491	chrX	39911479	39911491	13	0	100.0
chrX:39913259-39913270	chrX	39913259	39913270	12	0	100.0
chrX:39913510-39913522	chrX	39913510	39913522	13	0	100.0
chrX:39913530-39913541	chrX	39913530	39913541	12	0	100.0
chrX:39913581-39913592	chrX	39913581	39913592	12	0	100.0
chrX:39914703-39914715	chrX	39914703	39914715	13	0	100.0
chrX:39916445-39916456	chrX	39916445	39916456	12	0	100.0
chrX:39921517-39921528	chrX	39921517	39921528	12	0	100.0
chrX:39922025-39922037	chrX	39922025	39922037	13	0	100.0
chrX:39922043-39922054	chrX	39922043	39922054	12	0	100.0
chrX:39922116-39922128	chrX	39922116	39922128	13	0	100.0
chrX:39922118-39922129	chrX	39922118	39922129	12	0	100.0
chrX:39922286-39922298	chrX	39922286	39922298	13	0	100.0
chrX:39923053-39923064	chrX	39923053	39923064	12	0	100.0
chrX:39931703-39931714	chrX	39931703	39931714	12	0	100.0
chrX:39931903-39931914	chrX	39931903	39931914	12	0	100.0
chrX:39931997-39932008	chrX	39931997	39932008	12	0	100.0
chrX:39932204-39932215	chrX	39932204	39932215	12	0	100.0
chrX:39932266-39932279	chrX	39932266	39932279	14	0	100.0
chrX:39932589-39932601	chrX	39932589	39932601	13	0	100.0
chrX:39932901-39932911	chrX	39932901	39932911	11	0	100.0
chrX:39932916-39932927	chrX	39932916	39932927	12	0	100.0
chrX:39932927-39932939	chrX	39932927	39932939	13	0	100.0
chrX:39932978-39932989	chrX	39932978	39932989	12	0	100.0
chrX:39932991-39933002	chrX	39932991	39933002	12	0	100.0
chrX:39933727-39933738	chrX	39933727	39933738	12	0	100.0
chrX:39933869-39933880	chrX	39933869	39933880	12	0	100.0
chrX:39933926-39933938	chrX	39933926	39933938	13	0	100.0
chrX:39934202-39934231	chrX	39934202	39934231	30	0	100.0
KDM6A	chrX	44732797	44732958	84	78	51.8518518519
KDM6A	chrX	44733169	44733233	65	0	100.0
KDM6A	chrX	44820528	44820637	110	0	100.0
KDM6A	chrX	44833910	44833960	51	0	100.0
KDM6A	chrX	44870205	44870264	60	0	100.0
KDM6A	chrX	44879854	44879975	118	4	96.7213114754
chrX:44879866-44879876	chrX	44879866	44879876	11	0	100.0
KDM6A	chrX	44894175	44894230	56	0	100.0
KDM6A	chrX	44896899	44896934	36	0	100.0
chrX:44896916-44896926	chrX	44896916	44896926	11	0	100.0
KDM6A	chrX	44910953	44911047	93	2	97.8947368421
KDM6A	chrX	44913073	44913200	128	0	100.0
KDM6A	chrX	44918250	44918349	100	0	100.0
KDM6A	chrX	44918491	44918711	192	29	86.8778280543
chrX:44918509-44918519	chrX	44918509	44918519	11	0	100.0
KDM6A	chrX	44919266	44919401	136	0	100.0
KDM6A	chrX	44919853	44920009	157	0	100.0
KDM6A	chrX	44920568	44920664	97	0	100.0
KDM6A	chrX	44921891	44921993	103	0	100.0
KDM6A	chrX	44922666	44923062	359	38	90.4282115869
chrX:44922995-44923005	chrX	44922995	44923005	11	0	100.0

KDM6A	chrX	44928823	44929602	659	121	84.4871794872
chrX:44929072-44929082	chrX	44929072	44929082	11	0	100.0
chrX:44929105-44929115	chrX	44929105	44929115	11	0	100.0
KDM6A	chrX	44935941	44936071	131	0	100.0
KDM6A	chrX	44937644	44937750	107	0	100.0
KDM6A	chrX	44938390	44938596	186	21	89.8550724638
chrX:44938558-44938568	chrX	44938558	44938568	11	0	100.0
KDM6A	chrX	44941820	44941885	61	5	92.4242424242
KDM6A	chrX	44941959	44942034	76	0	100.0
KDM6A	chrX	44942704	44942853	147	3	98.0
chrX:44942747-44942757	chrX	44942747	44942757	11	0	100.0
chrX:44942750-44942760	chrX	44942750	44942760	11	0	100.0
chrX:44942755-44942765	chrX	44942755	44942765	11	0	100.0
KDM6A	chrX	44945109	44945224	116	0	100.0
KD	chrX	44948987	44949175	175	14	92.5925925926
KDM6A	chrX	44949967	44950109	143	0	100.0
KDM6A	chrX	44966654	44966781	128	0	100.0
chrX:44969318-44969328	chrX	44969318	44969328	11	0	100.0
KDM6A	chrX	44969323	44969494	171	1	99.4186046512
chrX:44969400-44969410	chrX	44969400	44969410	11	0	100.0
KDM6A	chrX	44970626	44970656	31	0	100.0
chrX:47066324-47066334	chrX	47066324	47066334	11	0	100.0
chrX:53430492-53430503	chrX	53430492	53430503	12	0	100.0
chrX:53432051-53432063	chrX	53432051	53432063	13	0	100.0
chrX:53432195-53432208	chrX	53432195	53432208	14	0	100.0
chrX:53440024-53440035	chrX	53440024	53440035	12	0	100.0
chrX:53441935-53441946	chrX	53441935	53441946	12	0	100.0
chrX:123164971-123164982	chrX	123164971	123164982	12	0	100.0
chrX:123171369-123171380	chrX	123171369	123171380	12	0	100.0
chrX:123171387-123171398	chrX	123171387	123171398	12	0	100.0
chrX:123176490-123176501	chrX	123176490	123176501	12	0	100.0
chrX:123179131-123179143	chrX	123179131	123179143	13	0	100.0
chrX:123179191-123179202	chrX	123179191	123179202	12	0	100.0
chrX:123179196-123179208	chrX	123179196	123179208	13	0	100.0
chrX:123179214-123179225	chrX	123179214	123179225	12	0	100.0
chrX:123181276-123181287	chrX	123181276	123181287	12	0	100.0
chrX:123190058-123190070	chrX	123190058	123190070	13	0	100.0
chrX:123195647-123195659	chrX	123195647	123195659	13	0	100.0
chrX:123196749-123196761	chrX	123196749	123196761	13	0	100.0
chrX:123197038-123197049	chrX	123197038	123197049	12	0	100.0
chrX:123197710-123197721	chrX	123197710	123197721	12	0	100.0
chrX:123197735-123197747	chrX	123197735	123197747	13	0	100.0
chrX:123197776-123197788	chrX	123197776	123197788	13	0	100.0
chrX:123200046-123200057	chrX	123200046	123200057	12	0	100.0
chrX:123202450-123202461	chrX	123202450	123202461	12	0	100.0
chrX:123202498-123202509	chrX	123202498	123202509	12	0	100.0
chrX:123210294-123210306	chrX	123210294	123210306	13	0	100.0
chrX:123211807-123211818	chrX	123211807	123211818	12	0	100.0
chrX:123211828-123211842	chrX	123211828	123211842	15	0	100.0
chrX:123211864-123211876	chrX	123211864	123211876	13	0	100.0
chrX:123217282-123217293	chrX	123217282	123217293	12	0	100.0
chrX:123220434-123220445	chrX	123220434	123220445	12	0	100.0
chrX:123220467-123220479	chrX	123220467	123220479	13	0	100.0
chrX:123220470-123220481	chrX	123220470	123220481	12	0	100.0
chrX:123220609-123220620	chrX	123220609	123220620	12	0	100.0
chrX:123224599-123224638	chrX	123224599	123224638	40	0	100.0
chrX:123229234-123229245	chrX	123229234	123229245	12	0	100.0
chrX:133511700-133511722	chrX	133511700	133511722	23	0	100.0
chrX:133511706-133511717	chrX	133511706	133511717	12	0	100.0
chrX:133511727-133511738	chrX	133511727	133511738	12	0	100.0
chrX:133527604-133527616	chrX	133527604	133527616	13	0	100.0
chrX:133527659-133527670	chrX	133527659	133527670	12	0	100.0
chrX:133527977-133527988	chrX	133527977	133527988	12	0	100.0
chrX:133547934-133547945	chrX	133547934	133547945	12	0	100.0
chrX:133547976-133547987	chrX	133547976	133547987	12	0	100.0
chrX:133549039-133549050	chrX	133549039	133549050	12	0	100.0
chrX:133549062-133549073	chrX	133549062	133549073	12	0	100.0
chrX:133549130-133549141	chrX	133549130	133549141	12	0	100.0
chrX:133549131-133549142	chrX	133549131	133549142	12	0	100.0
chrX:133551263-133551274	chrX	133551263	133551274	12	0	100.0
chrX:133551279-133551291	chrX	133551279	133551291	13	0	100.0
chrX:133551299-133551310	chrX	133551299	133551310	12	0	100.0
chrX:133551306-133551317	chrX	133551306	133551317	12	0	100.0
chrX:133551316-133551327	chrX	133551316	133551327	12	0	100.0
chrX:133551325-133551336	chrX	133551325	133551336	12	0	100.0
chrX:133559259-133559270	chrX	133559259	133559270	12	0	100.0
chrX:133559280-133559291	chrX	133559280	133559291	12	0	100.0

Supplementary Table 6. Differential expression analysis between malignant B-cell subclusters.

p_val	avg_log2FC	pct.1	pct.2	p_val_adj	cluster	gene
0	0,98795229	0,893	0,601	0	Intermediate DZ/LZ GC B-cell cluster	*BLK*
0	0,55133615	0,884	0,754	0	Intermediate DZ/LZ GC B-cell cluster	*CD79B*
0	0,56723795	0,905	0,697	0	Intermediate DZ/LZ GC B-cell cluster	*LYN*
0	0,5402	0,634	0,327	0	Intermediate DZ/LZ GC B-cell cluster	*CD38*
0	0,73345622	0,949	0,754	0	Intermediate DZ/LZ GC B-cell cluster	*ARID1B*
2,709E-238	0,62953065	0,49	0,257	8,034E-234	Intermediate DZ/LZ GC B-cell cluster	*IL7*
1,183E-227	0,37611122	0,809	0,578	3,51E-223	Intermediate DZ/LZ GC B-cell cluster	*ATM*
9,991E-174	0,34337816	0,657	0,441	2,963E-169	Intermediate DZ/LZ GC B-cell cluster	*CREBBP*
3,825E-154	0,28632331	0,614	0,408	1,135E-149	Intermediate DZ/LZ GC B-cell cluster	*EP300*
2,817E-138	0,38886574	0,847	0,648	8,354E-134	Intermediate DZ/LZ GC B-cell cluster	*CARD11*
3,264E-126	0,31576407	0,946	0,806	9,681E	Intermediate DZ/LZ GC B-cell cluster	*PLCG2*
1,919E-260	0,28426339	0,316	0,072	5,693E-256	Activated B-cell cluster	*TNFRSF1B*
1,087E-231	0,6709442	0,847	0,521	3,225E-227	Activated B-cell cluster	*HSPD1*
8,465E-196	0,51914482	0,995	0,956	2,511E-191	Activated B-cell cluster	*RPS24*
5,817E-153	0,6125677	0,748	0,451	1,725E-148	Activated B-cell cluster	*NFKB1*
3,481E-149	0,44502167	0,976	0,895	1,032E-144	Activated B-cell cluster	*RPL27A*
2,048E-142	0,57210531	0,68	0,381	6,074E-138	Activated B-cell cluster	*CD40*
1,314E-115	0,4054458	0,67	0,394	3,897E-111	Activated B-cell cluster	*PRMT1*
1,5626E-81	0,30330071	0,992	0,953	4,6346E-77	Activated B-cell cluster	*RPL18A*
4,136E-304	0,76405217	0,776	0,336	1,227E-299	Pre-plasma cell cluster	*ITGB1*
0	0,49885217	0,443	0,111	0	Pre-plasma cell cluster	*SCARB2*
0	1,4202427	0,824	0,158	0	Pre-plasma cell cluster	*CDKN2A*
0	0,25083826	0,197	0,018	0	Pre-plasma cell cluster	*FGF9*
0	0,68379856	0,469	0,064	0	Pre-plasma cell cluster	*FGFR1*
0	0,33533263	0,667	0,211	0	Pre-plasma cell cluster	*S100A4*
3,39E-299	0,66764657	0,996	0,944	1,005E-294	Pre-plasma cell cluster	*RPL7A*
4,192E	0,45310335	0,975	0,88	1,243E-157	Pre-plasma cell cluster	*RPL4*
7,513E-60	0,26588593	0,995	0,966	2,2283E-55	Pre-plasma cell cluster	*RPL32*
1,694E-192	1,32177924	0,944	0,854	5,024E-188	DZ GC B-cell cluster	*BTG1*
1,253E-104	0,57879793	0,981	0,908	3,715E-100	DZ GC B-cell cluster	*HMGB1*
3,4455E-89	0,75166231	0,861	0,687	1,0219E-84	DZ GC B-cell cluster	*BTG2*
3,1475E-29	0,42561357	0,338	0,215	9,3351E-25	DZ GC B-cell cluster	*TGIF1*
1,7711E-21	0,34605648	0,478	0,366	5,253E-17	DZ GC B-cell cluster	*ITGB1*
0	0,87357579	0,683	0,141	0	LZ GC B-cell cluster	*IL2RA*
0	1,36504434	0,868	0,137	0	LZ GC B-cell cluster	*IL4I1*
0	0,64001157	0,566	0,1	0	LZ GC B-cell cluster	*IL15RA*
1,317E-264	0,95443546	0,893	0,336	3,908E-260	LZ GC B-cell cluster	*IL21R*
1,942E-205	0,90928555	0,904	0,445	5,76E-201	LZ GC B-cell cluster	*STAT3*
6,16E-159	0,40802311	0,569	0,179	1,827E-154	LZ GC B-cell cluster	*IRF7*
4,495E-145	1,19238179	0,966	0,434	1,333E-140	Pre-memory B-cell cluster	*JAK2*
2,245E-79	0,68929301	1	0,969	6,6584E-75	Pre-memory B-cell cluster	*CD74*
1,8199E-67	0,50076544	0,877	0,454	5,3978E-63	Pre-memory B-cell cluster	*STAT3*
9,3313E-60	0,52799989	0,313	0,089	2,7676E-55	Pre-memory B-cell cluster	*TNFRSF13B*
4,3835E-27	0,25329553	0,79	0,46	1,3001E-22	Pre-memory B-cell cluster	*CD22*
